# Differential root and shoot magnetoresponses in *Arabidopsis thaliana*

**DOI:** 10.1038/s41598-021-88695-6

**Published:** 2021-04-28

**Authors:** Ivan A. Paponov, Judith Fliegmann, Ravishankar Narayana, Massimo E. Maffei

**Affiliations:** 1 Department of Food Science, Aarhus University, Aarhus, Denmark; 2 ZMBP Center for Plant Molecular Biology, University of Tübingen, Tübingen, Germany; 3 Citrus Research and Education Center, University of Florida, Lake Alfred, FL USA; 4 Plant Physiology Unit, Department Life Sciences and Systems Biology, University of Turin, Turin, Italy

**Keywords:** Plant stress responses, Abiotic, Plant sciences

## Abstract

The geomagnetic field (GMF) is one of the environmental stimuli that plants experience continuously on Earth; however, the actions of the GMF on plants are poorly understood. Here, we carried out a time-course microarray experiment to identify genes that are differentially regulated by the GMF in shoot and roots. We also used qPCR to validate the activity of some genes selected from the microarray analysis in a dose-dependent magnetic field experiment. We found that the GMF regulated genes in both shoot and roots, suggesting that both organs can sense the GMF. However, 49% of the genes were regulated in a reverse direction in these organs, meaning that the resident signaling networks define the up- or downregulation of specific genes. The set of GMF-regulated genes strongly overlapped with various stress-responsive genes, implicating the involvement of one or more common signals, such as reactive oxygen species, in these responses. The biphasic dose response of GMF-responsive genes indicates a hormetic response of plants to the GMF. At present, no evidence exists to indicate any evolutionary advantage of plant adaptation to the GMF; however, plants can sense and respond to the GMF using the signaling networks involved in stress responses.

## Introduction

The geomagnetic field (GMF) is a natural component of our environment. It is fairly homogeneous and relatively weak. The strength of the GMF at the surface of the Earth ranges from less than 30 μT in an area that includes most of South America and South Africa (the so-called South Atlantic anomaly) to over 60 μT around the magnetic poles in northern Canada, the south of Australia, and in parts of Siberia^1^. Plants, which are known to sense different wavelengths of light, respond to gravity, and react to touch and to electrical signals, cannot avoid the presence of the GMF^2^. While phototropism, gravitropism, hydrotropism, and autostraightening have been thoroughly documented^3^, possible effects of the GMF on plant growth and development are still a matter of discussion. Nevertheless, a growing body of evidence indicates that plants do react to varying magnetic field (MF) fluxes at values both below and above the GMF^4,5^.

Three different mechanisms of magnetoperception have been described: (1) a mechanism involving radical pairs (i.e., magnetically sensitive chemical intermediates that are formed by photoexcitation of cryptochrome^6^), as has been demonstrated both in animals^7^ and in plants^8^; (2) the presence of MF sensory receptors present in cells containing ferromagnetic particles, as has been shown in magnetotactic bacteria^9^; and (3) the detection of minute electric fields by electroreceptors in the ampullae of Lorenzini in elasmobranch animals^10^. Plants show both light-dependent^8,11,12^ and light-independent^13–15^ magnetoreception, which may reflect a differential ability of plant organs to interact with light and the GMF. For instance, leaves are constantly exposed to both light and GMF, whereas roots perceive the GMF but only a low light fluence when close to the soil surface and no light when they grow deep in the ground.

In a previous study aimed at evaluating the effect of GMF reversal on plants, we found differential root/shoot responses in plant morphology and in the expression of some genes (e.g., *Cruciferin 3*, *Copper Transport Protein1* and *Redox Responsive Transcription Factor1*)^16^. This finding is in agreement with the current view that the magnetic reaction could change the ratio of redox states in the cryptochrome photocycle to alter the biological activity of cryptochrome^8,17^. We also found that the GMF impacted the flowering time by differentially regulating leaf and floral meristem genes^18^ and by altering the signaling by cryptochrome and phytochrome. In particular, blue light exposure led to a partial association between the GMF-induced changes in gene expression and an alteration in cryptochrome activation^14^. The GMF also affected plant mineral nutrition by influencing both root ion uptake and ion channel activity^19,20^. Similar results have been obtained in Arabidopsis and other plant species^4,8,12,21–24^. Of the three possible mechanisms of magnetoreception, only the radical pair mechanism of chemical magnetosensing adequately explains the alterations in the MF by the rates of redox reactions and subsequently altered concentrations of free radicals and ROS observed in plants, animals, and humans^8,16,25,26^. The theory underlying the radical pair mechanism predicts that magnetic fields similar in strength to the Earth’s geomagnetic field are too weak to trigger cellular biochemical reactions; however, these magnetic fields are able to interact with short-lived reaction intermediates that affect the reaction rates of biochemical reactions. Examples include photoreceptors (e.g., cryptochromes) and redox reactions that can be initiated by metabolic factors. This modulation of cryptochrome signaling and/or redox reactions can alter ROS synthesis in the cells^27^.

Despite numerous demonstrations of MF effects on plant growth and development, the mechanism of MF action is poorly understood. The aim of the present study was to obtain deeper insight into plant responses to MF variations by conducting a global expression time-course experiment separately in shoots and roots and by examining the effect of varying MF intensity on selected MF-responsive genes. We found differential gene expression responses to MF in the shoot and the roots. The biphasic dose response of GMF-responsive genes implicates a hormetic stress mechanism in the response of plants to the GMF.

## Results

### Time-course analysis reveals differential root and shoot gene expression patterns in plants exposed to reduced GMF

In order to assess the Arabidopsis responses to the GMF, we performed a transcriptomic time-course analysis by gene microarray of Arabidopsis seedlings vertically grown in Petri dishes and exposed to Near Null Magnetic Field (NNMF) conditions from 10 min to 96 h. Roots and shoots were sampled separately. Controls were represented by plants growing in the same conditions (i.e., temperature, gravity, atmospheric pressure and Photosynthetic Phlux Density—PPD) and in the presence of the GMF. For significant analysis, genes were filtered based on their correct P-values calculated from statistical analysis. Supplementary Table S1, gathers all information on gene expression changes, statistical analysis and fold change volcano plots for all time points assessed.

In general, almost all biological replicates analyzed were retained in the analysis and the genes satisfying a corrected P-value cut-off of 0.05 ranged from 21 to 30% out of the total gene number. A consistent percentage of these genes explained a fold change value > 2 at almost all time points, with the sole exception of plants exposed for 4 h to NNMF (Supplementary Table S2).

### The GMF differentially affects root and shoot genes

**Figure 1:**
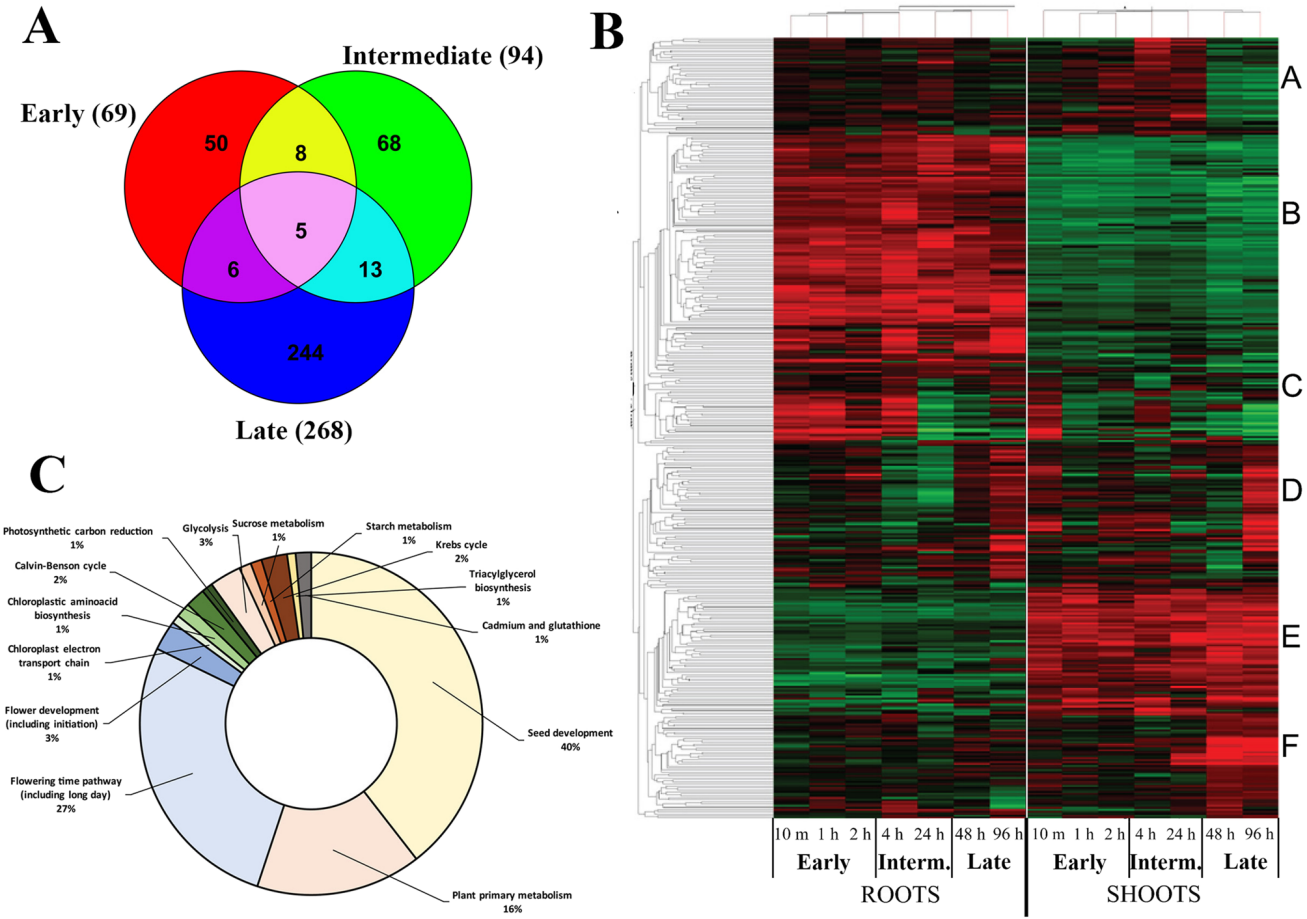
Transcriptomic time course analysis of *Arabidopsis thaliana* exposed to NNMF. (**A**) Venn diagram showing overlapping genes selected from the three time ranges. (**B**) hierarchical clustering of early, intermediate (Interm.) and late groups of regulated genes. Group A (composed of 49 differentially expressed genes, Supplementary Data Set S1) is upregulated in shoots at intermediate expositions and downregulated in the shoot at long term treatment. Group B (123 genes, Supplementary Data Set S2) is upregulated in the roots and downregulated in the shoots at all time points. Group C (31 genes, Supplementary Data Set S3) shows a biphasic induction in roots and upregulation in shoots only at the earliest time point. Group D (67 genes, Supplementary Data Set S4) is strongly upregulated at long time treatment (96 h). Group E (64 genes, Supplementary Data Set S5) is downregulated in the roots and upregulated in the shoots during all times, and Group F (47 genes, Supplementary Data Set S6) is upregulated in the shoots at long term treatment. (**C**) Pathway analysis of the genes selected for the hierarchical clustering.

We looked for genes which were differentially expressed at different time points in both shoots and roots. We selected 3 time ranges: early, which included samplings at time points at 10 min, 1 and 2 h; intermediate, which is represented by samplings at 4 and 24 h; and late, made by samplings at 48 and 96 h. For every time range, we identified genes which at least in 50% treatments showed significant differential expression at P < 0.05. The number of genes meeting the above mentioned requirements/conditions was 69 for early time, 94 for intermediate and 268 for late time, accounting for a total of 394 regulated genes. As shown in the Venn diagram of Fig. 1A, these genes partially overlapped. We calculated the hierarchical clustering (gene tree and conditional tree) using this set of genes and detected distinct patterns in the response of roots and shoots to NNMF. Based on the expression matrix we selected 6 groups with specific expression patterns (Groups A–F) (Fig. 1B).

With regard to Group A genes (upregulated in shoots at intermediate times and downregulated in shoots at longer times), the GO analysis of the 49 differentially expressed genes identified 47 genes with a known molecular function. The clustering of the GO analysis indicates that group A genes show an oxidoreductase activity (Supplementary Data Set S1). Genes associated to the cellular components are mainly expressed in the nucleus, cell wall, plasma membrane and chloroplasts, whereas the prevailing biological process are associated to genes responding to stress and transport (Supplementary Data Set S1). Nine genes with P value < 0.05 showed a fold change > 2 (Table 1) and the functional characterization of these genes indicates hydrolase activity (*At1g56680, At1g66270, At1g66280*), binding activity (*At1g74500, At5g66280, At2g25980*), transporter activity (*At5g50800*), seed storage (*At2g37870*) and a gene with unknown function (At2g41800). A significant late downregulation was found in shoots for *At1g56680, At1g66270, At1g66280, At1g74500*, *At2g25980, At2g41800* and *At5g50800*, whereas upregulation of *At2g37870, At5g50800* and *At5g66280* occurred in shoots at intermediate times (Table 1). Previous investigations showed that genes from this group are regulated by MF. Specifically, *At1g66280* (*BLUG22*) was modulated by treatments of 600 mT static magnetic field^24^. *At2g37870*, which codes for a bifunctional inhibitor/lipid-transfer protein/seed storage 2S albumin protein, and other gene of Group A *AT4G27560* (*UGT79B2*) glycosyltransferase were also found to be regulated by increasing the GMF intensity^24,28^. Interestingly, genes of this group are also responsive to stress. For example, two β -glucosidases (*At1g66270-BGLU21* and *At1g66280-BGLU22*), which belong to the subfamily 3 of GH family 1, are known to respond to salt stress, phosphate starvation and methyl jasmonate^29,30^.

**Table 1:** Group A, genes upregulated in shoots at intermediate times and downregulated in shoots at longer times. Selected genes showing a fold change > 2 and P value < 0.05 in at least one time point.

Gene model and description	Roots							Shoots						
	Early			Intermediate		Late		Early			Intermediate		Late	
	10 m	1 h	2 h	4 h	24 h	48 h	96 h	10 m	1 h	2 h	4 h	24 h	48 h	96 h
At1g56680: Chitinase family protein	1.035	0.927	0.946	1.262	0.873	0.924	1.179	0.859	0.857	1.107	0.953	1.232	0.924	**0.496**
At1g66270 BGLU21 encodes a beta-glucosidase	0.986	1.194	0.884	1.213	0.889	1.086	0.727	0.848	1.032	1.639	1.153	1.426	1.086	**0.526**
At1g66280 BGLU22 Glycosyl hydrolase superfamily protein	1.09	1.135	0.939	1.113	1.019	1.135	0.781	0.927	1.227	1.393	1.356	1.392	1.135	**0.570**
At1g74500 ATBS1, ACTIVATION-TAGGED BRI1 (BRASSINOSTEROID-INSENSITIVE 1)-SUPPRESSOR 1	1.018	1.014	0.988	1.259	0.94	1.055	1.016	0.796	0.898	1.222	1.301	1.278	1.055	**0.516**
At2g25980 Mannose-binding lectin superfamily protein	0.984	1.045	0.974	1.16	0.938	1.029	0.932	0.914	1.001	1.445	1.635	1.024	1.029	**0.527**
At2g37870 Bifunctional inhibitor/lipid-transfer protein/seed storage 2S albumin superfamily protein;	0.96	1.145	0.995	1.207	0.938	1.293	1.092	0.79	0.958	0.907	**1.978**	1.081	1.293	1.123
At2g41800 TEB, Encodes a DUF642 cell wall protein that is highly induced during the M/G1 phases of the cell cycle and is involved in hypocotyl cell elongation	0.992	1.021	0.914	1.339	0.869	1.033	0.809	0.886	1.07	1.107	1.256	1.085	1.033	**0.543**
At5g50800 RPG2, RUPTURED POLLEN GRAIN 2, Encodes a member of the SWEET sucrose efflux transporter family protein	1.135	1.044	1.229	1.016	1.06	0.913	1.613	1.247	1.26	1.375	1.216	**3.061**	0.913	**0.460**
At5g66280 GMD 1, GDP-D-mannose 4,6-dehydratase	1.088	1.235	0.982	1.242	1.01	1.196	0.722	0.739	0.681	0.801	**2.862**	1.812	1.196	0.688

Boldfaced numbers indicate fold changes with a P value < 0.05.

Group B consists of 123 genes characterized by a clear differential expression between roots and shoots at all time points, with upregulated root genes and downregulated shoot genes. The total GO analysis revealed 109 genes with a known function. The clustering of GO analysis indicates a zinc ion biding activity (Supplementary Data Set S2). Chloroplasts and, to a minor extent, mitochondria and the nucleus were the cellular components associated to the group B genes, whereas the biological process associated with these genes were mainly transcription, response to abiotic and biotic stimulus and transport (Supplementary Data Set S2). Fifteen genes were found significantly expressed with a fold change > 2 and P < 0.05. Some of these genes were upregulated in the roots at early, intermediate and late times and two genes were downregulated in shoots at late times (Table 2). The functional characterization of these genes showed nucleotide, DNA and nucleic acid binding activity (*At1g33890*, *At2g21650*, *At5g27810*), non-DNA binding (*At3g47710*), transferase (*At1g10880*) and kinase (*At1g58643*) activity, hydrolase activity (*At1g06990*), transporter activity (*At5g09720, At5g52680)* while many other genes were of an unknown function (*At1g04670*, *At3g58210*, *At3g61340*, *At4g18335*, *At5g16330, At5g10130*) (Table 2). In a previos investigation, the sensitivity of several genes of Group B, specifically *At1g18410*, *At4g14370* and *At5g07780* in response to MF variation was shown when plants were exposed to MF intensities higher than the GMF^24^. Several genes of this group were also associated to both biotic and abiotic stress. The root induced genes of group B include a highly upregulated hypothetical protein coded by the gene, *At4g18335*, that shows homology (E-value 0.38) with a *SERINE CARBOXYPEPTIDASE-LIKE 51* (*At2g27920-SCPL51*) which is involved in regulation of defence responses against biotic and oxidative stress^31^.

**Table 2:** Group B, genes characterized by a clear differential expression between roots and shoots at all time points. Selected genes showing a fold change > 2 and P value < 0.05 in at least one time point.

Gene model and description	Roots							Shoots						
	Early			Intermediate		Late		Early			Intermediate		Late	
	10 min	1 h	2 h	4 h	24 h	48 h	96 h	10 min	1 h	2 h	4 h	24 h	48 h	96 h
At1g04670 hypothetical protein	**2.05**	**2.804**	**2.299**	**2.045**	**2.117**	**3.086**	**3.093**	0.700	0.768	0.731	0.804	0.769	0.628	0.650
At1g06990 GDSL-motif esterase/acyltransferase/lipase	1.774	**2.774**	1.842	**3.058**	1.24	1.536	**2.532**	0.928	0.76	0.723	1.044	0.768	0.609	0.642
At1g10880 Putative role in response to salt stress	**2.226**	**2.156**	**2.179**	**2.359**	1.933	1.894	**3.338**	0.981	0.86	0.829	0.914	0.701	0.687	0.729
At1g33890 IAN3, IMMUNE ASSOCIATED NUCLEOTIDE BINDING 3	**2.616**	1.911	1.537	1.655	**2.306**	1.392	**2.037**	0.669	0.917	0.682	0.788	0.76	0.511	0.631
At1g58643 Inositol-pentakisphosphate 2-kinase family protein	**2.054**	1.113	1.219	1.532	**2.031**	**2.02**	**2.572**	0.780	0.843	0.778	0.836	0.987	0.666	0.850
At2g21650 ATRL2, MATERNAL EFFECT EMBRYO ARREST 3	1.46	1.567	1.92	1.416	1.869	1.082	1.24	0.562	1.238	0.953	1.013	0.946	0.589	**0.476**
At3g47710 BASIC HELIX-LOOP-HELIX PROTEIN 161, BHLH161	1.222	1.040	1.153	1.660	1.515	1.430	1.182	0.625	0.823	0.535	0.670	0.972	1.040	**0.354**
At3g58210 TRAF-like family protein	**2.769**	**2.026**	1.85	**2.497**	1.478	**2.392**	**2.887**	0.786	0.812	0.78	0.862	0.827	0.695	0.693
At3g61340 F-box and associated interaction domains-containing protein	**2.425**	1.557	1.517	**2.095**	1.496	1.71	**2.552**	0.56	0.604	0.586	0.639	0.526	0.509	0.543
At4g18335 hypothetical protein	**2.154**	**2.002**	1.533	**2.741**	**2.411**	**2.377**	**3.568**	0.774	0.711	0.652	0.714	0.68	0.621	0.582
At5g09720 Magnesium transporter CorA-like family protein	**2.676**	1.632	1.298	1.768	1.628	1.551	**2.076**	0.736	0.79	0.736	0.81	0.778	0.684	0.657
At5g10130 Pollen Ole e 1 allergen and extensin family protein	1.09	1.135	1.078	1.39	0.911	1.364	1.085	0.851	0.68	0.859	0.624	1.076	0.682	**0.560**
At5g16330 NC domain-containing protein-like protein	**2.545**	**2.337**	**2.049**	**2.686**	1.34	1.489	**2.226**	0.793	0.793	0.756	0.834	0.799	0.635	0.668
At5g27810 MADS-box transcription factor family protein	**2.045**	**2.907**	**2.599**	**2.481**	**2.46**	**2.437**	1.807	0.734	0.772	0.738	0.806	0.776	0.752	0.778
At5g52680 Copper transport protein family	1.499	1.162	1.583	1.167	**2.306**	**2.261**	**3.188**	0.935	0.663	0.731	0.662	0.903	0.858	0.837

Boldfaced numbers indicate fold changes with a P value < 0.05.

Among the 31 genes of Group C, which are upregulated in the root at early and late time treatment and at very early time treatment in the shoot, 28 had a known function and the GO analysis revealed that the main cellular component involved were mitochondria, chloroplasts (and plastids) and the nucleus, whereas the genes of this group were associated to biological processes such as developmental processes, protein metabolism, responses to stress (including abiotic and biotic) and transport (Supplementary Data Set S3). The general GO analysis of the 28 genes indicates a major involvement in seed development (Supplementary Data Set S3). A significant upregulation at early times in roots and shoots was found for several genes which also showed a intermediate time downregulation in roots and a late downregulation in shoots (Table 3). The molecular function of these genes was catalytic and binding activity (*At4g26740*), nutrient reservoir activity (*At2g28490*, *At4g25140, At4g28520, At5g54740)* and acquisition of desiccation tolerance (*At2g41260*) (Table 3).

**Table 3:** Group C, genes upregulated in the root at early and late time treatment and at very early time treatment in the shoot. Selected genes showing a fold change > 2 and P value < 0.05 in at least one time point.

Gene model and description	Roots							Shoots						
	Early			Intermediate		Late		Early			Intermediate		Late	
	10 min	1 h	2 h	4 h	24 h	48 h	96 h	10 min	1 h	2 h	4 h	24 h	48 h	96 h
At2g28490 RmlC-like cupins superfamily protein	**2.025**	**2.257**	1.156	**0.421**	0.708	**2.099**	0.710	1.696	0.623	0.740	1.235	0.737	1.319	**0.321**
At2g41260 Late-embryogenesis-abundant gene. M17	**2.180**	1.961	1.960	0.501	0.568	0.784	**0.489**	**2.306**	0.611	0.746	0.788	0.877	1.462	0.910
At4g25140 OLEOSIN 1. OLEO1 Encodes a protein found in oil bodies, involved in seed lipid accumulation	**2.069**	**2.729**	1.687	**0.416**	0.725	**2.127**	0.835	**2.206**	0.600	0.836	1.555	0.852	1.352	**0.358**
At4g26740 Peroxygenase 1, ATPXG1, CALEOSIN1, CLO1, caleosin, a 27-kDa protein found within seed lipid bodies	1.881	**2.275**	1.802	**0.483**	0.659	0.846	1.091	1.650	0.811	1.140	1.438	0.676	1.473	**0.478**
At4g28520 CRUCIFERIN 3. CRU3	**2.829**	**5.167**	**4.905**	**0.274**	**0.434**	1.959	0.542	**3.205**	0.613	0.834	0.544	0.906	0.683	**0.271**
At5g54740 SEED STORAGE ALBUMIN 5, SESA5	1.942	**2.466**	**3.285**	**0.201**	**0.441**	**2.077**	0.704	**3.086**	1.023	1.466	0.697	**0.309**	**0.35**	**0.487**

Boldfaced numbers indicate fold changes with a P value < 0.05.

Out of the 67 genes of Group D, which are characterized by a strong upregulation at late times, 57 had a known function. The main cellular components involved were chloroplasts, mitochondria and the plasma membrane, whereas the major biological processes were response to stress (both biotic and abiotic), transcription and signal transduction (Supplementary Data Set S4). The general GO analysis of the 57 genes with a known functions points to both DNA-dependent regulation of transcription and to defence response (including endogenous response to chitin) (Supplementary Data Set S4). Five genes showed a significant upregulation al late times in both roots and shoots (Table 4) and were involved in DNA binding (*At3g44350*, *At4g34410*), signal transduction (*At3g23120*), copper transport (*At5g52760*) and unknown function (*At5g22520*). In the group D, *At4g34410* encodes a member of the ERFs (ethylene response factor; *ERF109*) involved in the adaptation to biotic or abiotic stresses^32^. *ERF109* (also known as *RRTF*) responds to ethylene and jasmonic acid in order to regulate redox homeostasis^32^. The gene shows tissue-specific responsiveness to various abiotic stress treatments including a response to salt stress in roots^33^. Other salt stress-related gene was *At5g47220* (*ERF2*), a gene that is overexpressed by the presence of heavy metals (Cd, Cu and Al)^24,34^.

**Table 4:** Group D, genes characterized by a strong upregulation at late times. Selected genes showing a fold change > 2 and P value < 0.05 in at least one time point.

Gene model and description	Roots							Shoots						
	Early			Intermediate		Late		Early			Intermediate		Late	
	10 min	1 h	2 h	4 h	24 h	48 h	96 h	10 min	1 h	2 h	4 h	24 h	48 h	96 h
At3g23120 RECEPTOR LIKE PROTEIN 38. RLP38	1.307	1.313	1.162	**2.304**	1.191	1.248	**5.079**	0.840	0.857	0.781	0.848	1.253	0.777	**2.958**
At3g44350 NAC DOMAIN CONTAINING PROTEIN 61. ANAC061	0.525	0.823	0.853	0.934	0.927	0.773	**3.600**	0.783	1.338	1.268	1.415	1.103	0.998	**3.116**
At4g34410 ETHYLENE RESPONSE FACTOR 109. ERF109. REDOX RESPONSIVE TRANSCRIPTION FACTOR 1, RRTF1	0.875	1.526	**3.874**	0.523	1.579	0.776	**2.517**	1.538	**0.464**	0.878	**0.448**	1.892	0.51	**11.090**
At5g22520 hypothetical protein	0.836	0.680	1.063	1.183	1.001	1.686	**4.066**	1.139	0.716	0.747	0.820	0.695	0.971	**2.488**
At5g52760 Copper transport protein family	0.773	0.899	1.042	1.035	1.458	0.952	**6.372**	1.213	1.205	0.928	0.934	0.946	1.833	**5.993**

Boldfaced numbers indicate fold changes with a P value < 0.05.

The group E was made by 64 genes which were in general downregulated in the root and upregulated in the shoot at all times. Fifty-eight genes had a known function and the main cellular components involved were nucleus, cell wall, chloroplasts, mitochondria and the plasma membrane, whereas transcription, response to stress (both biotic and abiotic), protein metabolism, developmental processes and transport were the main biological processes involved (Supplementary Data Set S5). The general GO analysis points to a DNA-dependent regulation of transcription (Supplementary Data Set S5). A significant regulation was found for 28 genes, mostly upregulated in the shoots at different timings (Table 5). Specifically, 5 genes (*At1g19510*, *At2g42830*, *At5g18000*, *At5g46830*, *At5g60130*) were involved in nucleic acid and DNA binding; 6 in protein and other binding (*At1g16410*, *At1g17610, At3g59510*, *At3g60890*, *At5g50790*, *At5g55450*); 4 showed hydrolase activity (*At1g56710*, *At2g46880*, *At3g57240*, *At5g51530*); 2 kinase activity (*At1g29720*, *At4g23230*), a gene with transferase activity (*At3g12470*), a gene with transmembrane transporter activity (*At5g17830*), a transposable element gene (*At1g41650*), 3 defense and resistance genes (*At3g50450, At5g40155, At5g44420*), a gene involved in iron-sulfur cluster assembly (*At4g32990*), a gene involved in seed storage (*At5g62080*) and 3 genes on unknown function (*At1g78030*, *At1g79770*, *At3g59230*) (Table 5). In the group E, the shoot upregulation of the transposable element *At1g41650* is of particular interest. Transposable elements mobilize in response to stress elicitors, including biotic and abiotic cues, and can also confer stress inducibility modulated through their alternative methylation and demethylation in the gene promoter regions^35^. Several genes belonging to the group E have been found to be regulated by alterations of the GMF in other studies. For instance, *At1g51840* (*SIF1*), a classic LRR-RLK protein that responds to abiotic stress and is downregulated in the roots upon drought treatment^36^ was oppositely regulated by increasing GMF intensity^24^. LRR-RLK have been found to positively regulate plant biotic resistance and negatively regulate plant abiotic tolerance^36^. A strong shoot late regulation was found for *At1g01980* (*OGOX4*) that encodes an oligogalacturonide oxidase that inactivates the elicitor-active oligogalacturonides^37^; whereas *At5g55420*, a pseudogene that encodes a protease inhibitor/seed storage/LTP family protein shares a good homology (E value 6e−106) with *At5g55450* (*ATLTP4.4*), a bifunctional inhibitor/lipid-transfer protein/seed storage 2S albumin superfamily protein which is involved in maintaining the redox state^38^ and is involved in the systemic acquired resistance (SAR) pathway^39^.

**Table 5:** Group E, genes characterized by a general downregulation in the root and upregulation in the shoot at all times. Selected genes showing a fold change > 2 and P value < 0.05 in at least one time point.

Gene model and description	Roots							Shoots						
	Early			Intermediate		Late		Early			Intermediate		Late	
	10 min	1 h	2 h	4 h	24 h	48 h	96 h	10 min	1 h	2 h	4 h	24 h	48 h	96 h
At1g16410 CYP79F1, the mRNA is cell-to-cell mobile	0.923	0.854	0.772	0.792	1.136	0.925	0.870	1.354	**2.253**	1.884	1.857	**2.733**	**2.387**	**2.177**
At1g17610 CHS1, CHILLING SENSITIVE 1, TN-type protein that controls temperature-dependent growth and defense responses	0.839	0.798	0.818	0.678	0.918	0.882	1.157	1.644	1.277	1.108	1.192	1.42	**2.017**	**2.112**
At1g19510 RAD-LIKE 5 (RADIALIS-LIKE SANT/MYB 4) transcription factor	0.852	0.457	0.919	0.996	0.821	0.726	0.754	1.651	1.582	1.120	1.269	1.683	1.567	1.280
At1g29720 Leucine-rich repeat transmembrane protein kinase	0.608	0.675	1.158	0.703	0.954	0.733	0.881	1.955	**2.726**	**2.159**	1.259	1.081	1.134	1.209
At1g41650 Transposable element gene	0.551	0.638	0.601	1.738	1.064	**0.476**	1.123	0.844	**2.852**	**2.388**	**2.082**	0.964	1.899	**2.353**
At1g56710 PGL1, POLYGALACTURONASE LIKE 1	0.777	0.687	0.666	0.584	0.777	0.691	0.663	**2.132**	**2.188**	1.646	1.645	**2.022**	1.804	1.700
At1g78030 hypothetical protein	0.855	0.827	0.790	0.813	0.890	0.898	1.005	1.487	**2.101**	**2.228**	1.325	1.369	1.843	**2.048**
At1g79770 CASP-like protein (DUF1677)	0.627	0.555	**0.347**	0.59	0.575	0.585	**0.499**	1.378	1.756	1.38	1.554	1.594	1.414	1.704
At2g42830 AGL5, AGAMOUS-LIKE 5, MADS box protein involved in fruit development	0.546	0.572	**0.484**	0.798	0.812	0.601	0.749	1.54	1.174	1.258	1.115	1.525	**2.058**	1.884
At2g46880 PAP14, PURPLE ACID PHOSPHATASE	1.203	1.453	0.812	1.226	0.921	0.875	0.918	1.291	1.894	1.604	**5.098**	1.691	**3.309**	**2.978**
At3g12470 Polynucleotidyl transferase, ribonuclease H-like superfamily protein	0.761	0.817	0.658	0.830	0.723	0.700	0.734	1.926	1.788	1.419	1.891	**2.637**	1.922	**2.401**
At3g50450 HR1, HOMOLOG OF RPW8 1	0.852	0.665	0.873	0.822	0.889	0.901	0.872	1.712	**2.594**	1.843	1.529	1.270	**2.636**	**4.111**
At3g57240 BG3, BETA-1,3-GLUCANASE 3	0.713	0.677	0.689	0.773	0.736	0.736	1.124	1.444	1.639	**2.130**	1.456	1.954	1.378	**2.104**
At3g59230 RNI-like superfamily protein	0.821	0.810	0.782	0.805	0.883	0.841	0.886	1.886	1.54	1.868	1.325	**2.923**	**2.457**	**3.004**
At3g59510 Leucine-rich repeat (LRR) family protein	0.805	0.807	0.773	0.859	0.869	0.821	0.883	1.816	**2.218**	1.478	**2.408**	1.967	1.613	1.721
At3g60890 ZPR2, LITTLE ZIPPER 2, binding protein	0.808	0.901	0.779	0.816	0.919	0.834	0.860	1.339	1.591	1.938	1.481	**2.616**	**2.691**	**2.369**
At4g23230 CRK15, CYSTEINE-RICH RLK (RECEPTOR-LIKE PROTEIN KINASE) 15	0.777	0.797	0.764	0.782	0.770	0.981	0.859	1.947	1.376	**2.617**	1.949	**2.073**	**2.160**	**2.544**
At4g32990 Transducin/WD40 repeat-like superfamily protein	0.804	0.628	0.753	0.624	0.690	0.78	0.715	**2.117**	1.415	1.920	1.061	1.469	**2.453**	1.714
At5g17830 Plasma-membrane choline transporter family protein	0.826	0.859	0.702	0.716	0.758	0.702	0.705	1.804	1.489	1.506	1.327	**2.102**	**2.011**	1.409
At5g18000 VDD, VERDANDI, a putative transcription factor belonging to the reproductive meristem (REM) family	0.919	0.684	**0.472**	0.928	0.702	0.545	0.743	1.724	1.238	1.089	1.185	0.824	**2.403**	1.727
At5g40155 Encodes a defensin-like (DEFL) family protein	0.708	0.629	0.621	0.519	0.735	0.614	0.759	1.866	**2.170**	1.929	1.879	1.722	**2.485**	**2.721**
At5g44420 PDF1.2, PLANT DEFENSIN 1.2 Encodes an ethylene- and jasmonate-responsive plant defensin	0.839	0.675	0.594	0.783	0.735	0.779	1.197	1.452	1.069	1.145	1.208	**2.353**	**2.531**	**2.232**
At5g46830 NIG1, Calcium-binding transcription factor involved in salt stress signaling	0.720	1.091	0.902	0.600	0.636	1.012	0.938	1.392	1.271	1.217	**2.779**	**2.054**	0.787	0.872
At5g50790 SWEET10, Encodes a member of the SWEET sucrose efflux transporter family proteins	0.747	0.773	0.817	0.904	0.915	0.877	**0.454**	1.651	**2.585**	**2.245**	1.382	**3.324**	**3.641**	**2.454**
At5g51530 Ubiquitin carboxyl-terminal hydrolase-related protein	0.756	0.661	0.771	0.654	0.811	1.001	0.766	2.000	1.225	1.319	0.903	1.164	**2.571**	**2.011**
At5g55450 ATLTP4.4 Bifunctional inhibitor/lipid-transfer protein/seed storage 2S albumin superfamily protein	0.843	0.777	0.774	0.903	0.859	0.813	0.867	1.652	1.839	1.498	1.876	1.795	**2.306**	**2.022**
At5g60130 AP2/B3-like transcriptional factor family protein	0.698	0.647	0.719	0.653	0.716	0.773	0.670	1.447	1.404	1.390	1.881	1.526	**2.033**	**2.223**
At5g62080 Bifunctional inhibitor/lipid-transfer protein/seed storage 2S albumin superfamily protein	0.626	0.621	0.603	0.611	0.679	0.647	0.667	**2.559**	**2.169**	1.757	**2.310**	**2.491**	1.885	**3.024**

Boldfaced numbers indicate fold changes with a P value < 0.05.

The last Group F consisted of 47 genes which were characterized by a late upregulation in the shoots. The functional characterization was obtained for 43 genes and the major cellular components involved were chloroplasts, nucleus, cell wall and the plasma membrane, whereas the main biological processes involved were response to stress (both biotic and abiotic), developmental processes, protein metabolism, transcription and cell organization and biogenesis (Supplementary Data Set S6). Fourteen genes showed a significant regulation in the shoots, especially at late times (Table 6) and 7 were involved in DNA, nucleotide and protein binding (*At1g01980*, *At2g33270*, *At3g04510*, *At3g18550*, *At5g23260*, *At5g60140, At5g61080)*, a gene involved in seed storage (*At5g55420*), a gene with heme binding, peroxidase activity (*At5g58400*) and 5 genes on unknown function (*At1g05550*, *At1g67865*, *At3g49230*, *At4g19430*, *At5g43240*).

**Table 6:** Group F, genes characterized by a late upregulation in the shoots. Selected genes showing a fold change > 2 and P value < 0.05 in at least one time point.

Gene code	Roots							Shoots						
	Early			Intermediate		Late		Early			Intermediate		Late	
	10 min	1 h	2 h	4 h	24 h	48	96 h	10 min	1 h	2 h	4 h	24 h	48	96 h
At1g01980 OLIGOGALACTURONIDE OXIDASE 4. OGOX4	− 1.14	− 1.19	− 1.20	− 1.19	1.80	− 1.14	1.00	1.50	− 1.01	1.22	1.26	**3.33**	**4.13**	**3.28**
At1g05550 DUF295 ORGANELLAR A 2	− 1.08	− 1.07	− 1.22	1.09	1.31	1.39	1.51	− 1.11	− 1.18	− 1.11	1.20	1.26	**2.83**	**2.92**
At1g67865 hypothetical protein	− 1.04	1.01	1.03	− 1.06	1.03	− 1.02	1.04	− 1.08	1.41	− 1.13	− 1.04	**2.60**	**4.35**	**2.24**
At2g33270 ACHT3, ATYPICAL CYS HIS RICH THIOREDOXIN 3	− 1.54	1.25	− 1.02	− 1.08	1.08	− 1.05	− 1.02	1.01	1.05	1.29	1.31	1.19	**2.23**	**2.89**
At3g04510 LIGHT SENSITIVE HYPOCOTYLS 2. LSH2	− 1.24	1.07	− 1.28	− 1.24	1.08	− 1.04	1.35	1.07	1.42	− 1.16	− 1.02	1.83	**2.48**	**2.21**
At3g18550 TCP transcription factor, closely related to teosinte branched1, arrests axillary bud development and prevents axillary bud outgrowth	− 1.10	− 1.05	1.10	1.04	1.44	− 1.01	− 1.10	− 1.05	1.01	1.46	− 1.11	1.31	**2.84**	**3.06**
At3g49230 Unknown transmembrane protein	− 1.14	− 1.27	− 1.15	− 1.11	− 1.02	1.08	1.12	1.24	− 1.28	− 1.10	1.22	1.23	**3.04**	**2.94**
At4g19430 hypothetical protein	− 1.09	− 1.10	− 1.14	− 1.17	1.09	− 1.03	− 1.05	− 1.14	1.04	1.04	1.00	**2.21**	**7.01**	**4.21**
At5g23260 Encodes a MADS box protein	− 1.08	− 1.11	− 1.09	− 1.06	1.20	− 1.02	− 1.19	1.22	− 1.09	− 1.08	1.31	1.17	**3.26**	**3.70**
At5g43240 hypothetical protein	− 1.07	1.09	− 1.14	1.09	− 1.01	− 1.06	1.07	− 1.14	− 1.12	− 1.14	− 1.07	**2.03**	**3.27**	1.98
At5g55420 Encodes a Protease inhibitor/seed storage/LTP family protein	1.05	− 1.09	− 1.12	− 1.08	1.00	− 1.05	1.13	− 1.13	1.07	− 1.04	1.07	1.21	**3.56**	**2.03**
At5g58400 Peroxidase superfamily protein	− 1.22	1.01	− 1.36	− 1.43	− 1.01	− 1.03	1.00	1.11	1.02	− 1.17	1.35	1.68	1.83	**2.19**
At5g60140 AP2/B3-like transcriptional factor family protein	− 1.22	− 1.09	1.23	1.16	1.24	− 1.07	− 1.15	1.13	1.00	− 1.04	− 1.12	1.41	**2.89**	**3.62**
At5g61080 Ribonuclease H-like protein, nucleic acid binding	− 1.36	− 1.06	− 1.11	− 1.08	1.18	1.10	1.01	1.06	1.20	1.58	1.16	− 1.04	**2.37**	**3.20**

Boldfaced numbers indicate fold changes with a P value < 0.05.

As expected, the pathway analysis calculated on the pooled groups reveals the prevalence of genes involved in seed development, followed by plant primary metabolism and flowering time and development pathways (Fig. 1C and Supplementary Data Set S7).

We also focused our attention on early regulated genes to underline the mechanisms triggering the first responses to MF variations. Analysis of promoter sequences using the Plant RegMap^40^ and TF2Network tools^41^ showed over-representation of NAC transcription factors in the promoter sequence of differentially regulated genes after a 10 min exposure to NNMF (Supplementary Data Set S8).

### Arabidopsis roots and shoots selected genes respond to different magnetic field strengths

By observing the gene clustering of Fig. 1B, we focused our attention on some selected genes in order to assess their response to varying MF strengths. By varying the voltage applied to the triaxial Helmholtz coils we exposed Arabidopsis plants for 96 h to different static MF strengths (which were measured by the triaxial fluxgate sensor). The different voltages applied to the three couples of Helmholtz coils generated B values of 240 nT, 11 μT, 18 μT, 21 μT, 34 μT, 41 μT, 50 μT and 60 μT, with 41 μT representing the GMF B value of controls. qRT-PCR was performed for each MF strength on selected genes and the data were plotted as fold change. In order to emphasize the visualization of data, values < 1 were plotted as − 1/value, in order to obtain negative fold change data (indicating downregulation).

In general, all selected genes showed a differential expression when exposed to varying MF strengths, indicating the ability of the plant to perceive and respond to different MFs. Moreover, a differential gene expression was also observed between roots and shoots, confirming and validating the data obtained during the microarray time course analysis.

**Figure 2:**
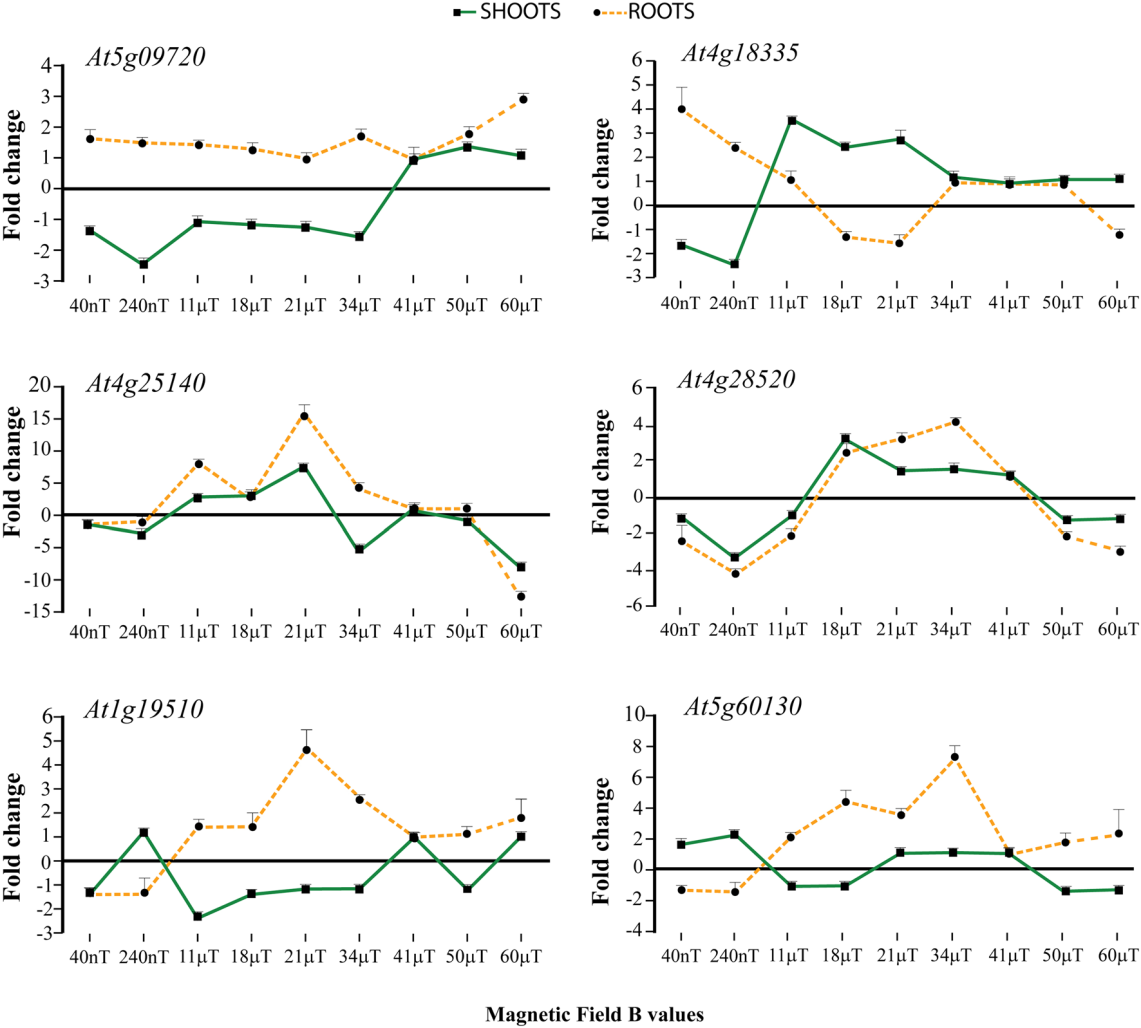
Differential gene expression of roots and shoots selected genes in response to varying MF intensity. The data are expressed as fold change in relation to controls (measured at 41 μT). In order to emphasize the visualization of data, fold change values below one were plotted as − 1/value, in order to obtain negative fold change values (indicating downregulation). Group B genes: *At5g09720* and *At4g18335*; Group C genes: *At4g25140* and *At4g28520*; Group E genes: *At1g19510* and *At5g60130*. Metric bars indicate standard deviation.

We analysed two genes belonging to the cluster B: *At5g09720*, a magnesium transporter CorA-like family protein, and *At4g18335*, that codifies a hypothetical protein of unknown function. *At5g09720* expression in shoots was always downregulated from 40 nT to 34 μT and showed an opposite trend when compared to the root gene expression. At 41 μT and up to 60 μT shoot and roots *At5g09720* showed an upregulated gene expression, which was particularly evident in roots (Fig. 2). A remarkable difference in root and shoot gene regulation was found for *At4g18335* from 40nT to 21 μT and the expression in the two organs was equal (and not regulated) from 34 μT to 60 μT. A strong upregulation in the roots and a consistent downregulation in shoots were confirmed at NNMF intensities and the trend was reversed between 18 μT and 21 μT (Fig. 2).

The two selected genes of the cluster C were: *At4g25140* (OLEO1), that encodes a protein found in oil bodies, involved in seed lipid accumulation and *At4g28520* (CRU3), which encodes a 12S seed storage protein that is tyrosine-phosphorylated and its phosphorylation state is modulated in response to ABA in *A. thaliana* seeds. The expression in roots and shoots of both genes followed the same pattern of variation with increasing MF intensity, with MF strength between 40nT and 11 μT and above 50 μT inducing downregulation, and MF strength between 18 and 41 μT inducing upregulation of the gene (Fig. 2).

Finally, the two selected genes of cluster E were *At1g19510*, a RAD-LIKE 5 (RADIALIS-LIKE SANT/MYB 4) transcription factor and *At5g60130*, an AP2/B3-like transcriptional factor family protein. The expression of both genes showed an opposite trend in roots and shoots with increasing MF strengths. The differential gene expression was particularly evident in the MF range between 11 and 41 μT (Fig. 2).

## Discussion

The results of this work provide evidence that (i) roots and shoot have different gene expression responses to the GMF, as almost 50% of the regulated genes were triggered in a reverse manner; (ii) the effects of the GMF are related to activation of stress responsive genes; and (iii) the majority of identified GMF-responsive genes show biphasic dose-dependent expression, indicating a hormetic response of plants to MFs.

### Differential response of roots and shoots to magnetic field

In this study, we found that roots and shoots have different responses to MFs. This evidence is based on global analysis of differentially expressed genes in roots and shoots, where 49% of all responsive genes were regulated in a reverse direction. Moreover, qRT-PCR data for selected GMF-induced genes confirmed this differential expression response over a range of MF fluxes for most of these genes.

That roots and shoots can respond differently to the same stimuli might be related to the different physiological functions of these organs and to the different roles of these organs in adaptation to their environments. Indeed, previous AtGenExpress global stress response studies have shown that the same genes in shoots and roots have different kinetics^42^. Interestingly, the reverse responses of roots and shoots to different stresses was found at both the gene expression level and at the metabolomics and functional levels. For example, drought stress activates the uptake of water and nutrients in roots, whereas it decreases growth and metabolism in the shoot to lower the concentrations of sugars, amino acids, nucleosides, and mineral nutrients^43^. Simultaneous application of drought and warm temperature stress also cause differential regulation of primary and secondary metabolites in roots and shoots of perennial grasses. These stresses increased the concentrations of terpenes, catechins and indole acetic acid in shoots, whereas the roots showed elevations in the levels of amino acids, quinic acid, nitrogenous bases, choline, and glycine betaine^44^. Roots and shoots also show very different responses to biotic stresses such as herbivory, where root herbivores induce systemic defensive responses in plants and shoot herbivore induced mostly local defensive response without triggering a systemic response^45^. Light signalling as well leads to differential responses in shoots and roots^46–48^. For example, photoreceptor-dependent effects on plant elongation growth are opposite in roots and shoots and probably reflect differential responses to phytohormones in the respective tissues and organs^49,50^.

These contrasting responses of roots and shoots are observed upon exposure to stress factors as well as to other stimuli that have an informative nature, such as gravity and light. The positive gravitropism and negative phototropism of roots and the reverse actions in shoots have been well characterized^51^. In both these tropic responses, the plant hormone auxin plays a key role. Roots and shoots also differ in their dose responses to auxin, as auxin concentrations that stimulate hypocotyl growth inhibit root growth^52^. This implicates auxin in the differential responses of shoots and roots to these stimuli.

The differential regulation of genes in roots and shoots might also be related to the different roles of plastids in the roots and shoots. Indeed, GO analysis has shown that genes related to chloroplast functions were overrepresented among the genes regulated by the GMF, indicating that different functions of plastids in roots and shoots can contribute to the differential responses of roots and shoots to the GMF. These observations support recent findings that chloroplasts are one of the main targets of MF effects in Arabidopsis^24^. One important consideration is that the function of chloroplasts is related to the cellular redox status^53^, which can induce ROS imbalances and modulate the expression of genes induced by different stresses. Indeed, GO analysis has shown that variations in MFs affect the regulation of stress-responsive genes. Moreover, early gene modulations by MFs are associated with redox responses, implying that rapid rates of redox reactions, triggered by a MF, alter the metabolism of free radicals and ROS.

### Relation of magnetic field variations with regulation of stress-responsive genes

The general biological responses identified by gene ontology analyses indicate a strong association among the responses to stress (particularly salt stress and both biotic and abiotic stress). This association suggests that the GMF might affect ROS level, which are modulated by any type of stress, and that the GMF can interact with other stress factors to modulate plant responses. Indeed, the GMF affects the ability of plants to respond to several stress conditions, including UV-B^54^, salinity^55^, water stress^56^ and oxidative stress ^4,8^, indicating a potential cross-talk between the GMF and ROS.

Previous investigations have shown that the GMF affects the redox level in plant cells^16,57^. This redox modulation might reflect a direct effect of MFs on the redox ability of compounds, such as glucose and hydrogen peroxide, which can interfere with the ROS balance in the cells. ROS are considered to serve as common messengers in plant responses to different stresses, and modulation of ROS levels is thought to affect the expression of stress-responsive genes^42,58^. In this context, the over-representation of stress-responsive genes in the set of GMF-responsive genes is not surprising, assuming that the same messengers, such as ROS, play a key role in the responses to MFs and to different stresses. In agreement with this idea, different stresses, such as cold, drought, and osmotic stresses, are now known to share several response genes, implying that plants cope with these stresses by utilizing overlapping signaling cascades to integrate similar kinds of information^42,59^.

A direct effect of MFs on ROS formation has been shown for different organisms, tissues, and in vitro cultures; however, other studies have also reported decreases in ROS levels or no effect of MFs^60^. Multiple factors could give rise to these discrepancies, so further investigation of these phenomena is required.

The near-null MF affects the redox level in the plants; therefore, it can interfere with plant physiology specifically by decreasing the reduction of Fe^61^. This reduction is crucial for Fe uptake in plants such as Arabidopsis, which uses a Strategy I uptake involving root Fe (III) reductase and proton extrusion activity for Fe acquisition^62^. A decreased reduction of Fe will decrease Fe uptake and induce Fe deficiency^61^. Interestingly, Fe deficiency can be considered a stress factor in itself that induces ROS production in plants^63^, indicating the potential for positive feedback loops between GMF and ROS levels through stimulation of Fe deficiency in plants.

Analysis of promoter sequences of genes responsive to a 10 min NNMF exposure showed that NAC transcription factors can be important players in the regulation of genes responsive to MFs (Supplementary Data Set S8). The NAC transcription factors are well recognized as regulators of plant abiotic stress^64^, in agreement with an involvement of NAC in signaling transduction in response to MFs.

### Biphasic dose response of GMF-responsive genes to MF intensities

Our qRT-PCR analysis of expression of five of the six analyzed GMF-response genes at different intensities of MFs showed a non-linear biphasic relationship between gene expression and MF intensity: low doses stimulated or inhibited the expression when compared with near-zero MFs; however, further increases in MF dosage reversed this gene expression. This biphasic response, known as hormesis, is common in a wide range of areas, including cell biology, microbiology, diet/nutrition, medicine, public health, and plant biology, and it is a typical response pattern for a wide range of stimulus types, including toxins, heavy metals, drugs, herbicides, radiation exposure, and others^65–68^. The explanation for a hormetic response is based on the activation of a biological defense mechanism by a low dosage of a stimulus versus the elicitation of stress-induced, non-reparable damage by high doses that ultimately lead to growth inhibition^69^. The fact that very different classes of stimuli can trigger this hormetic response supports the regulation of overlapping sets of stress-responsive genes by these different stimuli, including regulation by MFs. ROS are potential common triggers involved in this biphasic response: a low dosage of ROS would induce defense mechanisms, whereas a high dosage could induce significant damange and ultimately inhibit plant growth.

The reduction in the gene expression responses observed in the range from zero to a 60 µT MF indicates that an optimal level of the GMF might be about 30 µT MF, which is the typical MF for some regions of the earth, such as South America and South Africa. However, the biphasic responses for individual genes do not necessary reflect the growth responses of plants. Indeed, numerous investigations of MF effects on plant growth have shown that strong increases in MFs above the levels normally found on Earth still can have stimulating effects on plant growth^70^. However, at extremely high intensities, MFs inhibit plant growth and the emergence of new leaves^70^, thereby supporting a biphasic response of plant growth to MFs. Taking into consideration that the stimulatory effect of MFs on plant growth is observed across a wide range of intensities and that MFs have a low environmental footprint, the use of MFs has substantial potential for hormetic activation and priming in broad areas, including plant agriculture.

The relatively constant MF dosage during the plant lifespan suggests that plants presumably had no need to evolve mechanisms for quick responses to MF changes. Therefore, we hypothesize that plants recruit signaling cascades that have evolved to respond to other stimuli and stress factors to give rise to the hormetic response. Hence, low stress doses can stimulate plant growth, with ROS playing the role of a common trigger that induces this hormetic response.

Recent findings have provided evidence for an involvement of the radical pair mechanism (thereby implying the modulation of cellular ROS) in magnetoperception in plants, animals, and humans^8,17,26,27^. In plants, appropriate radical pairs are formed by two molecules: cryptochrome and chlorophyll^7^. However, any kind of metabolic or redox enzymes capable of generating ROS can also generate radical pair intermediates^8,26,27^. This includes enzymes operating in mitochondria^71^.

Cryptochrome has been reported to modulate ROS in response to weak magnetic fields through an alteration of the rate of redox reactions in the presence of a magnetic field^27^. This effect changes the cellular ROS production (in the nucleus and cytosol, and possibly also in other organelles) and is proposed to be similar in both plants and animals. Thus, the primary effect of a magnetic field with respect to cryptochrome function has been postulated to be the modulation of ROS^27^. This mechanism perfectly predicts an effect on cellular ROS signaling pathways; therefore, this hypothesis explains the ROS-related modulation of gene expression in response to MFs observed in the present study.

Further studies and strong collaborative efforts are required to deepen our knowledge of plant magnetoperception and transduction.

## Methods

### Plant materials and growth conditions

*Arabidopsis thaliana* ecotype Columbia-0 (*Col-0*) wild type (WT) seeds were surface sterilized with 70% v/v ethanol for 2 min and then with 5% w/v calcium hypochlorite for 5 min. After 3–4 washes with sterile water, seeds were sown on the surface of sterile agar plates (12 × 12 cm) containing half-strength Murashige and Skoog (MS) medium^72^. Plates were sealed with Micropore tape to allow gas exchange and to avoid condensation. Plates were vernalized for 48 h at 4 °C and then exposed vertically under a homogenous and continuous light source at 120 μmol m^−2^ s^−1^ and 22 °C (± 1.5) for 14 h before being kept in the darkness at room temperature for 72 h. Plates were then transferred, in the same laboratory and at the same time, under either NNMF or GMF (controls, see below) and exposed to 120 μmol m^−2^ s^−1^ white light provided by a high-pressure sodium lamp source (SYLVANIA, Grolux 600 W, Belgium) at 22 °C (± 1.5 °C) with a 16/8 light/darkness photoperiod, where germination and plant development occurred. The temperature in the room were the experiments were carried out was controlled and stabilized by air conditioning in order to avoid possible effects of temperature changes during the experiments. All experiments were performed under normal gravity and atmospheric pressure. This work does not invove the collection of plant or seed specimens and complies with the IUCN Policy Statement on Research Involving Species at Risk of Extinction and the Convention on the Trade in Endangered Species of Wild Fauna and Flora.

### Near Null Magnetic Field (NNMF) generation system and plant exposure

The GMF (or local geomagnetic field) values where typical of the Northern hemisphere at 45° 0′ 59″ N and 7° 36′ 58″ E coordinates. Near-null magnetic field (NNMF) was generated by three orthogonal Helmholtz coils connected to three DC power supplies (model E3642A 50 W, 2.5Adual range: 0–-8 V/5A and 0–20 V/2.5 A, 50 W, Agilent Technologies, Santa Clara, CA) controlled by a computer via a GPIB connection^18^. Real-time monitoring of the magnetic field in the plant exposure chamber was achieved with a three-axis magnetic field sensor (model Mag-03, Bartington Instruments, Oxford, U.K.) that was placed at the geometric centre of the Helmholtz coils. The output data from the magnetometer were uploaded to a VEE Pro for Windows software Release 7.51 (Agilent Technologies, https://www.keysight.com/it) to fine-tune the current applied through each of the Helmholtz coil pairs in order to maintain the magnetic field inside the plant growth chamber at NNMF intensity as recently reported^18^. Defining the vertical axis as “y”, the GMF level at the experimental location in our lab was: B_x_ = 6.39 µT, B_y_ = 36.08 µT, B_z_ = 20.40 µT; i.e., a magnetic field strength (B = (Bx^2^ + By^2^ + Bz^2^)^½^) of 41.94 µT; by applying the following voltages Vx = 11.36, Vy = 15.04, Vz = 13.81 (which produced currents I_x_ = 26 mA, I_y_ = 188 mA, I_z_ = 103 mA), the magnetometer values were: B_x_ = 0.033 µT, By = 0.014 µT, Bz = 0.018 µT with a field strength of 40.11 nT, which is about one thousandth of the GMF strength. Plates containing Arabidopsis seedlings were placed in the geometric center of the triaxial Helmholtz coils system and exposed either to NNMF for 10 min, 1 h, 4 h, 24 h, 48 h and 96 h. After the exposure period, shoots and roots were harvested separately and immediately frozen in liquid nitrogen. In order to assess the effect of varying B values on Arabidopsis gene expression, the voltage applied to the three couples of Helmholtz coils was also varied to obtain B values of 240 nT, 11 μT, 18 μT, 21 μT, 34 μT, 41 μT, 50 μT and 60 μT. Sham experiments were performed as detailed previously^18^.

### RNA extraction from Arabidopsis shoots and roots upon time-course exposure to GMF and NNMF

For each time point, 100 mg of frozen Arabidopsis roots and shoots exposed to either GMF or NNMF were ground in liquid nitrogen with mortar and pestle. In details, 6 biological replicates were extracted after 10 min exposure; whereas for all other timings, four biological replicates were extracted. Total RNA was isolated using the Agilent Plant RNA Isolation Mini Kit (Agilent Technologies) and RNase-Free DNase set (Qiagen, Hilden, Germany). Sample quality and quantity was checked by using the RNA 6000 Nano kit and the Agilent 2100 Bioanalyzer (Agilent Technologies) according to manufacturer’s instructions. Quantification of RNA was also confirmed spectrophotometrically by using a NanoDrop ND-1000 (Thermo Fisher Scientific, Waltham, MA, US).

### cDNA synthesis and gene microarray analyses (including MIAME)

Five hundred ng total RNA from each sample were separately reverse-transcribed into double-stranded cDNA by the Moloney murine leukemia virus reverse transcriptase (MMLV-RT) and amplified for 2 h at 40 °C using the Agilent Quick Amp Labelling Kit, two-color (Agilent Technologies). Subsequently, cDNAs were transcribed into antisense cRNA and labelled with either Cy3-CTP or Cy5-CTP fluorescent dyes for 2 h at 40 °C following the manufacturer’s protocol. Cyanine-labeled cRNAs were purified using RNeasy Minikit (Qiagen, Hilden, Germany). Purity and dye incorporation were assessed with the NanoDrop ND-1000 UV–Vis Spectrophotometer (Thermo Fisher Scientific) and the Agilent 2100 Bioanalyzer (Agilent Technologies). Then, 825 ng of control Cy3-RNAs and 825 ng of treated Cy5-RNAs were pooled together and hybridized using the Gene Expression Hybridization Kit (Agilent Technologies) onto 4 × 44 K Arabidopsis (v3) Oligo Microarray (Agilent Technologies). The microarray experiment followed a direct 2 × 2 factorial two-colour design. This resulted in two-colour arrays, satisfying Minimum Information About a Microarray Experiment (MIAME) requirements^73^.

Microarrays were scanned with the Agilent Microarray G2505B Scanner with the extended dynamic range (XDR) scan mode to scan the same slide at two different levels and data were extracted and normalized from the resulting images using Agilent Feature Extraction (FE) software (v.9.5.1) (Agilent Technologies).

GO enrichment information for the differently expressed probe sets was obtained from The Arabidopsis Information Resource (https://www.arabidopsis.org/index.jsp).

### Magnetic field flux density-dependent responses of Arabidopsis selected genes by quantitative Real-Time PCR

The expression of root and shoot Arabidopsis genes selected from the microarray analysis were assayed by quantitative real time PCR (qPCR) and this assay was aimed at both validating the gene microarray and evaluating the gene expression at different magnetic field strengths. First strand cDNA synthesis was accomplished with 1.5 µg total RNA and random primers using the High-Capacity cDNA Reverse Transcription Kit (Applied Biosystems, Foster City, CA, US), according to the manufacturer’s instructions. Briefly, the reactions were prepared by adding 10 µl total RNA (1.5 µg), 2 µl of 10× RT Buffer, 0.8 µl of 25× dNTPs mix (100 mM), 2 µl 10× RT random primer, 1 µl of Multiscribe Reverse Transcriptase and nuclease-free sterile water up to 20 µl. Then the reaction mixtures were subjected to thermal incubation according to the following conditions; 25 °C for 10 min, 37 °C for 2 h, and 85 °C for 5 s.

All qPCR experiments were performed on a Stratagene Mx3000P Real-Time System (La Jolla, CA, USA) using SYBR green I with ROX as an internal loading standard. The reaction was performed with 25 µl of mixture consisting of 12.5 µl of 2× Maxima SYBR Green/ROX qPCR Master Mix (Fermentas International, Inc, Burlington, ON, Canada), 0.5 µl of cDNA and 100 nM primers (Integrated DNA Technologies, Coralville, IA, US). Controls included non-RT controls (using total RNA without reverse transcription to monitor for genomic DNA contamination) and non-template controls (water template). Fluorescence was read following each annealing and extension phase. All runs were followed by a melting curve analysis from 55 to 95 °C. The linear range of template concentration to threshold cycle value (Ct value) was determined by performing a dilution series using cDNA from three independent RNA extractions analysed in three technical replicates. All primers were designed using Primer 3 software^74^ (See Supplementary Table S3 for primers description). Primer efficiencies for all primers pairs were calculated using the standard curve method^75^. Four different reference genes (cytoplasmic glyceraldehyde-3-phosphate dehydrogenase, (*GAPC2*, *At1g13440*), ubiquitin specific protease 6 (*UBP6*, *At1g51710*), β-adaptin (*At4g11380*) and the elongation factor 1B alpha-subunit 2 (*eEF1Balpha2*, *At5g19510*)) were used to normalize the results of the real time PCR. The best of the four genes was selected using the Normfinder software^76^; the most stable gene was the *elongation factor 1B alpha-subunit 2*.

All amplification plots were analyzed with the MX3000P software to obtain Ct values. Relative RNA levels were calibrated and normalized with the level of the elongation factor 1B alpha-subunit 2 mRNA.

### Statistical analyses

The data obtained from qPCR were treated by using Systat 10. Mean value was calculated along with the SD. Paired t test and Bonferroni adjusted probability were used to assess the difference between treatments and the control. Processing and statistical analysis of the microarray data were done in R using Bioconductor package limma^77^. The raw microarray data are subjected to background subtraction and loess normalized. Agilent control probes were filtered out. The linear models implemented in limma were used for finding differentially expressed genes. Comparisons were made for each of the treatment. Benjamini and Hochberg (BH) multiple testing correction was applied.

## Supplementary Information

Supplementary Information 1.

Supplementary Information 2.
