# Supplementary material for: Differential root and shoot magnetoresponses in Arabidopsis thaliana
Source: Sci Rep. 2021 Apr 28;11:9195. doi: 10.1038/s41598-021-88695-6 (PMC8080623; doi:10.1038/s41598-021-88695-6)
Supplement: Supplementary file 1 — Supplementary Information 1. [file 41598_2021_88695_MOESM1_ESM.pdf]

Differential root and shoot magnetoresponses in *Arabidopsis thaliana*

Ivan Paponov[1], Judith Fliegmann[2**], Ravishankar Narayana[3], and Massimo E. Maffei[4*]**

[1] Department of Food Science, Aarhus University, Aarhus, Denmark.
[2] ZMBP Center for Plant Molecular Biology, University of Tübingen, Tübingen, Germany (https://orcid.org/0000-0003-1021-8006).
[3]Citrus Research and Education Center, University of Florida - Lake Alfred, FL, USA
[4] Plant Physiology Unit. Dept. Life Sciences and Systems Biology. University of Turin, Turin, Italy

SUPPLEMENTARY TABLES S1, S2 and S3

And

SUPPLEMENTARY DATA SETS

S1, S2, S3, S4, S5, S6, S8

Supplementary Table S1

Gene expression changes, statistical analysis and fold change volcano plots for all time points assessed

10 min

Test Description

Selected Test : T Test Against Zero
p-value computation: Asymptotic
Multiple Testing Correction: Benjamini-Hochberg

Result Summary

	P all	P < 0.05	P < 0.02	P < 0.01	P < 0.0050	P < 0.0010
FC all	37538	10811	9153	8207	7362	5769
FC > 1.1	18950	10307	8932	8093	7297	5753
FC > 1.5	9827	8982	8181	7616	6974	5636
FC > 2.0	3654	3591	3499	3392	3219	2809
FC > 3.0	142	136	120	93	52	21
Expected by ch...		540	183	82	36	5

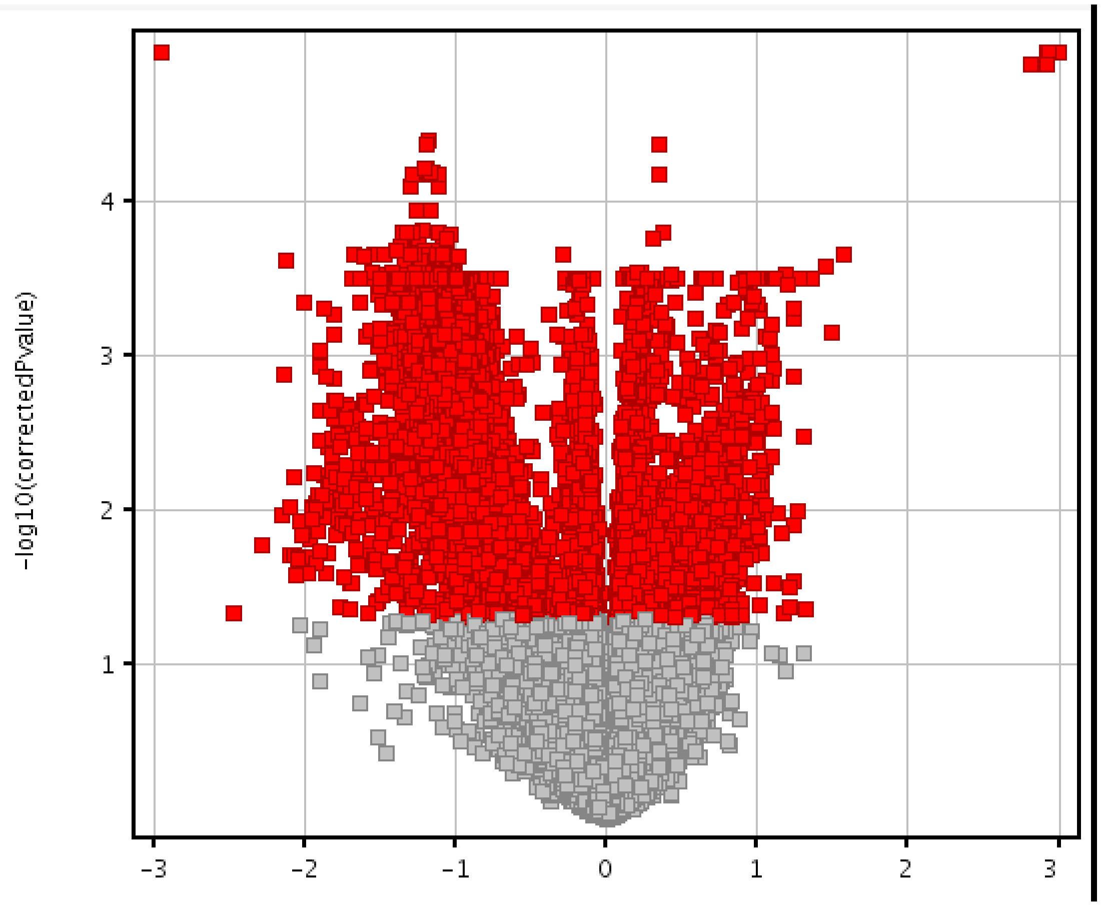

1 h

Test Description

Selected Test : T Test Against Zero
p-value computation: Asymptotic
Multiple Testing Correction: Benjamini-Hochberg

Result Summary

	P all	P < 0.05	P < 0.02	P < 0.01	P < 0.0050	P < 0.0...
FC all	37538	10321	8725	7887	7212	5997
FC > 1.1	20051	9900	8570	7810	7176	5991
FC > 1.5	10063	8593	7915	7423	6960	5939
FC > 2.0	6713	6653	6551	6431	6294	5698
FC > 3.0	29	25	24	21	19	15
Expected ...		516	174	78	36	5

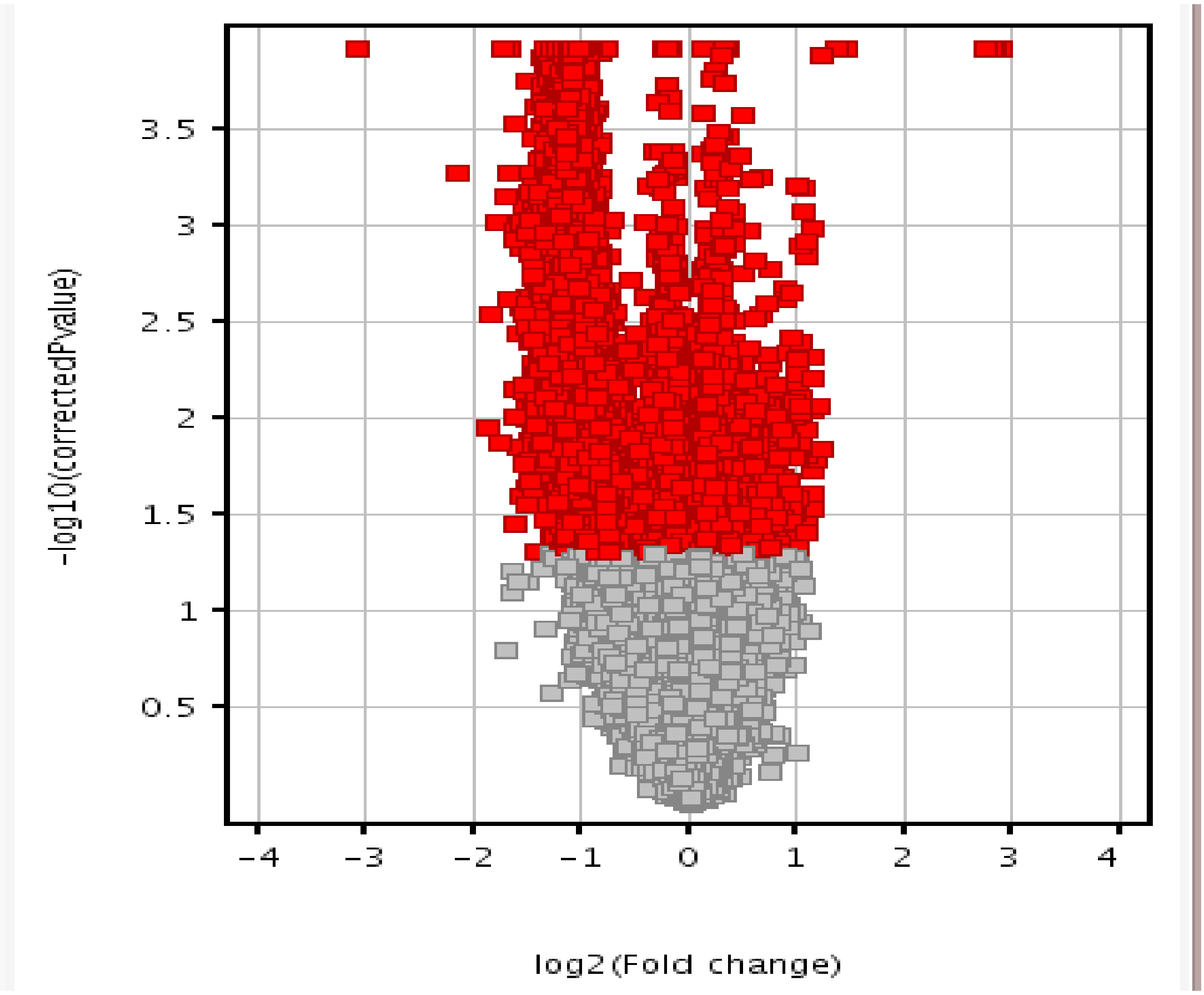

2 h

Test Description

Selected Test : T Test Against Zero
p-value computation: Asymptotic
Multiple Testing Correction: Benjamini-Hochberg

Result Summary

	P all	P < 0.05	P < 0.02	P < 0.01	P < 0.0050	P < 0.0010
FC all	37538	10876	9084	8123	7363	6133
FC > 1.1	18830	10118	8760	7950	7288	6119
FC > 1.5	9748	8521	7812	7319	6883	5976
FC > 2.0	6286	6248	6174	6086	5974	5612
FC > 3.0	21	19	18	15	15	13
Expected by ch...		543	181	81	36	6

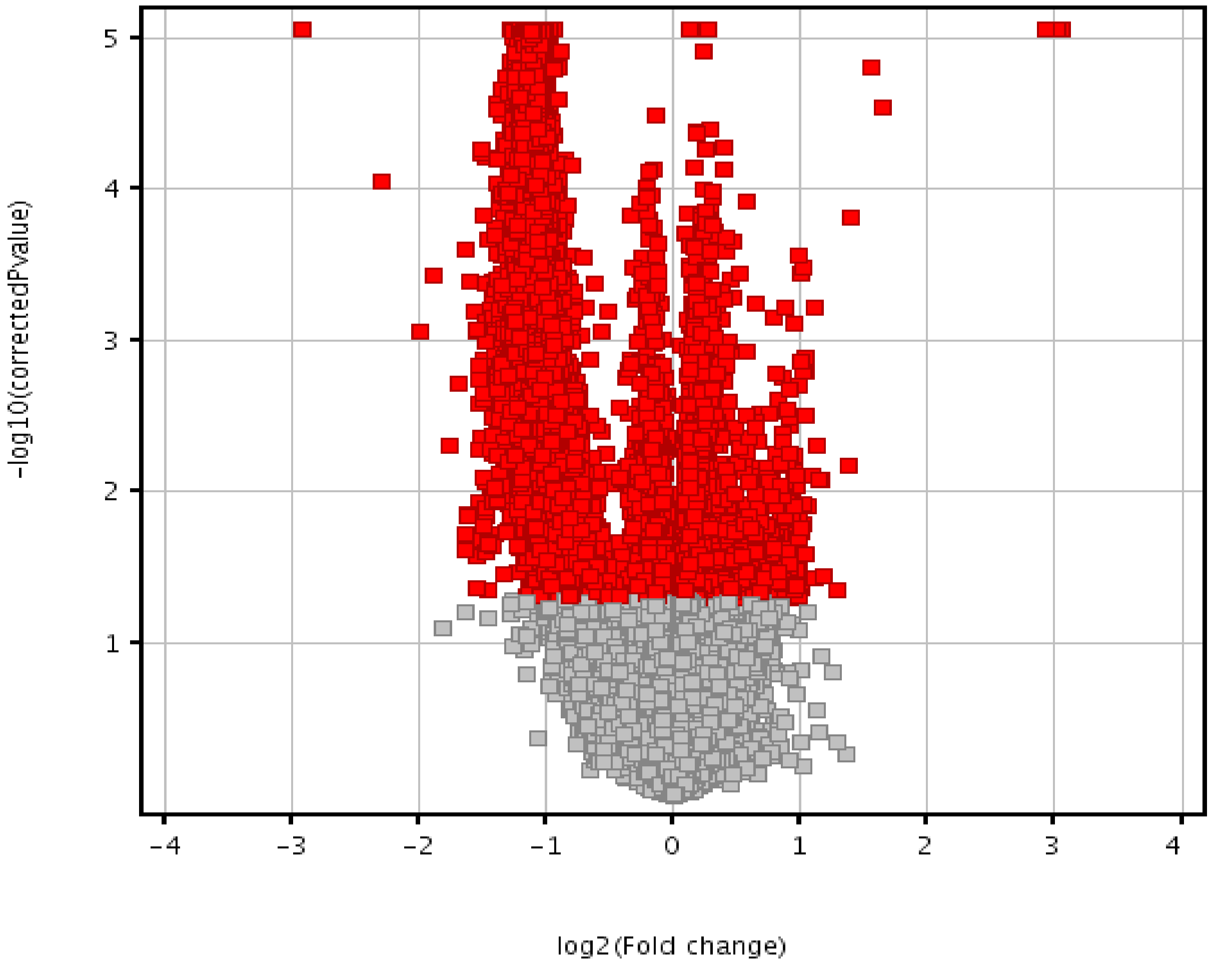

4 h

Test Description

Selected Test : T Test Against Zero
p-value computation: Asymptotic
Multiple Testing Correction: Benjamini-Hochberg

Result Summary

	P all	P < 0.05	P < 0.02	P < 0.01	P < 0.0050	P < 0.0010
FC all	37538	11135	9057	7982	7136	5759
FC > 1.1	21896	10709	8901	7908	7098	5753
FC > 1.5	9557	8557	7826	7298	6770	5666
FC > 2.0	2056	2018	1965	1904	1804	1464
FC > 3.0	21	16	14	14	13	11
Expected by ch...		556	181	79	35	5

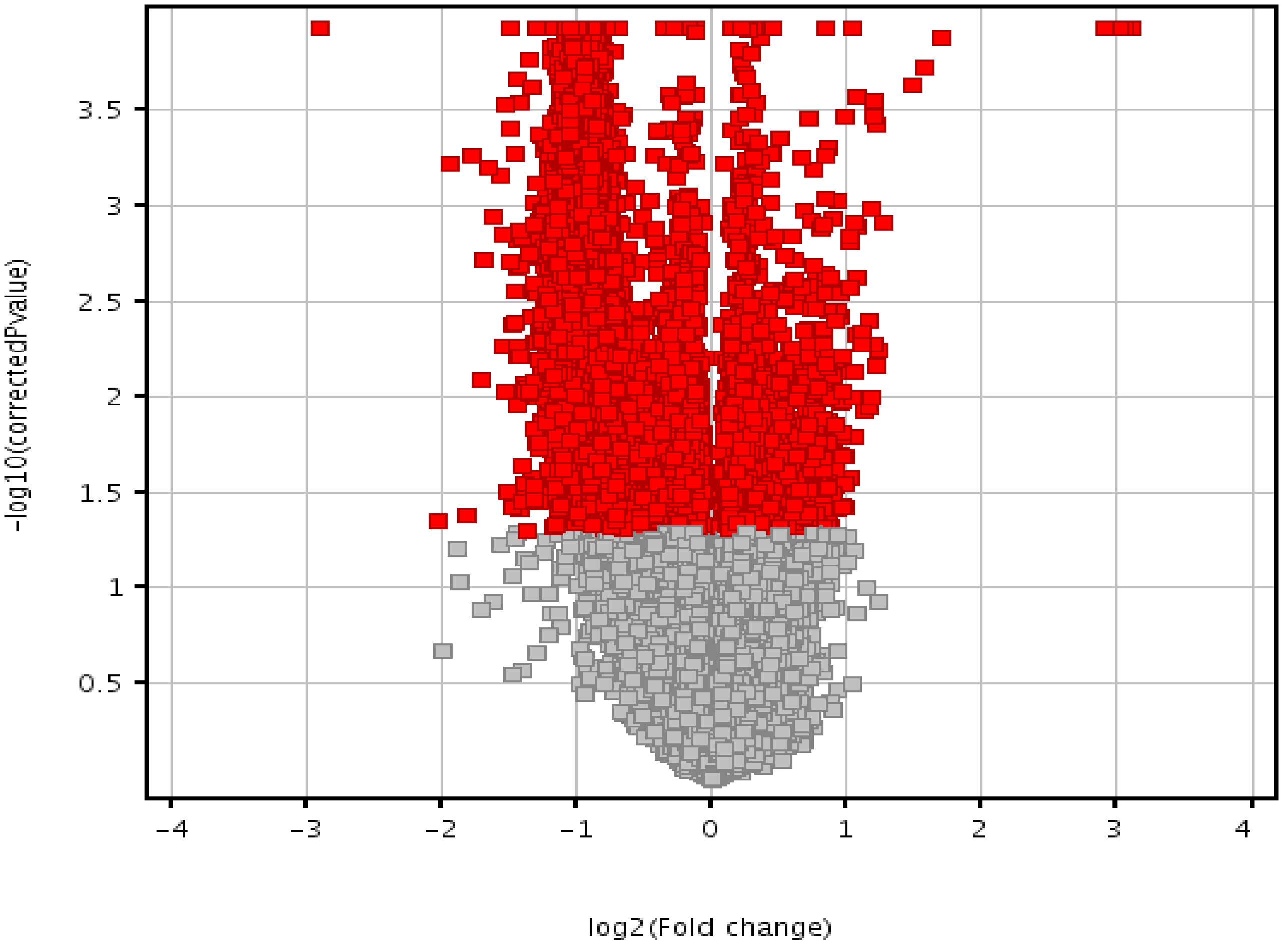

24 h

Test Description

Selected Test : T Test Against Zero
p-value computation: Asymptotic
Multiple Testing Correction: Benjamini-Hochberg

Result Summary

	P all	P < 0.05	P < 0.02	P < 0.01	P < 0.0050	P < 0.0010
FC all	37538	7984	6861	6171	5597	0
FC > 1.1	19702	7900	6836	6163	5590	0
FC > 1.5	9544	7443	6648	6065	5539	0
FC > 2.0	4244	4104	3972	3849	3712	0
FC > 3.0	12	11	10	9	9	0
Expected by ch...		399	137	61	27	0

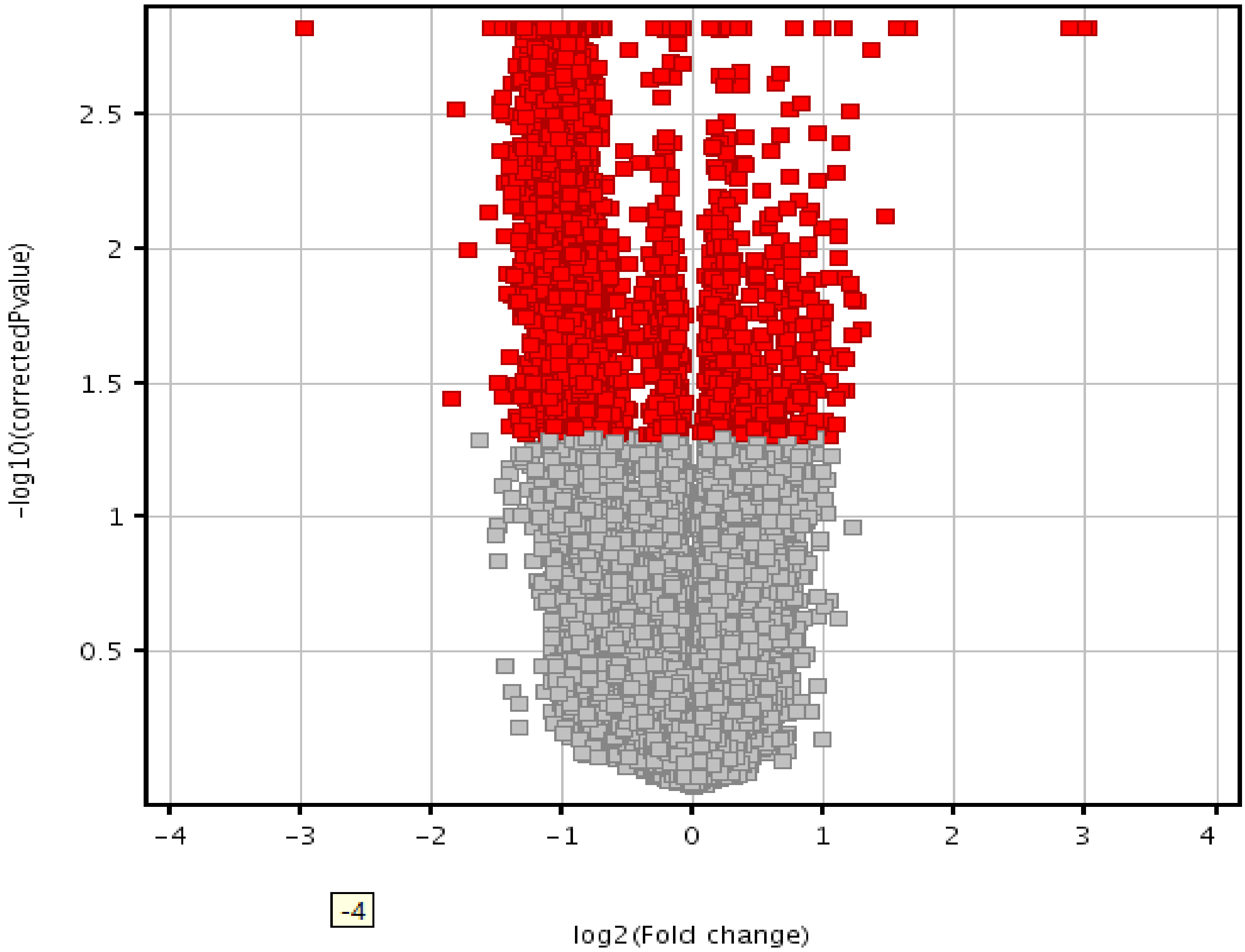

48 h

Test Description

Selected Test : T Test Against Zero
p-value computation: Asymptotic
Multiple Testing Correction: Benjamini-Hochberg

Result Summary

	P all	P < 0.05	P < 0.02	P < 0.01	P < 0.0050	P < 0.0010
FC all	37538	10963	8932	7808	6932	5512
FC > 1.1	20281	10246	8621	7643	6843	5496
FC > 1.5	9676	8202	7418	6830	6304	5285
FC > 2.0	6565	6522	6418	6225	5957	5201
FC > 3.0	49	48	45	43	39	23
Expected by ch...		548	178	78	34	5

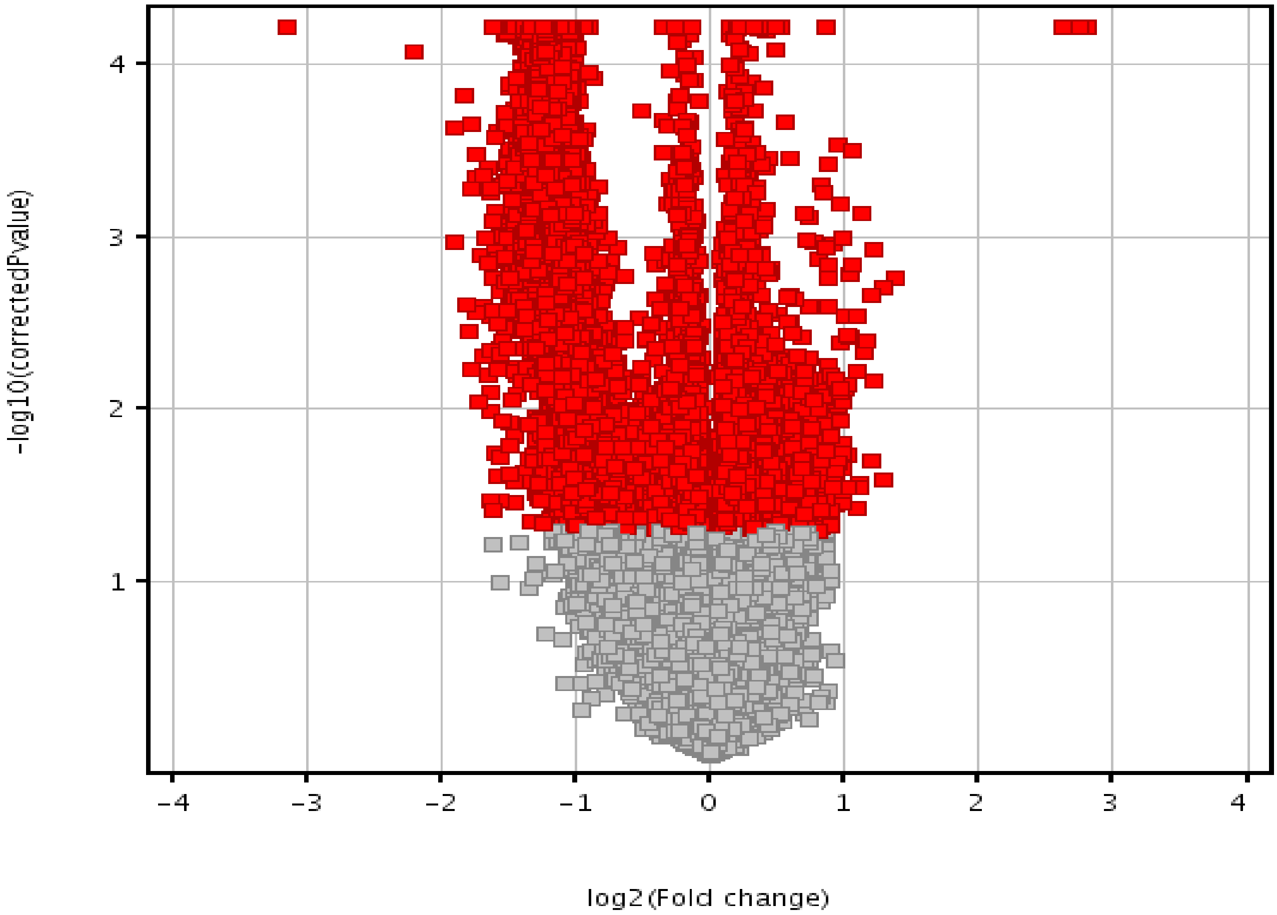

96 h

Test Description

Selected Test : T Test Against Zero
p-value computation: Asymptotic
Multiple Testing Correction: Benjamini-Hochberg

Result Summary

	P all	P < 0.05	P < 0.02	P < 0.01	P < 0.0050	P < 0.0010
FC all	37538	8124	6555	5751	5058	0
FC > 1.1	21772	7853	6446	5699	5037	0
FC > 1.5	9940	6966	6042	5458	4918	0
FC > 2.0	5991	5662	5350	4996	4613	0
FC > 3.0	61	43	34	25	15	0
Expected by ch...		406	131	57	25	0

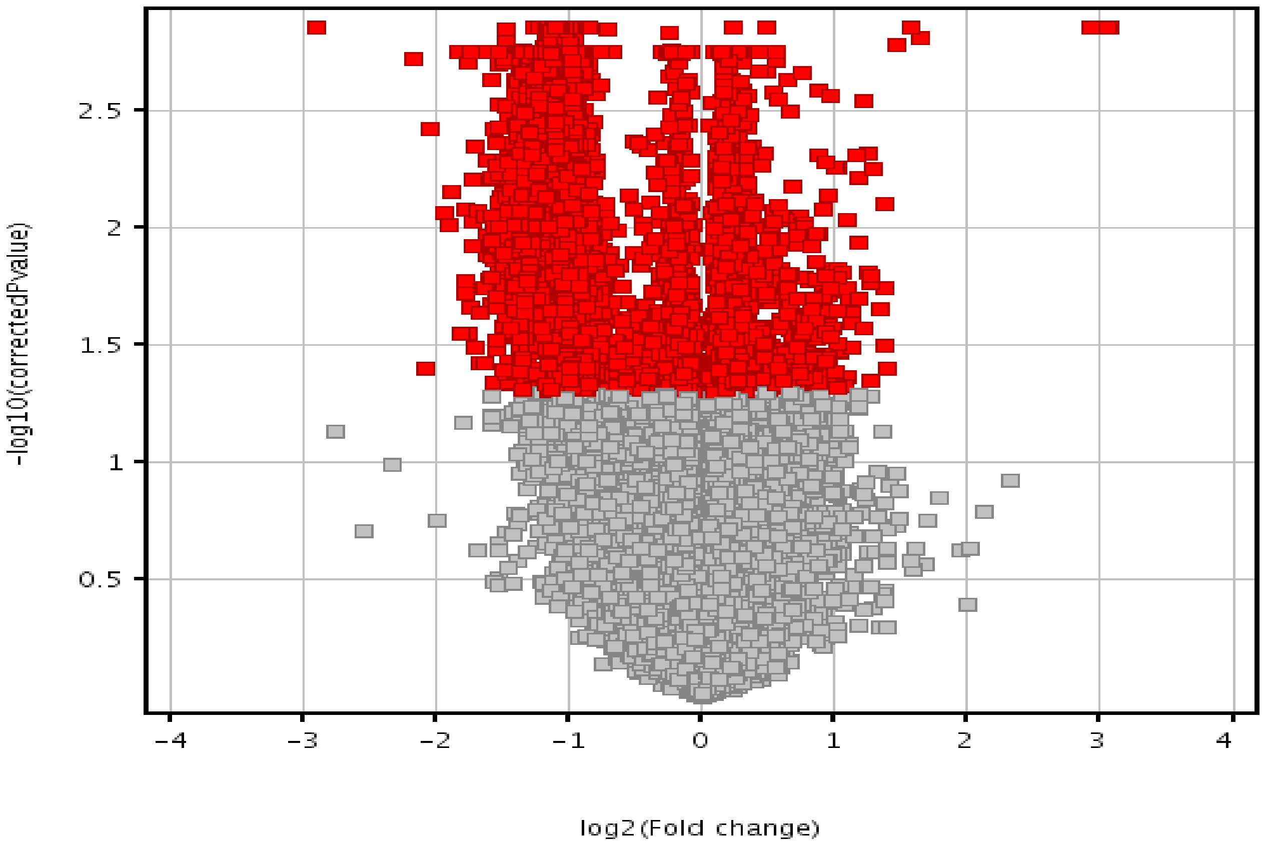

Supplementary Table S2. GeneSpring analysis of gene expression from a time course analysis of Arabidopsis thaliana seedlings exposed to GMF (control) and NNMF.

**Specification**	**Time-course point**						
	**10 min**	**1 h**	**2 h**	**4 h**	**24 h**	**48 h**	**96 h**
Number of Biological replicates retained in the analysis (total number)	10 (12)	7 (8)	8 (8)	8 (8)	7 (8)	8 (8)	6 (8)
Number of genes satisfying a corrected P-value cut-off of 0.05 (% out of 37538 genes)	10,811 (29%)	10,321 (27%)	10,876 (29%)	11,135 (30%)	7,984 (21%)	10,963 (29%)	8,124 (22%)
Number of genes with FC > 2 and P < 0.05 (% out of satisfying genes)	3,591 (33%)	6,653 (64%)	6,248 (57%)	2,018 (18%)	4,104 (51%)	6,522 (59%)	5,662 (70%)
Number of genes with FC > 3 and P < 0.05	136	25	19	16	11	48	43

Supplementary Table S3

Primers used in quantitative real time PCR experiments

Gene Code	Gene	Forward primer (5'-3')	Reverse primer (5'-3')
**Reference genes**			
At1g13440	*GAPC2*	TCAGGAACCCTGAGGACATC	CGTTGACACCAACAACGAAC
At1g51710	*UBP6*	GAAAGTGGATTACCCGCTG	CTCTAAGTTTCTGGCGAGGAG
At4g11380	*β-adaptin*	GAAGACTGCGGCTATTTGCG	CTTGACCCCACTCAGTGCAT
At5g19510	*eEF1Balpha2*	ACTTGTACCAGTTGGTTATGGG	CTGGATGTACTCGTTGTTAGGC
**Target genes**			
At1g19510	*RAD-LIKE 5 (RADIALIS-LIKE SANT/MYB 4)*	GCTCGAGCTCTTCTTGGAC	CGAGGATGTCGTAGTGACGTT
At4g18335	*unknown*	CATCGTGCTGGCTACTTCAA	AGCGGAATTAGGGTTCTGG
At4g25140	*OLEO1*	ACCCACAGGGATCAGACAAG	CCCGACATTGGAGTTATTGG
At4g28520	*CRU3*	TTCCTCCTCAGCTACAGAACG	GAAGGTGGGCAAGTAAAGACC
At5g09720	*Mg transporter CorA-like family protein*	CTCCGGATACTTGACCCAAA	AAAGGACCGAAACACACAGG
At5g60130	*AP2/B3-like transcriptional factor family protein*	AGCGAGGAGATCAAAGTGGA	CTCGACAGCAGCTTCATCAG

SUPPLEMENTARY DATA SET S1: GENE EXPRESSION

Group A: Genes upregulated in shoot at middle times and downregulated in the shoot at longer times

		Fold Change														p value													
		ROOTS							SHOOTS							ROOTS							SHOOTS						
		EARLY			MIDDLE		LATE		EARLY			MIDDLE		LATE		EARLY			MIDDLE		LATE		EARLY			MIDDLE		LATE	
		10m	1h	2h	4h	24h	48	96h	10m	1h	2h	4h	24h	48	96h	10m	1h	2h	4h	24h	48	96h	10m	1h	2h	4h	24h	48	96h
Gene Code	Gene model																												
A_84_P23651	At1g03840.1	1.063	1.073	1.028	1.123	0.961	0.991	0.959	0.913	1.146	1.017	1.194	1.242	0.991	0.778	0.33	0.372	0.716	0.198	0.649	0.912	0.59	0.3	0.14	0.91	0.0762	0.202	0.028	0.0255
A_84_P586130	At1g05240.1	1.046	0.963	1.17	1.09	0.883	1.417	1.049	1.182	0.838	1.386	1.423	1.423	1.417	0.638	0.6	0.627	0.0897	0.463	0.356	0.0452	0.698	0.329	0.559	0.373	0.528	0.0145	0.0408	0.0766
A_84_P277420	At1g05250.1	0.994	0.924	1.109	1.046	0.843	1.399	1.039	1.194	0.724	1.839	1.092	1.355	1.399	0.586	0.937	0.324	0.216	0.711	0.258	0.0361	0.767	0.205	0.394	0.353	0.91	0.032	0.0759	0.0484
A_84_P11901	At1g06100.1	0.977	0.957	0.983	0.935	0.877	1.451	0.816	0.721	1.539	0.951	1.625	1.076	1.451	0.71	0.738	0.597	0.824	0.419	0.543	0.064	0.524	0.167	0.0614	0.738	0.0172	0.484	0.0248	0.0303
A_84_P18992	At1g15210.1	1.046	1.085	0.97	1.125	0.992	0.985	0.96	1.016	1.036	1.239	1.126	1.172	0.985	0.733	0.53	0.328	0.716	0.27	0.953	0.846	0.67	0.852	0.647	0.0755	0.431	0.37	0.0433	0.0205
A_84_P159165	At1g17030.1	1.125	1.077	0.828	0.801	0.7	1.604	1.02	1.318	1.235	1.027	1.467	1.603	1.604	1.236	0.325	0.643	0.167	0.181	0.318	0.0966	0.795	0.155	0.111	0.916	0.016	0.0145	0.685	0.721
A_84_P127531	At1g21310.1	1.025	0.947	1.121	1.025	1.123	0.918	1.005	0.961	0.999	1.13	1.405	1.333	0.918	0.846	0.741	0.485	0.181	0.773	0.237	0.301	0.955	0.793	0.991	0.727	0.0363	0.0264	0.0282	0.354
A_84_P294984	At1g24735.1	1.109	1.178	1.026	1.02	0.928	1.049	0.906	1.001	0.926	1.021	1.233	0.99	1.049	0.759	0.124	0.202	0.738	0.807	0.408	0.607	0.292	0.992	0.488	0.801	0.0554	0.933	0.035	0.0242
A_84_P16071	At1g33750.1	1.023	1.065	0.941	1.282	1.017	1.018	1.009	1.154	0.841	1.389	1.232	1.289	1.018	0.525	0.789	0.423	0.443	0.107	0.899	0.809	0.935	0.223	0.28	0.285	0.579	0.429	0.0192	0.0394
A_84_P223359	At1g54000.1	1.09	1.083	0.867	1.205	0.906	1.043	0.849	0.917	0.951	1.375	1.154	1.371	1.043	0.588	0.392	0.367	0.14	0.0642	0.368	0.586	0.216	0.464	0.764	0.367	0.57	0.21	0.0197	0.0132
A_84_P14146	At1g56680.1	1.035	0.927	0.946	1.262	0.873	0.924	1.179	0.859	0.857	1.107	0.953	1.232	0.924	0.496	0.757	0.508	0.486	0.0658	0.248	0.458	0.0821	0.25	0.47	0.524	0.828	0.175	0.0385	0.00143
A_84_P14033	At1g66270.1	0.986	1.194	0.884	1.213	0.889	1.086	0.727	0.848	1.032	1.639	1.153	1.426	1.086	0.526	0.912	0.132	0.276	0.11	0.385	0.468	0.081	0.328	0.861	0.139	0.602	0.336	0.0293	0.0152
A_84_P11390	At1g66280.1	1.09	1.135	0.939	1.113	1.019	1.135	0.781	0.927	1.227	1.393	1.356	1.392	1.135	0.57	0.268	0.167	0.422	0.215	0.841	0.183	0.209	0.55	0.225	0.326	0.238	0.379	0.0105	0.0398
A_84_P237923	At1g68470.1	1.097	1.087	1.078	1.079	0.799	0.947	0.976	1.01	0.999	1.009	1.35	1.122	0.947	0.895	0.208	0.347	0.478	0.384	0.0384	0.671	0.902	0.914	0.99	0.917	0.0162	0.368	0.861	0.297
A_84_P18104	At1g74500.1	1.018	1.014	0.988	1.259	0.94	1.055	1.016	0.796	0.898	1.222	1.301	1.278	1.055	0.516	0.816	0.882	0.891	0.0561	0.484	0.498	0.912	0.293	0.679	0.406	0.334	0.308	0.0447	0.0434
A_84_P15447	At2g25980.1	0.984	1.045	0.974	1.16	0.938	1.029	0.932	0.914	1.001	1.445	1.635	1.024	1.029	0.527	0.866	0.629	0.739	0.256	0.596	0.704	0.564	0.553	0.995	0.243	0.17	0.925	0.00843	0.0284
A_84_P19161	At2g32860.1	1.015	1.397	0.816	1.34	0.712	1.042	0.863	0.998	1.849	1.095	1.049	0.957	1.042	0.953	0.923	0.137	0.154	0.0221	0.0331	0.76	0.135	0.992	0.0525	0.748	0.731	0.762	0.885	0.808
A_84_P302850	At2g37870.1	0.96	1.145	0.995	1.207	0.938	1.293	1.092	0.79	0.958	0.907	1.978	1.081	1.293	1.123	0.596	0.189	0.946	0.0718	0.65	0.0267	0.638	0.0961	0.808	0.317	0.0443	0.598	0.0422	0.652
A_84_P21103	At2g41480.1	1.047	1.132	0.961	1.003	1.026	0.97	0.737	0.931	1.137	1.078	1.304	1.322	0.97	0.954	0.697	0.242	0.676	0.987	0.744	0.74	0.0183	0.559	0.28	0.353	0.0317	0.0266	0.503	0.684
A_84_P73044	At2g41660.1	1.015	0.985	0.992	0.948	0.988	1.085	1.115	1.026	1.105	1.22	1.153	1.071	1.085	0.771	0.809	0.84	0.92	0.523	0.888	0.313	0.216	0.837	0.311	0.0941	0.143	0.447	0.0338	0.041
A_84_P160753	At2g41800.1	0.992	1.021	0.914	1.339	0.869	1.033	0.809	0.886	1.07	1.107	1.256	1.085	1.033	0.543	0.942	0.831	0.317	0.051	0.253	0.669	0.342	0.36	0.563	0.501	0.362	0.705	0.0123	0.0333
A_84_P308363	At2g45750.1	0.968	0.989	1.133	1.148	0.984	1.15	0.992	0.941	1.22	1.165	1.084	0.995	1.15	0.75	0.652	0.882	0.151	0.143	0.836	0.193	0.928	0.428	0.111	0.257	0.534	0.953	0.0402	0.0175
A_84_P22110	At3g08770.1	0.873	0.908	1.054	1.349	0.915	0.735	1.375	0.813	1.178	0.994	1.275	1.266	0.735	0.819	0.147	0.305	0.688	0.0361	0.603	0.117	0.333	0.0497	0.167	0.931	0.0237	0.122	0.282	0.0721
A_84_P23103	At3g14940.1	1.053	1.102	1.042	0.977	1.075	1.01	1.121	1.109	1.024	0.939	0.948	1.307	1.01	0.742	0.402	0.242	0.609	0.784	0.362	0.894	0.225	0.411	0.824	0.606	0.493	0.278	0.0288	0.0421
A_84_P500433	At3g16410.1	1.007	1.092	0.949	1.193	1.013	1.1	0.972	1.07	0.7	1.235	1.589	1.209	1.1	0.706	0.923	0.318	0.516	0.0925	0.917	0.283	0.744	0.728	0.112	0.409	0.156	0.551	0.0242	0.032
A_84_P558095	At3g23175.1	1.073	1.003	1.092	1.005	1.071	1.049	1.033	0.936	0.961	0.969	0.968	1.401	1.049	0.681	0.277	0.965	0.278	0.952	0.39	0.556	0.671	0.533	0.804	0.82	0.8	0.152	0.0488	0.0382
A_84_P22161	At3g24300.1	1.114	0.993	1.081	0.928	1.06	0.981	0.975	0.988	1.403	1.263	1.163	1.619	0.981	0.722	0.109	0.933	0.368	0.647	0.459	0.842	0.845	0.919	0.023	0.4	0.138	0.127	0.0363	0.0243
A_84_P286640	At3g48510.1	1.086	1.108	1.127	1.154	0.815	0.744	1.072	0.858	1.065	1.273	1.817	1.032	0.744	0.817	0.506	0.457	0.214	0.39	0.216	0.0445	0.414	0.169	0.647	0.0338	0.000778	0.933	0.03	0.384
A_84_P17486	At3g49760.1	1.06	1.067	1.045	1.061	1.055	1.096	0.886	0.985	0.769	0.748	1.717	1.913	1.096	0.602	0.354	0.408	0.569	0.532	0.554	0.265	0.228	0.938	0.185	0.34	0.15	0.319	0.0467	0.0451
A_84_P274590	At3g53650.1	1.012	0.92	1.049	1.083	0.977	1.039	0.995	0.952	0.975	1.063	1.015	1.045	1.039	0.779	0.847	0.364	0.561	0.324	0.766	0.661	0.952	0.561	0.736	0.439	0.851	0.783	0.0244	0.0356
A_84_P723766	At3g55850.2	0.935	0.967	1.071	0.717	0.951	1.088	1.07	0.908	1.014	1.044	1.3	1.031	1.088	0.951	0.447	0.866	0.513	0.0153	0.725	0.385	0.444	0.558	0.936	0.7	0.0176	0.768	0.171	0.621
A_84_P734406	At3g55850.2	0.97	0.985	1.133	0.753	1.002	1.141	1.125	0.938	1.049	1.091	1.402	1.052	1.141	0.981	0.72	0.937	0.239	0.0246	0.989	0.193	0.193	0.701	0.778	0.476	0.00699	0.606	0.225	0.854
A_84_P22264	At3g60870.1	1.032	0.993	0.936	1.309	0.971	0.992	1.294	1.036	0.931	1.009	1.175	1.423	0.992	0.777	0.776	0.93	0.463	0.0902	0.828	0.939	0.0224	0.822	0.722	0.961	0.375	0.112	0.0454	0.193
A_84_P541401	At4g18425.1	0.99	1.099	0.71	1.128	0.676	1.701	0.868	1.157	1.071	0.923	1.288	1.343	1.701	0.984	0.884	0.521	0.0833	0.425	0.278	0.119	0.376	0.237	0.561	0.531	0.0344	0.0137	0.875	0.958
A_84_P23288	At4g22810.1	1.033	0.928	0.98	1.025	1.009	1.015	1.013	0.943	1.127	1.303	1.239	1.402	1.015	0.619	0.603	0.351	0.789	0.779	0.923	0.846	0.867	0.694	0.48	0.204	0.114	0.301	0.0188	0.0303
A_84_P12888	At4g23600.1	1.315	0.784	0.886	2.275	1.066	1.078	0.972	0.932	1.238	0.819	1.742	1.171	1.078	1.079	0.302	0.492	0.724	0.0474	0.865	0.819	0.963	0.66	0.141	0.0819	0.00883	0.143	0.0546	0.591
A_84_P183184	At4g26320.1	1.049	1.002	1.114	1.048	0.94	1.039	0.858	1.161	1.011	1.147	1.338	1.183	1.039	0.625	0.452	0.978	0.201	0.603	0.536	0.654	0.225	0.347	0.959	0.23	0.171	0.513	0.0186	0.0187
A_84_P268810	At4g27560.1	1.039	1.003	1.01	1.169	0.885	0.913	1.066	1.004	0.973	0.989	1.316	1.246	0.913	0.861	0.572	0.973	0.891	0.0921	0.259	0.41	0.781	0.957	0.716	0.885	0.029	0.0328	0.177	0.255
A_84_P17743	At5g12970.1	1.002	0.963	1.105	1.018	0.982	0.953	0.973	0.873	1.122	1.072	1.333	1.053	0.953	0.72	0.973	0.639	0.232	0.827	0.845	0.537	0.723	0.0957	0.206	0.426	0.0162	0.571	0.0277	0.0164
A_84_P146818	At5g37940.1	0.95	1.144	0.983	1.17	0.988	1.078	0.862	0.984	0.814	0.883	1.693	1.243	1.078	0.902	0.636	0.34	0.839	0.0897	0.973	0.395	0.424	0.876	0.214	0.44	0.0485	0.0354	0.534	0.359
A_84_P11171	At5g37970.1	1.068	1.224	0.972	1.221	1.02	1.185	0.75	0.723	0.723	0.712	1.98	1.147	1.185	0.652	0.46	0.214	0.788	0.0477	0.95	0.0859	0.0765	0.0612	0.0355	0.113	0.0265	0.877	0.477	0.169
A_84_P13061	At5g37990.1	1.092	0.936	0.995	0.848	1.094	1.013	0.956	1.15	1.117	1.59	1.105	1.512	1.013	0.568	0.391	0.221	0.854	0.0338	0.975	0.0686	0.045	0.0682	0.0939	0.397	0.0103	0.561	0.213	0.213
A_84_P23490	At5g45070.1	1.139	0.97	1.122	1.435	1.08	0.832	1.182	0.91	1.364	0.958	1.95	1.075	0.832	0.771	0.366	0.456	0.969	0.276	0.286	0.869	0.776	0.312	0.494	0.398	0.585	0.245	0.0172	0.0377
A_84_P16890	At5g50800.1	1.135	1.044	1.229	1.016	1.06	0.913	1.613	1.247	1.26	1.375	1.216	3.061	0.913	0.46	0.127	0.824	0.38	0.0111	0.451	0.223	0.444	0.645	0.186	0.771	0.0294	0.59	0.0244	0.522
A_84_P14071	At5g56080.1	1.02	0.896	0.927	1.14	0.966	1.057	0.902	0.969	1.048	0.976	1.009	1.022	1.057	0.694	0.0947	0.782	0.361	0.956	0.737	0.519	0.505	0.348	0.532	0.367	0.654	0.000572	0.00605	0.00562
A_84_P14072	At5g56320.1	1.019	1.015	1.081	1.084	1.014	1.103	0.767	0.86	1.192	1.28	1.523	1.295	1.103	0.748	0.797	0.26	0.375	0.226	0.7	0.477	0.491	0.649	0.578	0.747	0.941	0.847	0.026	0.00701
A_84_P17864	At5g58860.1	0.992	1.182	0.828	1.247	0.79	1.217	0.701	1.014	1.398	1.083	1.009	1.009	1.217	0.904	0.749	0.844	0.33	0.323	0.9	0.397	0.0442	0.303	0.169	0.352	0.0472	0.488	0.0104	0.0347
A_84_P15978	At5g60020.1	1.012	1.07	0.985	1.127	0.909	1.159	0.936	1.061	0.857	0.984	1.043	1.219	1.159	0.749	0.934	0.108	0.185	0.0495	0.0419	0.147	0.0293	0.913	0.137	0.592	0.949	0.926	0.591	0.606
A_84_P23572	At5g66280.1	1.088	1.235	0.982	1.242	1.01	1.196	0.722	0.739	0.681	0.801	2.862	1.812	1.196	0.688	0.842	0.394	0.846	0.225	0.273	0.202	0.589	0.622	0.161	0.858	0.803	0.301	0.0461	0.0203

SUPPLEMENTARY DATA SET S1: Functional Characterization

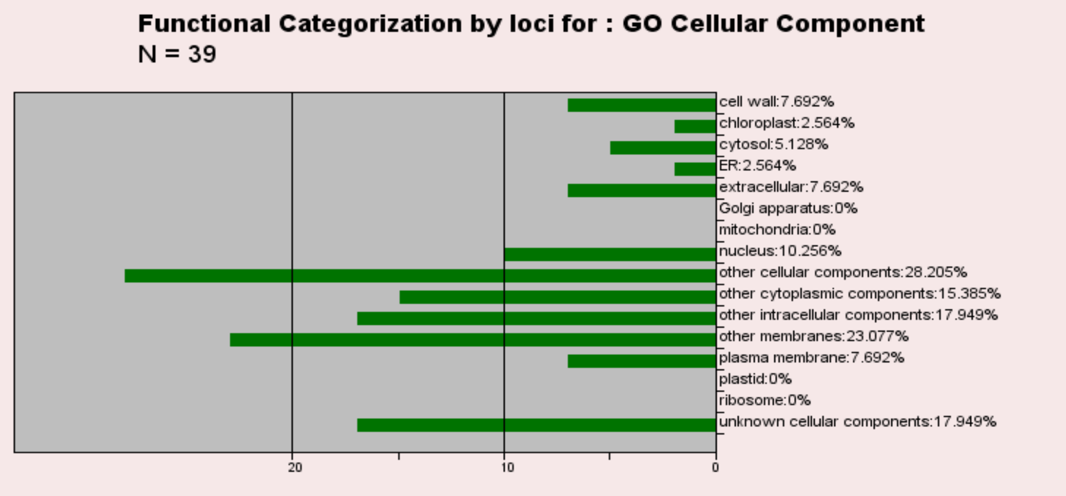

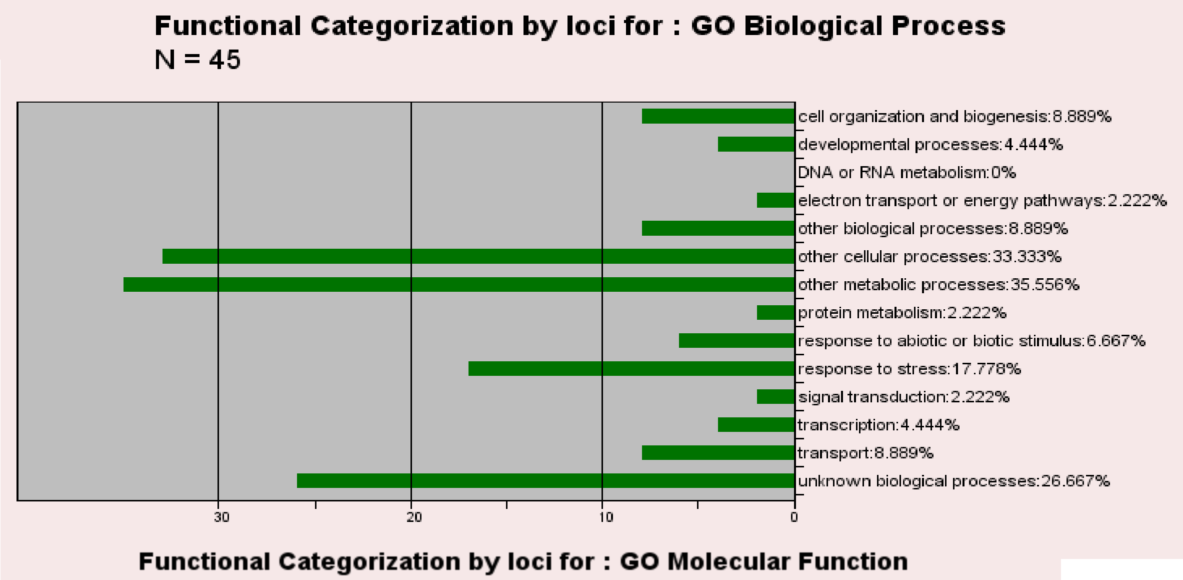

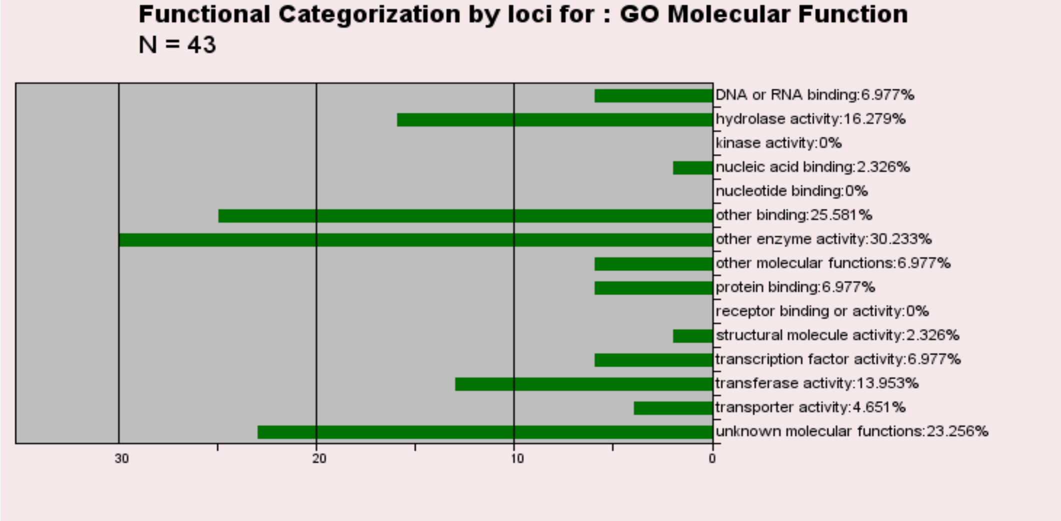

SUPPLEMENTARY DATA SET S1: GO Analysis

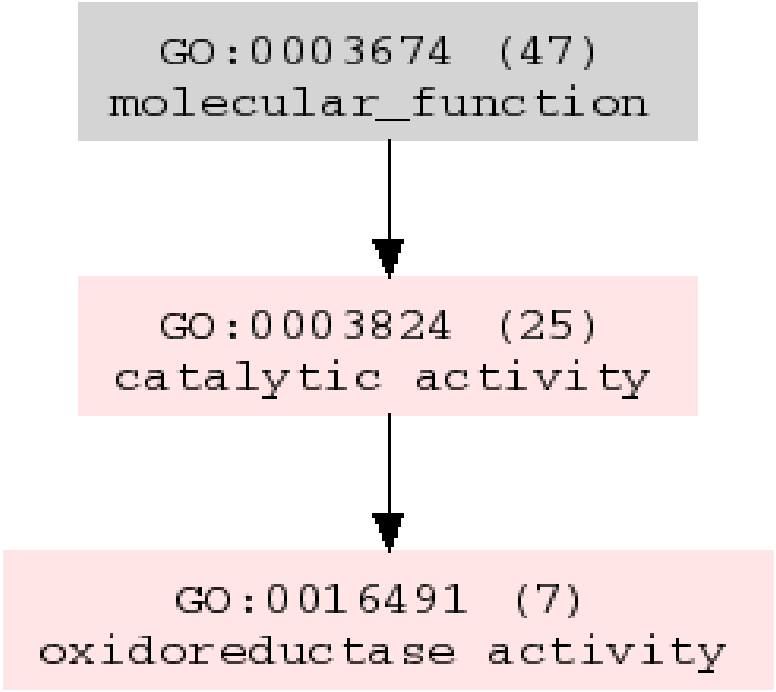

SUPPLEMENTARY DATA SET S2: Gene expression

Group B genes up-regulated in the roots and downregulated in the shoots at all time points

fold change

		ROOTS							SHOOTS						
		EARLY			MIDDLE		LATE		EARLY			MIDDLE		LATE	
Gene Code	Gene model	10m	1h	2h	4h	24h	48	96h	10m	1h	2h	4h	24h	48	96h
A_84_P572778	At1g02040.1	1.786	1.748	1.92	1.955	1.421	1.271	1.559	0.689	0.715	0.591	0.664	0.587	0.602	0.421
A_84_P18004	At1g04670.1	2.05	2.804	2.299	2.045	2.117	3.086	3.093	0.7	0.768	0.731	0.804	0.769	0.628	0.65
A_84_P21815	At1g05577.1	1.191	1.18	1.171	1.38	1.262	1.073	1.246	0.633	0.72	0.661	0.626	0.549	0.782	0.611
A_84_P17085	At1g05650.1	1.213	1.222	1.293	1.374	1.051	2.199	0.847	0.782	0.583	0.562	0.669	0.608	0.825	0.63
A_84_P10574	At1g06990.1	1.774	2.774	1.842	3.058	1.24	1.536	2.532	0.928	0.76	0.723	1.044	0.768	0.609	0.642
A_84_P262310	At1g10880.1	2.226	2.156	2.179	2.359	1.933	1.894	3.338	0.981	0.86	0.829	0.914	0.701	0.687	0.729
A_84_P20852	At1g11920.1	1.996	3.552	2.615	3.073	1.514	1.208	1.17	0.826	0.864	0.83	0.911	0.905	0.701	0.738
A_84_P18909	At1g13310.1	2.564	2.302	1.33	2.511	1.668	2.356	1.471	0.639	0.75	0.611	0.687	0.642	0.487	0.925
A_84_P582608	At1g13540.1	1.826	1.707	2.28	1.674	1.832	1.744	1.356	0.828	0.723	0.686	0.759	0.718	0.578	0.677
A_84_P18086	At1g18410.1	1.376	1.142	1.559	2.278	1.541	2.319	1.576	0.648	0.621	0.68	0.762	0.804	0.56	0.783
A_84_P14044	At1g29540.1	1.081	1.008	1.129	1.103	0.86	1.661	1.342	1.122	0.77	0.833	0.475	1.006	0.896	0.987
A_84_P15241	At1g33890.1	2.616	1.911	1.537	1.655	2.306	1.392	2.037	0.669	0.917	0.682	0.788	0.76	0.511	0.631
A_84_P10830	At1g34540.1	1.174	1.218	1.444	1.543	0.98	2.806	1.432	0.614	0.748	0.77	0.702	0.561	0.755	0.636
A_84_P285470	At1g44030.1	1.711	1.811	1.628	1.436	1.364	1.566	1.026	0.59	0.598	0.592	0.616	0.702	0.53	0.494
A_84_P541828	At1g48070.1	1.348	1.65	1.413	1.756	1.533	1.896	1.244	0.798	0.684	0.777	0.88	0.768	0.685	0.613
A_84_P13245	At1g51220.1	1.95	1.918	1.54	2.753	2.042	1.467	1.597	1.031	0.887	0.852	0.814	0.858	0.787	0.71
A_84_P521460	At1g52240.1	1.427	1.501	1.567	1.787	1.336	2.804	1.097	0.714	0.753	0.72	0.81	0.813	0.654	0.506
A_84_P19775	At1g55230.1	1.575	1.164	1.74	2.585	1.635	1.441	1.109	0.625	0.502	0.552	0.584	0.768	0.822	0.783
A_84_P605885	At1g55990.1	1.317	1.775	1.445	1.27	1.022	1.398	1.122	0.731	0.536	0.707	0.941	0.687	0.658	0.625
A_84_P14049	At1g56160.1	1.836	1.747	1.616	1.626	1.2	2.172	1.348	0.805	1.099	1.333	0.793	0.666	0.563	0.609
A_84_P572925	At1g58643.1	2.054	1.113	1.219	1.532	2.031	2.02	2.572	0.78	0.843	0.778	0.836	0.987	0.666	0.85
A_84_P17436	At1g59530.1	1.401	1.289	1.483	1.995	1.687	1.763	2.36	0.577	0.634	0.596	0.644	0.61	0.463	0.648
A_84_P20982	At1g61950.1	1.3	1.202	1.382	1.499	1.092	2.555	1.3	0.598	0.633	0.608	0.665	0.689	0.509	0.53
A_84_P16139	At1g65670.1	1.318	1.731	1.14	0.776	1.964	2.235	3.699	0.857	0.684	0.902	1.09	0.838	0.635	0.838
A_84_P594345	At1g66030.1	2.106	2.132	1.794	1.787	1.46	2.118	1.396	0.655	0.734	0.661	0.776	0.761	0.684	0.645
A_84_P17981	At1g67270.1	1.505	1.162	1.242	1.852	1.035	1.477	1.87	0.667	0.517	0.498	0.545	0.787	0.77	0.603
A_84_P605032	At1g68935.1	0.828	1.181	0.956	1.241	1.437	1.114	1.242	1.075	0.971	0.809	0.672	0.674	1.084	0.928
A_84_P584570	At1g76210.1	1.491	1.703	2.126	1.185	1.757	2.74	1.173	0.909	0.84	0.755	0.823	0.791	0.73	0.665
A_84_P12413	At1g77730.1	1.193	1.078	1.279	1.298	1.478	1.263	0.777	0.912	0.813	0.693	0.855	0.519	0.377	0.511
A_84_P18234	At2g02310.1	1.849	2.493	1.847	1.424	1.516	1.951	1.326	0.745	0.775	0.601	0.818	0.825	0.726	0.65
A_84_P212758	At2g02620.1	1.803	1.627	1.706	2.171	1.726	1.685	1.344	0.596	0.624	0.596	0.695	0.63	0.525	0.532
A_84_P566753	At2g03830.1	1.427	1.383	1.051	1.685	1.433	1.277	1.507	0.735	0.613	0.614	0.701	0.706	0.573	0.578
A_84_P12272	At2g04025.1	1.134	1.116	1.048	1.434	1.176	1.221	1.29	0.751	0.696	0.456	0.66	1.539	0.736	0.661
A_84_P586382	At2g05330.1	1.138	1.091	0.964	1.484	1.366	1.358	0.847	1.13	0.804	0.932	0.701	0.89	0.581	0.58
A_84_P548152	At2g05400.1	1.464	1.699	1.971	1.417	2.175	2.427	2.821	1.11	0.788	0.766	0.815	0.784	0.658	0.738
A_84_P10618	At2g14920.1	1.644	1.63	1.469	2.512	1.266	1.743	1.337	0.608	0.729	0.688	0.672	0.786	0.75	0.637
A_84_P602427	At2g16575.1	1.821	2.214	1.805	1.892	2.37	2.201	1.849	0.516	0.516	0.545	0.542	0.521	0.696	0.436
A_84_P14147	At2g17050.1	1.593	0.954	1.029	0.931	1.989	1.552	2.497	0.716	0.779	0.847	0.969	1.065	0.979	0.866
A_84_P17307	At2g18640.1	1.478	1.427	1.586	1.402	1.303	2.724	1.173	0.685	0.721	0.782	0.753	0.81	0.586	0.584
A_84_P153835	At2g21650.1	1.46	1.567	1.92	1.416	1.869	1.082	1.24	0.562	1.238	0.953	1.013	0.946	0.589	0.476
A_84_P21025	At2g22290.1	1.266	1.299	1.332	1.504	1.294	2.145	1.26	0.638	0.542	0.573	0.615	0.604	0.478	0.54
A_84_P554387	At2g23440.1	1.521	1.26	1.536	0.894	1.232	2.871	2.541	0.84	0.882	0.772	0.94	0.908	0.694	0.857
A_84_P22001	At2g24710.1	2.604	1.616	1.327	1.593	1.468	3.848	3.47	0.821	0.855	0.814	0.9	0.997	0.903	0.733
A_84_P11673	At2g27440.1	1.187	1.317	1.32	1.633	1.331	1.536	1.373	0.628	0.602	0.577	0.636	0.616	0.508	0.49
A_84_P170463	At2g31082.1	1.288	1.237	1.21	1.726	1.287	1.538	1.312	0.758	0.623	0.598	0.759	0.752	0.682	0.702
A_84_P295664	At2g31083.1	1.425	1.35	1.308	1.371	1.228	2.053	1.014	0.674	0.656	0.701	0.755	0.706	0.676	0.654
A_84_P525165	At2g31480.1	1.221	1.195	1.62	1.302	1.271	1.514	1.336	0.584	0.641	0.66	0.65	0.858	0.712	0.579
A_84_P140119	At2g34020.1	1.378	1.277	1.274	2.321	1.498	1.251	1.119	0.968	0.718	0.723	1.031	0.66	0.835	0.617
A_84_P588220	At2g34330.1	1.572	2.315	1.784	1.756	1.531	2.431	1.964	0.707	0.74	0.663	0.725	0.691	0.559	0.59
A_84_P542928	At2g40450.1	1.512	1.296	0.966	2.169	1.357	1.376	1.51	0.648	0.551	0.631	0.733	0.852	0.676	0.576
A_84_P139609	At2g44380.1	1.82	2.129	1.761	1.149	1.81	2.274	1.069	0.766	0.805	0.779	0.84	0.803	0.649	0.685
A_84_P12625	At3g03800.1	1.231	1.49	1.418	1.756	2.01	2.277	1.681	0.778	0.646	0.693	0.633	0.697	0.749	0.793
A_84_P257110	At3g04420.1	1.458	1.556	1.097	1.18	1.257	1.866	2.138	0.615	0.57	0.538	0.667	0.883	0.672	0.665
A_84_P19270	At3g05930.1	2.064	2.729	2.191	2.159	1.326	2.335	1.786	0.752	0.794	0.749	0.817	0.786	0.641	0.664
A_84_P242653	At3g06990.1	1.057	0.987	0.999	1.24	1.041	1.105	1.097	0.891	0.957	0.938	0.655	1.111	1.145	1.13
A_84_P248505	At3g07620.1	1.766	2.078	1.581	2.586	1.451	1.611	1.226	0.756	0.772	0.854	0.779	0.588	0.508	0.694
A_84_P504860	At3g09280.1	1.183	1.148	1.326	1.516	1.635	1.227	1.985	0.761	0.851	0.667	0.622	0.746	0.796	0.732
A_84_P21241	At3g12220.1	1.71	0.903	1.57	2.107	1.656	1.356	1.321	0.621	0.654	0.629	0.686	0.651	0.559	0.599
A_84_P128101	At3g15830.1	1.504	1.619	1.734	1.021	1.715	1.578	1.203	0.664	0.673	0.532	0.999	0.957	0.647	0.63
A_84_P17417	At3g18460.1	2.074	1.728	1.886	1.612	1.814	2.038	1.062	0.721	0.702	0.747	0.813	0.843	0.635	0.666
A_84_P24018	At3g23360.1	1.387	1.923	1.491	2.781	1.308	1.964	1.329	0.739	0.663	0.63	0.698	0.671	0.536	0.56

p value

		ROOTS							SHOOTS						
		EARLY			MIDDLE		LATE		EARLY			MIDDLE		LATE	
Gene Code	Gene model	10m	1h	2h	4h	24h	48	96h	10m	1h	2h	4h	24h	48	96h
A_84_P572778	At1g02040.1	0.0163	0.0216	0.0158	0.0125	0.212	0.259	0.089	0.423	0.581	0.424	0.49	0.386	0.41	0.212
A_84_P18004	At1g04670.1	0.0372	0.0319	0.0627	0.129	0.147	0.0273	0.0273	0.493	0.681	0.63	0.73	0.684	0.494	0.522
A_84_P21815	At1g05577.1	0.163	0.162	0.135	0.0318	0.0434	0.622	0.0672	0.259	0.551	0.421	0.353	0.217	0.551	0.311
A_84_P17085	At1g05650.1	0.0499	0.0459	0.0186	0.0239	0.85	0.0222	0.789	0.622	0.419	0.394	0.527	0.444	0.738	0.471
A_84_P10574	At1g06990.1	0.0403	0.0199	0.105	0.0101	0.518	0.0326	0.0095	0.877	0.668	0.618	0.943	0.686	0.47	0.512
A_84_P262310	At1g10880.1	0.00383	0.0925	0.0474	0.0318	0.037	0.167	0.0359	0.97	0.814	0.771	0.887	0.56	0.58	0.636
A_84_P20852	At1g11920.1	0.022	0.0248	0.0252	0.00718	0.454	0.75	0.792	0.715	0.819	0.772	0.883	0.875	0.598	0.648
A_84_P18909	At1g13310.1	0.00348	0.0242	0.363	0.0268	0.0386	0.00333	0.0376	0.399	0.639	0.459	0.538	0.5	0.299	0.89
A_84_P582608	At1g13540.1	0.0118	0.0491	0.0259	0.0338	0.0243	0.0201	0.0994	0.718	0.614	0.565	0.666	0.607	0.427	0.549
A_84_P18086	At1g18410.1	0.0214	0.354	0.0225	0.00543	0.0473	0.01	0.0121	0.407	0.461	0.541	0.656	0.665	0.389	0.682
A_84_P14044	At1g29540.1	0.412	0.923	0.202	0.344	0.381	0.0172	0.0301	0.679	0.494	0.68	0.196	0.986	0.805	0.979
A_84_P15241	At1g33890.1	0.00491	0.0375	0.0349	0.124	0.0698	0.416	0.121	0.451	0.883	0.546	0.708	0.67	0.322	0.498
A_84_P10830	At1g34540.1	0.252	0.0661	0.0101	0.0361	0.951	0.00252	0.0461	0.364	0.608	0.668	0.567	0.367	0.643	0.467
A_84_P285470	At1g44030.1	0.000912	0.0507	0.0667	0.0439	0.0255	0.00584	0.813	0.324	0.438	0.43	0.461	0.569	0.354	0.319
A_84_P541828	At1g48070.1	0.00404	0.0203	0.0277	0.023	0.0833	0.00562	0.561	0.652	0.511	0.661	0.827	0.668	0.556	0.464
A_84_P13245	At1g51220.1	0.0123	0.0426	0.176	0.0199	0.0213	0.16	0.0693	0.955	0.851	0.804	0.728	0.812	0.713	0.578
A_84_P521460	At1g52240.1	0.00114	0.00986	0.00565	0.000662	0.351	0.00283	0.747	0.526	0.659	0.614	0.738	0.741	0.523	0.298
A_84_P19775	At1g55230.1	0.0616	0.385	0.126	0.0191	0.0149	0.0241	0.715	0.349	0.312	0.362	0.399	0.657	0.724	0.662
A_84_P605885	At1g55990.1	0.00385	0.00627	0.0106	0.0505	0.892	0.0395	0.626	0.502	0.312	0.565	0.918	0.545	0.503	0.465
A_84_P14049	At1g56160.1	0.00896	0.000302	0.0469	0.0715	0.658	0.0803	0.632	0.665	0.849	0.599	0.712	0.526	0.4	0.467
A_84_P572925	At1g58643.1	0.0832	0.846	0.721	0.422	0.0909	0.00901	0.0137	0.636	0.786	0.698	0.776	0.983	0.547	0.794
A_84_P17436	At1g59530.1	0.0142	0.153	0.0376	0.0154	0.0637	0.00928	0.00689	0.299	0.483	0.41	0.499	0.428	0.269	0.487
A_84_P20982	At1g61950.1	0.00549	0.0938	0.0167	0.0095	0.68	0.0131	0.0497	0.344	0.487	0.456	0.53	0.559	0.339	0.364
A_84_P16139	At1g65670.1	0.483	0.328	0.763	0.633	0.0639	0.0123	0.0088	0.761	0.526	0.871	0.882	0.773	0.493	0.764
A_84_P594345	At1g66030.1	0.00000421	0.0184	0.0122	0.0071	0.298	0.0382	0.491	0.418	0.632	0.521	0.689	0.669	0.559	0.512
A_84_P17981	At1g67270.1	0.0952	0.153	0.0459	0.0367	0.837	0.00536	0.0342	0.442	0.333	0.313	0.366	0.681	0.647	0.424
A_84_P605032	At1g68935.1	0.433	0.555	0.9	0.504	0.331	0.763	0.529	0.61	0.846	0.138	0.0349	0.0129	0.443	0.545
A_84_P584570	At1g76210.1	0.0258	0.00978	0.000149	0.432	0.0843	0.00996	0.772	0.845	0.78	0.664	0.758	0.715	0.625	0.544
A_84_P12413	At1g77730.1	0.0439	0.392	0.0387	0.0368	0.00912	0.0476	0.157	0.824	0.712	0.492	0.771	0.321	0.19	0.307
A_84_P18234	At2g02310.1	0.00128	0.00948	0.0498	0.0457	0.0114	0.015	0.0743	0.575	0.691	0.419	0.752	0.761	0.619	0.508
A_84_P212758	At2g02620.1	0.000117	0.00656	0.00189	0.0019	0.00311	0.00386	0.277	0.338	0.473	0.438	0.566	0.482	0.352	0.363
A_84_P566753	At2g03830.1	0.00718	0.0229	0.72	0.00282	0.0121	0.0925	0.00719	0.512	0.449	0.449	0.565	0.573	0.399	0.403
A_84_P12272	At2g04025.1	0.093	0.31	0.654	0.00712	0.107	0.0457	0.023	0.511	0.546	0.223	0.502	0.35	0.331	0.355
A_84_P586382	At2g05330.1	0.186	0.448	0.683	0.0102	0.0186	0.0159	0.611	0.704	0.633	0.873	0.419	0.803	0.327	0.328
A_84_P548152	At2g05400.1	0.0583	0.167	0.0187	0.0965	0.0335	0.0117	0.00439	0.822	0.709	0.678	0.748	0.704	0.533	0.637
A_84_P10618	At2g14920.1	0.000377	0.03	0.0233	0.0005	0.126	0.000927	0.155	0.357	0.611	0.556	0.538	0.693	0.633	0.487
A_84_P602427	At2g16575.1	0.0223	0.0422	0.0541	0.0833	0.0294	0.0149	0.0377	0.235	0.328	0.356	0.359	0.341	0.51	0.249
A_84_P14147	At2g17050.1	0.239	0.925	0.954	0.861	0.000801	0.00216	0.0404	0.515	0.654	0.753	0.954	0.916	0.964	0.765
A_84_P17307	At2g18640.1	0.000599	0.0177	0.00642	0.012	0.187	0.00523	0.301	0.477	0.612	0.694	0.657	0.735	0.437	0.423
A_84_P153835	At2g21650.1	0.193	0.177	0.0662	0.214	0.0539	0.719	0.377	0.0002	0.459	0.879	0.97	0.824	0.0057	0.0204
A_84_P21025	At2g22290.1	0.00936	0.0299	0.0207	0.00352	0.149	0.0418	0.169	0.38	0.339	0.405	0.458	0.444	0.298	0.365
A_84_P554387	At2g23440.1	0.233	0.653	0.277	0.847	0.672	0.00783	0.0136	0.74	0.842	0.681	0.922	0.881	0.585	0.809
A_84_P22001	At2g24710.1	0.0148	0.36	0.538	0.343	0.421	0.00807	0.00966	0.711	0.806	0.749	0.867	0.997	0.869	0.639
A_84_P11673	At2g27440.1	0.113	0.0305	0.0295	0.0105	0.0557	0.0109	0.0187	0.376	0.442	0.412	0.486	0.462	0.331	0.292
A_84_P170463	At2g31082.1	0.00589	0.0613	0.075	0.0009	0.062	0.00236	0.0193	0.574	0.473	0.443	0.653	0.643	0.542	0.571
A_84_P295664	At2g31083.1	0.00191	0.0219	0.0366	0.13	0.431	0.0171	0.975	0.449	0.519	0.575	0.652	0.584	0.536	0.511
A_84_P525165	At2g31480.1	0.0612	0.168	0.0109	0.0617	0.0728	0.0173	0.0376	0.293	0.49	0.526	0.496	0.788	0.588	0.425
A_84_P140119	At2g34020.1	0.0481	0.37	0.174	0.00941	0.0493	0.135	0.55	0.947	0.55	0.58	0.949	0.501	0.753	0.441
A_84_P588220	At2g34330.1	0.000697	0.0301	0.00651	0.00304	0.23	0.0133	0.00825	0.505	0.634	0.532	0.617	0.571	0.402	0.44
A_84_P542928	At2g40450.1	0.0158	0.193	0.887	0.0162	0.146	0.0226	0.00448	0.401	0.359	0.474	0.612	0.782	0.519	0.374
A_84_P139609	At2g44380.1	0.00257	0.00575	0.0139	0.388	0.0745	0.0318	0.88	0.613	0.734	0.698	0.784	0.733	0.524	0.572
A_84_P12625	At3g03800.1	0.129	0.0423	0.0652	0.0385	0.0488	0.0185	0.17	0.602	0.497	0.556	0.474	0.565	0.627	0.692
A_84_P257110	At3g04420.1	0.0965	0.145	0.726	0.449	0.214	0.0154	0.00498	0.32	0.385	0.288	0.533	0.83	0.497	0.523
A_84_P19270	At3g05930.1	0.0133	0.0194	0.0412	0.052	0.372	0.0326	0.0439	0.587	0.718	0.656	0.75	0.708	0.511	0.527
A_84_P242653	At3g06990.1	0.444	0.882	0.994	0.0427	0.736	0.234	0.445	0.312	0.693	0.45	0.0111	0.468	0.198	0.385
A_84_P248505	At3g07620.1	0.0252	0.0208	0.0146	0.0157	0.347	0.306	0.659	0.587	0.658	0.797	0.695	0.4	0.32	0.577
A_84_P504860	At3g09280.1	0.153	0.329	0.0842	0.00896	0.00351	0.0824	0.0023	0.58	0.779	0.521	0.427	0.629	0.684	0.603
A_84_P21241	At3g12220.1	0.000877	0.821	0.0423	0.00406	0.00353	0.0292	0.0621	0.376	0.516	0.484	0.559	0.514	0.395	0.444
A_84_P128101	At3g15830.1	0.00525	0.0104	0.00728	0.879	0.032	0.0243	0.326	0.427	0.514	0.284	0.999	0.933	0.512	0.45
A_84_P17417	At3g18460.1	0.0015	0.0141	0.00711	0.00624	0.169	0.0678	0.885	0.509	0.571	0.654	0.744	0.785	0.505	0.545
A_84_P24018	At3g23360.1	0.00318	0.00353	0.0245	0.00395	0.0738	0.0011	0.445	0.554	0.527	0.486	0.576	0.542	0.371	0.4

A_84_P252155	At3g28650.1	1.631	1.413	1.503	1.461	1.45	1.202	1.452	0.653	0.557	0.648	0.716	0.683	0.499	0.698	0.00323	0.116	0.0373	0.0277	0.0327	0.11	0.0118	0.399	0.366	0.51	0.602	0.537	0.242	0.519
A_84_P592843	At3g29580.1	2.191	1.841	1.887	1.833	2.635	1.987	2.08	0.741	0.755	0.755	0.788	0.814	0.621	0.767	0.0289	0.134	0.116	0.155	0.0627	0.0271	0.032	0.547	0.662	0.66	0.708	0.743	0.445	0.673
A_84_P74604	At3g45530.1	1.574	1.586	1.259	1.523	1.49	1.725	1.607	0.69	0.67	0.724	0.746	0.632	0.421	0.438	0.00439	0.041	0.181	0.0522	0.056	0.0254	0.0476	0.424	0.493	0.584	0.602	0.463	0.236	0.253
A_84_P14634	At3g46190.1	1.744	2.034	2.037	1.498	1.576	0.69	2.161	1.123	0.916	0.911	0.666	0.932	0.715	0.998	0.0247	0.0366	0.0461	0.392	0.0104	0.251	0.264	0.803	0.878	0.855	0.498	0.897	0.543	0.997
A_84_P10854	At3g46330.1	2.19	1.47	1.463	1.872	1.992	1.802	1.373	0.803	0.823	0.765	0.87	1.014	0.904	0.674	0.0307	0.27	0.337	0.157	0.00129	0.00137	0.0101	0.675	0.76	0.658	0.826	0.982	0.869	0.533
A_84_P167283	At3g46800.1	1.722	1.415	1.774	1.215	1.244	2.287	1.47	0.663	0.609	0.611	0.642	0.646	0.754	0.67	0.00298	0.109	0.0374	0.235	0.185	0.000726	0.0207	0.422	0.454	0.448	0.497	0.502	0.607	0.526
A_84_P117432	At3g46810.1	1.79	1.833	1.509	1.299	1.809	1.675	1.43	0.633	0.649	0.622	0.683	0.668	0.64	0.597	0.000299	0.00552	0.0278	0.0335	0.00828	0.000899	0.0061	0.391	0.509	0.474	0.555	0.534	0.49	0.44
A_84_P288500	At3g47710.1	1.222	1.04	1.153	1.66	1.515	1.43	1.182	0.625	0.823	0.535	0.67	0.972	1.04	0.354	0.0201	0.667	0.186	0.00341	0.0435	0.00844	0.147	0.308	0.735	0.313	0.522	0.959	0.936	0.11
A_84_P14651	At3g50150.1	1.438	1.333	1.287	1.843	1.316	1.692	0.972	0.597	0.639	0.537	0.648	0.725	0.763	0.538	0.000908	0.0303	0.0437	0.000995	0.0514	0.00527	0.825	0.328	0.481	0.361	0.495	0.596	0.644	0.358
A_84_P23179	At3g53600.1	1.416	1.342	1.565	1.477	1.859	1.97	1.785	0.698	0.716	0.683	0.762	0.717	0.705	0.756	0.00325	0.0419	0.00922	0.0161	0.0202	0.0348	0.0189	0.495	0.604	0.562	0.669	0.609	0.586	0.656
A_84_P549304	At3g54530.1	1.104	1.065	1.365	1.681	1.612	1.622	1.839	0.762	0.589	0.67	0.613	0.588	0.5	0.662	0.167	0.469	0.0083	0.00523	0.0046	0.00596	0.0921	0.573	0.425	0.523	0.456	0.426	0.32	0.491
A_84_P16565	At3g55180.1	1.289	1.383	1.306	1.653	1.256	2.053	1.082	0.565	0.512	0.665	0.601	1	0.731	0.859	0.0138	0.0197	0.0381	0.00299	0.0795	0.0114	0.839	0.24	0.313	0.505	0.436	0.999	0.549	0.788
A_84_P556400	At3g58210.1	2.769	2.026	1.85	2.497	1.478	2.392	2.887	0.786	0.812	0.78	0.862	0.827	0.695	0.693	0.00762	0.174	0.204	0.0508	0.445	0.0168	0.0337	0.645	0.745	0.7	0.814	0.766	0.584	0.584
A_84_P539588	At3g61340.1	2.425	1.557	1.517	2.095	1.496	1.71	2.552	0.56	0.604	0.586	0.639	0.526	0.509	0.543	0.00161	0.0956	0.191	0.0216	0.122	0.0113	0.00157	0.271	0.425	0.402	0.442	0.34	0.313	0.351
A_84_P15658	At4g00540.1	1.568	1.652	1.372	1.169	1.219	2.771	2.693	0.734	0.619	0.691	0.677	0.716	0.791	0.668	0.142	0.294	0.518	0.757	0.529	0.0227	0.0328	0.49	0.455	0.549	0.531	0.557	0.683	0.498
A_84_P118332	At4g01265.1	2.252	1.471	1.084	2.805	1.989	1.686	1.915	0.63	0.672	0.731	0.786	0.79	0.607	0.794	0.0582	0.428	0.774	0.022	0.0782	0.019	0.0215	0.424	0.53	0.628	0.705	0.71	0.465	0.71
A_84_P23229	At4g01520.1	1.196	0.816	1.608	0.798	1.677	2.98	2.818	0.769	0.691	1.105	0.956	0.917	1.074	0.797	0.551	0.653	0.25	0.649	0.115	0.00574	0.018	0.611	0.514	0.862	0.939	0.871	0.889	0.696
A_84_P23230	At4g01760.1	1.131	1.469	1.578	1.515	1.375	1.309	1.344	0.756	0.595	0.572	0.669	0.619	0.592	0.684	0.371	0.114	0.0194	0.016	0.0357	0.0262	0.0161	0.57	0.434	0.406	0.527	0.437	0.352	0.539
A_84_P547576	At4g03480.1	1.686	2.043	1.661	1.373	1.591	2.316	0.98	0.682	0.711	0.822	0.749	0.729	0.587	0.634	0.00153	0.0179	0.0414	0.055	0.217	0.00727	0.97	0.474	0.6	0.75	0.653	0.626	0.439	0.497
A_84_P18512	At4g04930.1	1.583	1.698	1.414	2.28	1.502	1.77	1.274	0.697	0.74	0.667	0.733	0.756	0.701	0.772	0.000785	0.00975	0.04	0.000545	0.0322	0.00892	0.169	0.492	0.635	0.539	0.628	0.66	0.578	0.677
A_84_P14736	At4g09120.1	1.455	1.441	1.721	1.997	1.935	2.13	1.45	0.654	0.704	0.675	0.756	0.824	0.643	1.043	0.0121	0.0195	0.00326	0.000571	0.0116	0.0144	0.0371	0.405	0.588	0.549	0.66	0.755	0.502	0.94
A_84_P10013	At4g09940.1	1.509	1.972	1.748	2.052	1.419	1.538	1.414	0.796	0.641	0.644	0.781	0.856	0.564	0.689	0.00479	0.00123	0.00837	0.00153	0.0122	0.00295	0.0204	0.652	0.48	0.479	0.698	0.8	0.4	0.565
A_84_P13796	At4g10510.1	1.292	1.411	1.351	1.027	1.328	1.368	1.352	0.707	0.535	1.01	0.703	0.733	0.54	0.631	0.206	0.0364	0.0732	0.919	0.106	0.0247	0.0486	0.501	0.316	0.986	0.573	0.587	0.33	0.469
A_84_P10965	At4g11390.1	2.144	1.872	1.613	2.337	1.48	2.28	1.807	1.04	0.854	0.825	0.907	0.755	0.755	0.744	0.00801	0.0977	0.235	0.0326	0.329	0.0218	0.0375	0.935	0.804	0.766	0.877	0.65	0.667	0.655
A_84_P13904	At4g14370.1	1.765	1.864	2.271	2.182	3.624	1.288	1.263	0.677	0.768	0.889	0.595	0.598	0.568	0.69	0.104	0.157	0.0563	0.036	0.00661	0.361	0.37	0.431	0.652	0.832	0.426	0.445	0.394	0.547
A_84_P519221	At4g14860.1	1.12	1.089	1.311	1.232	1.266	1.658	1.747	0.844	0.567	0.593	0.697	0.959	0.535	0.571	0.176	0.351	0.0207	0.0634	0.14	0.0163	0.00307	0.691	0.362	0.365	0.561	0.937	0.305	0.353
A_84_P528108	At4g15150.1	1.079	1.146	1.085	1.192	0.966	1.026	1.224	0.865	0.791	0.959	0.974	0.839	0.914	0.709	0.319	0.133	0.316	0.171	0.672	0.753	0.0464	0.368	0.19	0.841	0.878	0.356	0.482	0.0436
A_84_P18636	At4g15350.1	1.528	1.267	1.445	1.881	1.229	1.516	2.087	0.646	0.664	0.499	0.763	0.714	0.649	0.581	0.00106	0.0888	0.0158	0.0113	0.15	0.00249	0.000885	0.373	0.526	0.287	0.655	0.573	0.429	0.384
A_84_P572459	At4g18335.1	2.154	2.002	1.533	2.741	2.411	2.377	3.568	0.774	0.711	0.652	0.714	0.68	0.621	0.582	0.0105	0.0992	0.172	0.0232	0.0265	0.0152	0.00834	0.609	0.594	0.517	0.6	0.556	0.473	0.429
A_84_P158165	At4g19690.1	2.037	1.688	1.537	1.552	1.484	2.506	1.11	0.803	0.843	0.789	0.886	0.852	0.685	0.718	0.0141	0.000579	0.0198	0.0252	0.28	0.0482	0.84	0.676	0.789	0.705	0.847	0.802	0.574	0.618
A_84_P22364	At4g27550.1	1.372	1.404	1.297	2.837	1.173	1.328	1.731	0.655	0.557	0.565	0.688	0.492	0.679	1.046	0.111	0.19	0.41	0.00781	0.421	0.0454	0.00327	0.406	0.372	0.395	0.551	0.293	0.522	0.927
A_84_P14801	At4g28620.1	2.184	1.108	1.159	1.673	2.296	1.661	2.29	0.914	0.814	0.678	0.858	0.954	0.858	0.749	0.0348	0.839	0.726	0.275	0.0223	0.0295	0.00571	0.852	0.747	0.535	0.809	0.938	0.804	0.645
A_84_P12925	At4g31710.1	1.586	2.181	1.464	1.68	2.128	1.162	1.286	0.696	0.632	0.793	0.869	0.81	0.685	0.566	0.167	0.0125	0.0715	0.0441	0.0139	0.694	0.476	0.481	0.45	0.713	0.824	0.745	0.544	0.385
A_84_P501542	At4g33590.1	1.766	1.564	1.493	3.062	1.488	1.803	1.469	0.721	1.007	0.717	0.747	0.756	0.584	0.547	0.0072	0.0277	0.0256	0.000576	0.0833	0.00517	0.185	0.54	0.991	0.609	0.64	0.667	0.431	0.375
A_84_P185584	At4g39590.1	1.387	1.227	1.136	1.908	1.275	1.384	1.372	0.674	0.599	0.514	0.665	0.933	0.882	0.611	0.087	0.0941	0.415	0.00583	0.104	0.0124	0.0131	0.432	0.432	0.319	0.517	0.903	0.818	0.429
A_84_P12998	At5g07450.1	1.262	1.23	1.363	1.432	1.844	1.812	1.132	0.705	0.54	0.658	0.641	0.61	0.454	0.683	0.0149	0.0633	0.0408	0.00849	0.0208	0.0404	0.469	0.461	0.359	0.498	0.48	0.435	0.268	0.528
A_84_P23402	At5g07550.1	1.483	1.183	1.173	1.558	1.584	1.393	1.649	0.702	0.713	0.684	0.702	0.966	0.602	0.695	0.00447	0.224	0.275	0.0324	0.0077	0.02	0.00567	0.421	0.551	0.532	0.535	0.95	0.344	0.522
A_84_P56160	At5g07780.1	1.734	1.811	1.346	1.993	1.244	2.288	1.48	0.709	0.737	0.843	0.777	0.743	0.593	0.62	0.00107	0.032	0.161	0.00752	0.528	0.00949	0.0402	0.521	0.635	0.779	0.691	0.649	0.447	0.482
A_84_P548604	At5g09720.1	2.676	1.632	1.298	1.768	1.628	1.551	2.076	0.736	0.79	0.736	0.81	0.778	0.684	0.657	0.00403	0.23	0.537	0.0182	0.0152	0.0144	0.00158	0.561	0.71	0.636	0.739	0.696	0.563	0.532
A_84_P23409	At5g10130.1	1.09	1.135	1.078	1.39	0.911	1.364	1.085	0.851	0.68	0.859	0.624	1.076	0.682	0.56	0.344	0.148	0.368	0.046	0.3	0.0395	0.719	0.362	0.0869	0.388	0.0884	0.814	0.157	0.0384
A_84_P506930	At5g10680.1	1.513	1.386	1.111	2.388	1.676	1.399	2.127	0.593	0.678	0.658	0.609	0.745	0.815	0.62	0.169	0.316	0.754	0.0344	0.0418	0.078	0.0753	0.325	0.512	0.505	0.448	0.621	0.716	0.454
A_84_P583283	At5g16330.1	2.545	2.337	2.049	2.686	1.34	1.489	2.226	0.793	0.793	0.756	0.834	0.799	0.635	0.668	0.0042	0.0085	0.017	0.00315	0.572	0.397	0.128	0.659	0.717	0.665	0.774	0.73	0.505	0.548
A_84_P542422	At5g17340.1	1.274	1.233	1.129	1.914	2.339	1.419	1.388	0.663	0.579	0.532	0.566	0.781	0.751	0.899	0.0508	0.154	0.405	0.00594	0.0387	0.0563	0.0579	0.41	0.404	0.35	0.392	0.67	0.617	0.843
A_84_P18861	At5g20470.1	2.466	1.758	1.055	1.236	2.088	1.744	1.457	0.82	0.866	0.833	0.998	0.91	0.696	0.955	0.0104	0.245	0.898	0.432	0.000362	0.00455	0.0144	0.706	0.821	0.777	0.998	0.882	0.592	0.939
A_84_P21552	At5g24880.1	1.245	1.506	1.438	1.283	1.118	1.518	0.981	0.645	0.499	0.507	0.557	0.88	0.437	0.517	0.00878	0.00455	0.00693	0.0259	0.394	0.00309	0.865	0.333	0.278	0.323	0.38	0.811	0.215	0.272
A_84_P552182	At5g25750.1	1.826	2.425	2.021	1.462	1.937	2.218	3.822	0.72	0.692	0.665	0.735	0.705	0.695	0.722	0.0573	0.0699	0.108	0.331	0.168	0.0251	0.00784	0.525	0.571	0.534	0.629	0.59	0.568	0.606
A_84_P583317	At5g26320.1	1.566	1.577	1.725	1.197	1.124	2.367	1.274	0.748	0.749	0.707	0.885	0.854	0.539	0.516	0.00301	0.0175	0.00373	0.339	0.773	0.00828	0.144	0.542	0.649	0.586	0.838	0.796	0.362	0.319
A_84_P591494	At5g27810.1	2.045	2.907	2.599	2.481	2.46	2.437	1.807	0.734	0.772	0.738	0.806	0.776	0.752	0.778	0.0116	0.00416	0.016	0.0107	0.0412	0.015	0.121	0.558	0.687	0.64	0.734	0.693	0.653	0.691
A_84_P603820	At5g38320.1	1.091	1.083	1.037	1.226	1.271	1.102	1.043	0.836	1.06	1.124	0.973	1.095	0.71	0.661	0.637	0.704	0.87	0.469	0.294	0.697	0.826	0.419	0.861	0.625	0.91	0.681	0.0192	0.0362
A_84_P22525	At5g39930.1	1.422	1.738	0.987	1.77	1.308	1.911	2.792	0.71	0.746	0.686	0.784	0.754	0.562	0.784	0.0644	0.183	0.974	0.11	0.254	0.0142	0.00572	0.519	0.649	0.561	0.702	0.662	0.394	0.665
A_84_P612712	At5g41300.1	2.051	1.93	1.413	1.787	1.712	1.363	1.334	0.664	0.706	0.684	0.719	0.855	0.691	0.673	0.0025	0.00148	0.188	0.0314	0.0233	0.0188	0.0415	0.444	0.566	0.552	0.578	0.791	0.563	0.517
A_84_P533752	At5g41380.1	2.112	1.862	1.625	1.838	1.968	2.925	2.717	0.786	0.796	0.792	0.859	0.854	0.671	0.706	0.0681	0.233	0.316	0.23	0.169	0.0383	0.036	0.647	0.719	0.718	0.81	0.803	0.555	0.602
A_84_P566529	At5g41730.1	1.418	1.791	1.928	1.807	1.754	2.468	1.289	0.787	0.836	0.803	0.876	0.819	0.67	0.577	0.0108	0.00773	0.00392	0.00532	0.0437	0.00584	0.132	0.649	0.779	0.734	0.835	0.758	0.556	0.4
A_84_P19710	At5g46040.1	2.028	1.899	1.989	1.947	1.626	3.169	1.499	0.726	0.743	0.71	0.784	0.757	0.604	0.629	0.000011	0.00359	0.00188	0.00161	0.198	0.00432	0.317	0.521	0.645	0.6	0.702	0.666	0.462	0.495
A_84_P12154	At5g48130.1	1.35	2.909	1.901	2.995	1.747	1.713	1.809	0.511	0.533	0.512	0.562	0.555	0.555	0.52	0.0723	0.0141	0.0671	0.00322	0.0493	0.0234	0.0218	0.229	0.352	0.329	0.386	0.378	0.371	0.334
A_84_P523896	At5g50335.1	1.025	1	0.941	1.046	1.247	1.449	2.058	0.863	0.87	0.988	1.102	1.004	0.88	1.208	0.913	0.996	0.689	0.921	0.47	0.02	0.00717	0.363	0.137	0.93	0.465	0.968	0.157	0.0594
A_84_P510592	At5g52680.1	1.499	1.162	1.583	1.167	2.306	2.261	3.188	0.935	0.663	0.731	0.662	0.903	0.858	0.837	0.285	0.79	0.403	0.761	0.0449	0.0452	0.0112	0.882	0.523	0.612	0.524	0.852	0.783	0.753
A_84_P15014	At5g55360.1	1.557	1.582	1.506	1.424	1.574	2.37	1.242	0.624	0.766	0.67	0.837	0.613	0.492	0.516	0.022	0.03	0.0411	0.119	0.091	0.00772	0.361	0.368	0.657	0.526	0.752	0.459	0.315	0.342
A_84_P570015	At5g57123.1	0.959	1.088	1.044	1.172	1.069	1.114	1.152	0.894	1.209	0.903	0.999	1.054	0.775	0.701	0.561	0.356	0.624	0.128	0.494	0.342	0.176	0.634	0.559	0.761	0.998	0.886	0.0464	0.0154
A_84_P535453	At5g60350.1	1.296	1.189	1.417	1.662	1.489	1.531	3.042	0.672	0.64	0.627	0.612	0.709	0.468	0.578	0.0076	0.12	0.0113	0.0938	0.0109	0.0272	2.12E-05	0.433	0.468	0.451	0.452	0.567	0.288	0.403
A_84_P15988	At5g62110.1	1.61	1.029	0.67	1.808	1.639	2.73	4.473	0.906	0.906	0.868	0.961	0.917	0.742	0.774	0.237	0.963	0.497	0.274	0.257	0.0206	0.00214	0.847	0.876	0.825	0.949	0.892	0.655	0.698
A_84_P11265	At5g62750.1	1.447	1.299	1.635	1.721	2.053	1.515	1.575	0.854	0.885	0.552	0.712	0.711	0.508	0.765	0.161	0.223	0.0675	0.135	0.00536	0.0179	0.0176	0.753	0.83	0.36	0.585	0.595	0.294	0.681

SUPPLEMENTARY DATA SET S2: Functional characterization

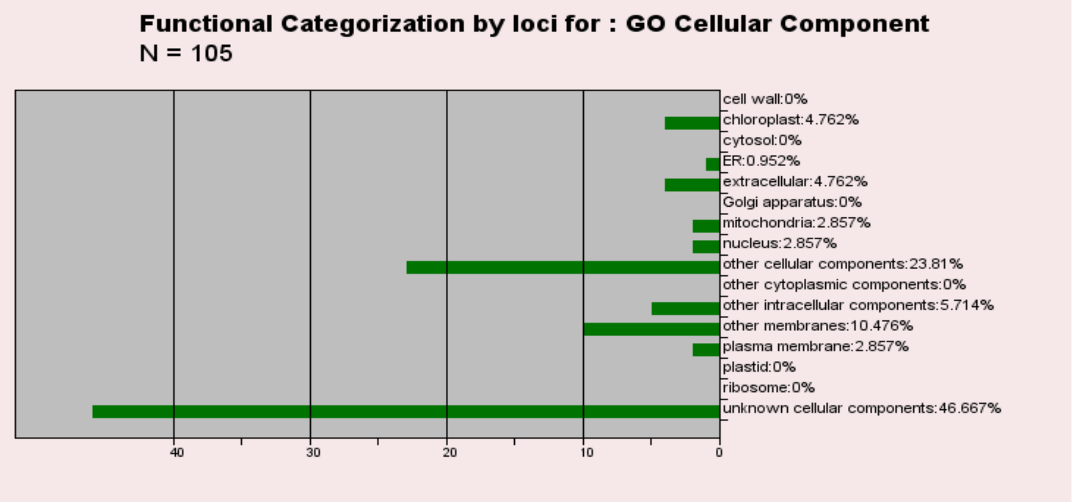

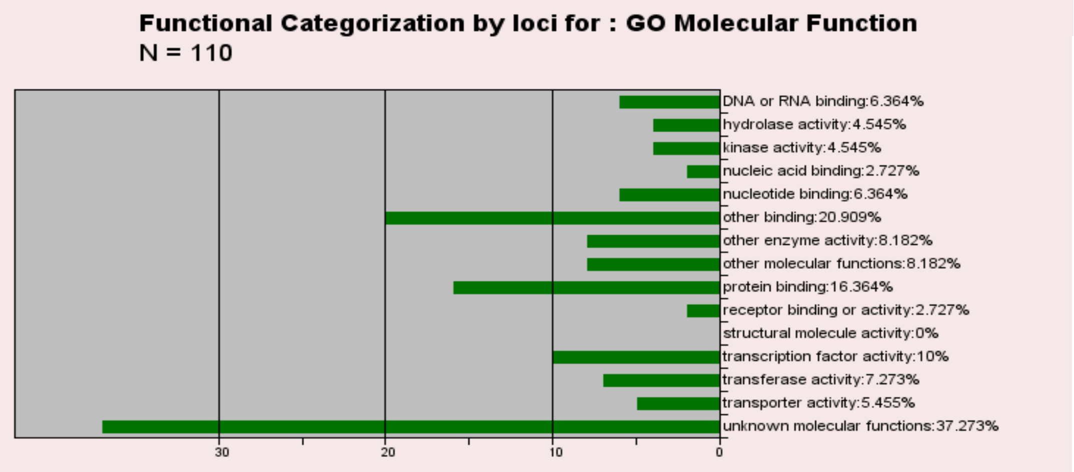

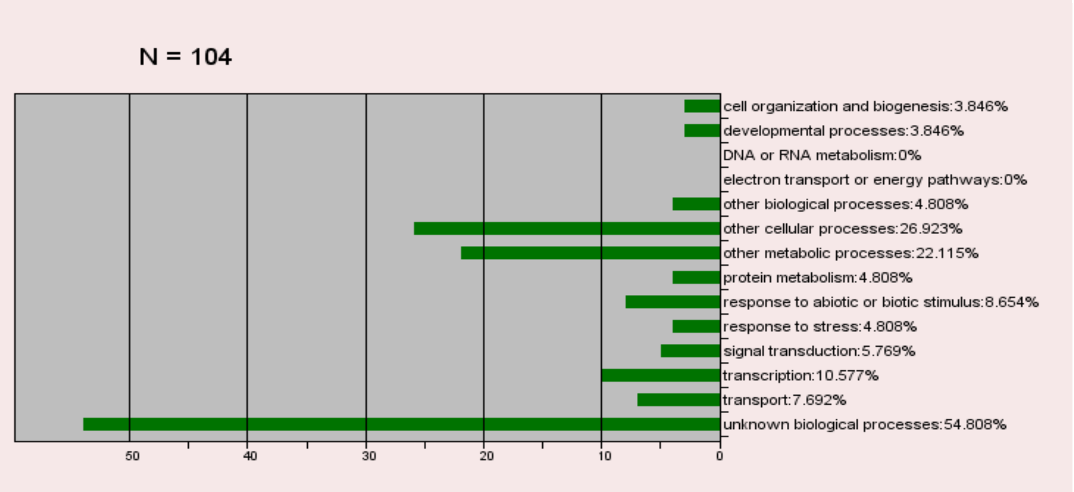

SUPPLEMENTARY DATA SET S2: GO Analysis

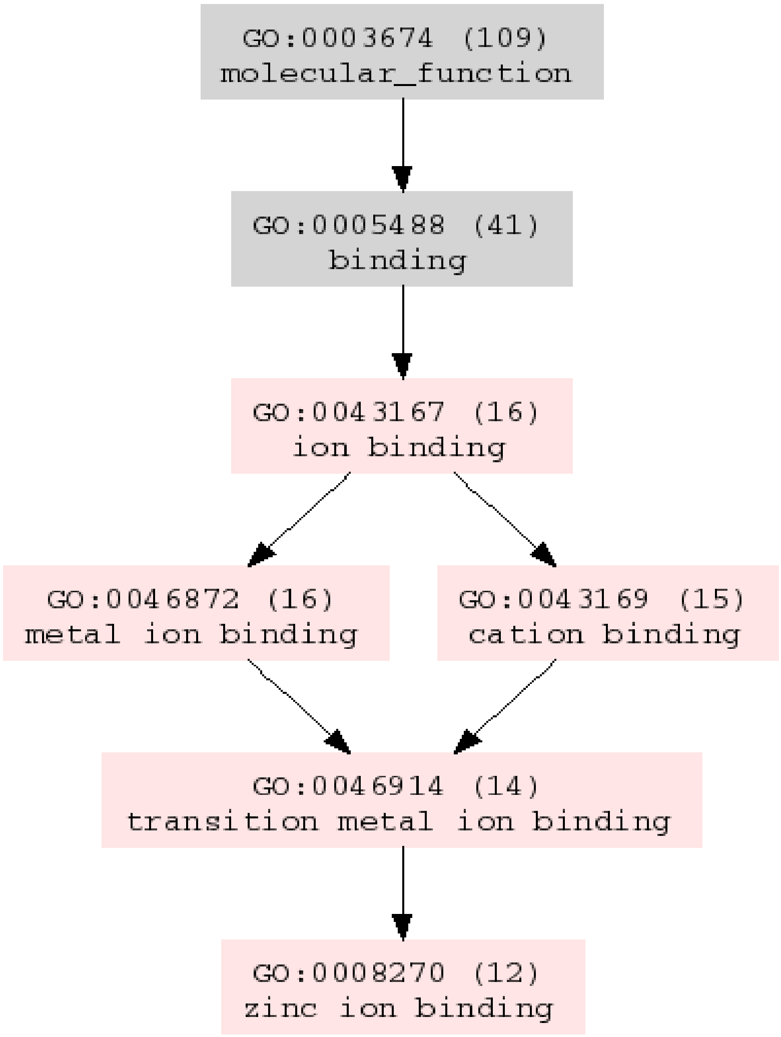

SUPPLEMENTARY DATA SET S3: Gene expression

Group C. upregulated in the root at early and late time treatment and at very early time treatment in the shoot.

fold change

		ROOTS							SHOOTS						
		EARLY			MIDDLE		Late		EARLY			MIDDLE		Late	
		10m	1h	2h	4h	24h	48	96h	10m	1h	2h	4h	24h	48	96h
Field1	PrimaryAccession	root1m	root2m	root3m	root4m	root5m	root6m	root7m	shoot1m	shoot2m	shoot3m	shoot4m	shoot5m	shoot6m	shoot7m
A_84_P576295	At1g03106.1	1.745	1.161	1.729	0.789	0.763	1.73	1.083	1.209	0.645	0.784	0.672	0.727	0.483	1.025
A_84_P23653	At1g03990.1	1.296	1.291	0.987	0.646	0.857	1.168	0.828	1.329	0.992	0.774	1.39	0.647	0.914	1.093
A_84_P22733	At1g04560.1	2.224	2.362	1.504	0.706	0.91	2.208	1.158	1.522	0.719	0.787	1.338	1.098	0.818	0.546
A_84_P11936	At1g29090.1	1.711	1.252	1.375	0.515	0.952	2.001	0.615	1.951	0.936	1.166	0.519	1.268	0.646	0.468
A_84_P14274	At1g52690.1	1.581	1.832	1.203	0.803	0.946	1.353	1.03	1.239	0.773	0.952	1.271	0.895	0.605	0.512
A_84_P10365	At1g54870.1	1.62	1.744	1.815	0.419	0.708	2.78	1.213	1.499	0.607	0.953	0.968	0.548	0.46	0.279
A_84_P549850	At1g60190.1	1.077	1.376	1.274	0.509	0.995	1.218	0.763	1.516	0.831	0.704	1.234	0.783	0.59	1.093
A_84_P20161	At2g15010.1	1.843	2.202	1.978	0.676	0.945	1.176	0.681	2.725	0.77	0.71	1.379	0.956	0.477	0.546
A_84_P522474	At2g19320.1	2.195	1.809	1.769	0.773	0.715	1.797	0.68	1.491	0.66	0.649	1.364	0.838	0.614	0.544
A_84_P17288	At2g28490.1	2.025	2.257	1.156	0.421	0.708	2.099	0.71	1.696	0.623	0.74	1.235	0.737	1.319	0.321
A_84_P18225	At2g29330.1	1.006	0.958	0.974	0.925	0.97	1.292	1.226	0.941	0.881	1.293	0.989	1.366	0.956	0.991
A_84_P22954	At2g29380.1	1.292	1.616	0.998	0.685	0.924	1.379	0.807	1.457	0.733	0.666	1.333	0.709	0.736	0.631
A_84_P17235	At2g34080.1	1.086	0.982	1.133	0.666	1.101	1.126	1.285	1.121	0.853	0.998	0.611	0.949	0.848	0.695
A_84_P10637	At2g40170.1	1.834	1.725	1.11	0.67	0.8	1.711	0.688	1.304	0.763	0.922	1.297	1.021	0.735	0.541
A_84_P22942	At2g41260.1	2.18	1.961	1.96	0.501	0.568	0.784	0.489	2.306	0.611	0.746	0.788	0.877	1.462	0.91
A_84_P22906	At2g42560.1	2.508	3.665	1.375	0.581	0.605	3.078	1.065	1.522	0.653	0.774	1.335	0.633	0.516	0.334
A_84_P551816	At3g03620.1	1.64	1.615	0.918	0.64	0.855	2.7	1.06	1.375	0.749	0.702	1.139	0.85	0.787	0.793
A_84_P19392	At3g53040.1	1.952	1.866	1.026	0.626	0.852	1.477	1.256	1.401	0.846	0.901	1.296	0.844	1.064	0.419
A_84_P11845	At3g56350.1	1.84	1.43	1.317	0.587	0.806	1.367	0.975	1.629	0.791	0.999	0.973	0.706	0.622	0.488
A_84_P12020	At4g16160.1	1.581	1.967	0.956	0.654	0.817	1.847	1.445	0.902	0.636	0.691	1.143	0.677	0.786	0.71
A_84_P11935	At4g21490.1	1.314	1.02	1.051	0.569	1.162	1.272	1.627	1.236	0.682	1.075	0.71	0.993	1.121	0.52
A_84_P12895	At4g25140.1	2.069	2.729	1.687	0.416	0.725	2.127	0.835	2.206	0.6	0.836	1.555	0.852	1.352	0.358
A_84_P14787	At4g25580.1	1.648	1.463	1.217	0.631	0.735	2.92	2.17	1.235	0.779	0.81	0.769	0.624	0.695	0.989
A_84_P11958	At4g26740.1	1.881	2.275	1.802	0.483	0.659	0.846	1.091	1.65	0.811	1.14	1.438	0.676	1.473	0.478
A_84_P11966	At4g28520.1	2.829	5.167	4.905	0.274	0.434	1.959	0.542	3.205	0.613	0.834	0.544	0.906	0.683	0.271
A_84_P14808	At4g30140.1	1.144	1.047	0.992	0.698	0.966	1.163	1.087	1.288	0.917	1.135	0.628	1.25	1.006	0.937
A_84_P23376	At4g36880.1	1.139	1.018	0.978	0.75	0.931	1.375	1.336	1.139	0.799	1.056	0.676	1.113	0.833	0.755
A_84_P20598	At5g22470.1	2.096	2.208	1.247	0.557	0.721	2.344	1.222	1.995	0.803	0.952	1.238	0.645	0.615	0.494
A_84_P15009	At5g54040.1	1.031	0.992	1.049	0.928	1.108	1.058	1.061	1.11	0.823	1.018	0.674	1.348	0.78	0.694
A_84_P20687	At5g54740.1	1.942	2.466	3.285	0.201	0.441	2.077	0.704	3.086	1.023	1.466	0.697	0.309	0.35	0.487
A_84_P18843	At5g66400.1	1.54	1.839	1.323	0.627	0.563	1.461	0.84	1.193	0.673	0.626	1.428	0.779	0.781	1.261

p value

		ROOTS							SHOOTS						
		EARLY			MIDDLE		Late		EARLY			MIDDLE		Late	
		10m	1h	2h	4h	24h	48	96h	10m	1h	2h	4h	24h	48	96h
Field1	PrimaryAccession	root1p	root2p	root3p	root4p	root5p	root6p	root7p	shoot1p	shoot2p	shoot3p	shoot4p	shoot5p	shoot6p	shoot7p
A_84_P576295	At1g03106.1	0.00789	0.592	0.259	0.343	0.0218	0.228	0.743	0.0462	0.144	0.351	0.0399	0.539	0.0954	0.886
A_84_P23653	At1g03990.1	0.0362	0.228	0.952	0.02	0.267	0.358	0.296	0.345	0.923	0.197	0.0144	0.366	0.775	0.83
A_84_P22733	At1g04560.1	0.000216	0.0347	0.343	0.208	0.463	0.0108	0.591	0.000457	0.158	0.129	0.116	0.906	0.758	0.0485
A_84_P11936	At1g29090.1	0.00531	0.29	0.0226	0.0887	0.888	0.282	0.401	0.0115	0.683	0.636	0.0573	0.586	0.228	0.0918
A_84_P14274	At1g52690.1	0.00481	0.0101	0.288	0.196	0.649	0.269	0.928	0.0217	0.184	0.661	0.118	0.909	0.311	0.24
A_84_P10365	At1g54870.1	0.00223	0.122	0.337	0.0996	0.0847	0.00706	0.2	0.0159	0.0424	0.812	0.801	0.458	0.202	0.0442
A_84_P549850	At1g60190.1	0.522	0.00787	0.0251	0.0249	0.961	0.487	0.476	0.0165	0.1	0.0181	0.275	0.746	0.184	0.791
A_84_P20161	At2g15010.1	0.0269	0.0469	0.197	0.391	0.871	0.793	0.47	0.00531	0.575	0.484	0.449	0.927	0.276	0.289
A_84_P522474	At2g19320.1	0.00361	0.0314	0.0848	0.548	0.46	0.289	0.504	0.0484	0.244	0.364	0.389	0.691	0.333	0.261
A_84_P17288	At2g28490.1	0.000426	0.0581	0.589	0.0513	0.319	0.0366	0.647	0.00243	0.162	0.27	0.463	0.699	0.654	0.0492
A_84_P18225	At2g29330.1	0.923	0.634	0.725	0.359	0.788	0.0238	0.0452	0.752	0.468	0.283	0.942	0.124	0.619	0.945
A_84_P22954	At2g29380.1	0.0116	0.0215	0.988	0.0565	0.457	0.017	0.612	0.00352	0.112	0.08	0.111	0.386	0.226	0.224
A_84_P17235	At2g34080.1	0.259	0.873	0.167	0.0334	0.325	0.169	0.0874	0.458	0.307	0.991	0.00172	0.758	0.394	0.0263
A_84_P10637	At2g40170.1	0.0059	0.0817	0.554	0.103	0.188	0.00537	0.221	0.0612	0.237	0.414	0.0638	0.975	0.512	0.0286
A_84_P22942	At2g41260.1	0.00182	0.0388	0.000772	0.0127	0.169	0.461	0.356	0.00531	0.0647	0.259	0.028	0.818	0.262	0.773
A_84_P22906	At2g42560.1	0.004	0.0011	0.237	0.198	0.235	0.0175	0.874	0.0107	0.0716	0.229	0.236	0.531	0.349	0.11
A_84_P551816	At3g03620.1	0.00338	0.086	0.613	0.0516	0.477	0.00245	0.838	0.0412	0.0895	0.0276	0.269	0.758	0.589	0.605
A_84_P19392	At3g53040.1	0.00239	0.0114	0.892	0.0724	0.186	0.026	0.0537	0.00526	0.218	0.487	0.179	0.798	0.902	0.0809
A_84_P11845	At3g56350.1	0.00032	0.181	0.0246	0.0273	0.164	0.17	0.892	0.00499	0.261	0.996	0.856	0.593	0.443	0.0852
A_84_P12020	At4g16160.1	0.0405	0.0173	0.888	0.0532	0.571	0.151	0.291	0.565	0.0433	0.101	0.351	0.529	0.614	0.255
A_84_P11935	At4g21490.1	0.00886	0.9	0.647	0.0503	0.31	0.117	0.0498	0.0548	0.123	0.65	0.145	0.981	0.695	0.0264
A_84_P12895	At4g25140.1	0.000179	0.0227	0.0501	0.0524	0.358	0.148	0.699	0.00886	0.277	0.512	0.288	0.811	0.542	0.082
A_84_P14787	At4g25580.1	0.00345	0.0138	0.376	0.0377	0.123	0.0175	0.0206	0.0468	0.141	0.0543	0.523	0.229	0.226	0.977
A_84_P11958	At4g26740.1	0.0043	0.0498	0.00693	0.0197	0.266	0.722	0.872	0.0773	0.545	0.377	0.113	0.106	0.207	0.162
A_84_P11966	At4g28520.1	0.00016	0.0168	0.115	0.074	0.149	0.204	0.409	0.00000664	0.446	0.8	0.36	0.904	0.536	0.161
A_84_P14808	At4g30140.1	0.223	0.696	0.922	0.0754	0.748	0.293	0.635	0.137	0.554	0.541	0.00115	0.0345	0.949	0.649
A_84_P23376	At4g36880.1	0.21	0.886	0.764	0.36	0.562	0.0363	0.0458	0.096	0.175	0.744	0.0418	0.624	0.382	0.21
A_84_P20598	At5g22470.1	0.00119	0.0394	0.249	0.0401	0.354	0.0221	0.735	0.0014	0.358	0.823	0.302	0.599	0.428	0.182
A_84_P15009	At5g54040.1	0.675	0.914	0.577	0.385	0.219	0.504	0.768	0.482	0.296	0.926	0.00418	0.0335	0.302	0.106
A_84_P20687	At5g54740.1	0.000012	0.0489	0.396	0.0648	0.112	0.165	0.574	0.00535	0.95	0.519	0.655	0.21	0.0805	0.134
A_84_P18843	At5g66400.1	0.000944	0.0403	0.206	0.18	0.0021	0.314	0.618	0.0594	0.0251	0.00747	0.0754	0.752	0.441	0.0287

SUPPLEMENTARY DATA SET S3: Functional characterization

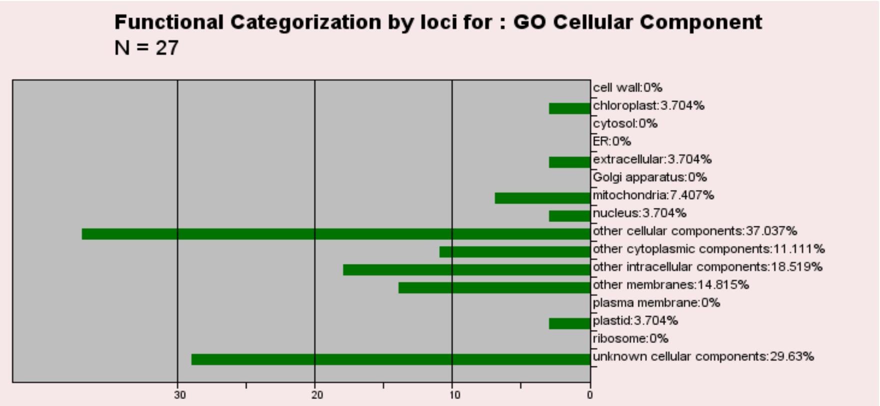

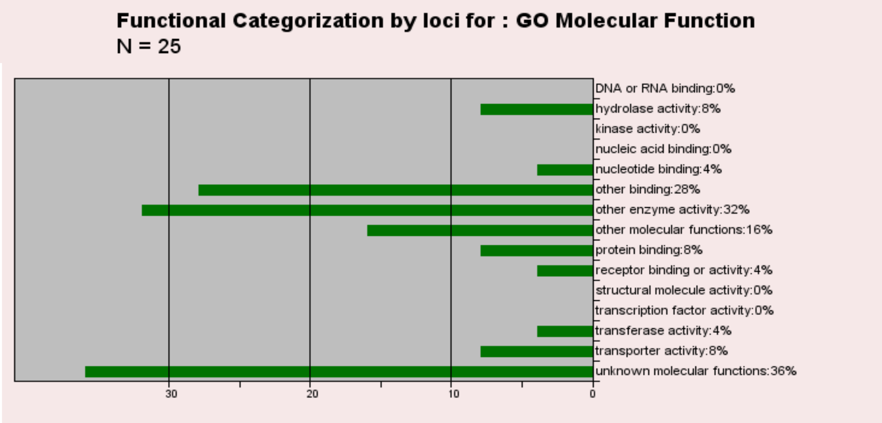

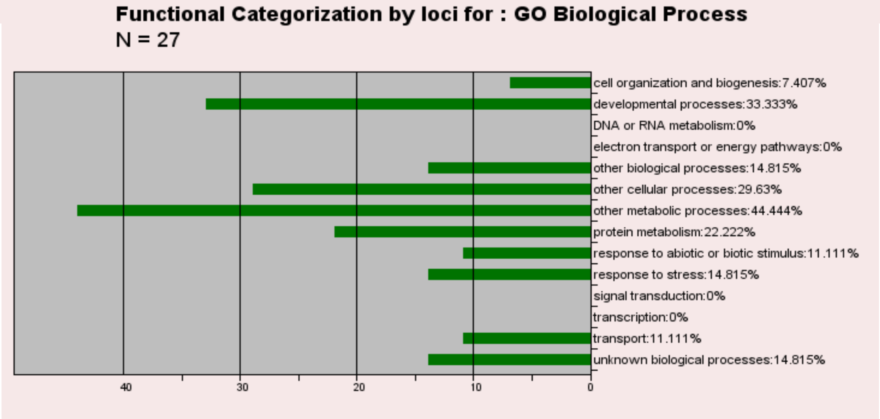

SUPPLEMENTARY DATA SET S3: GO Analysis

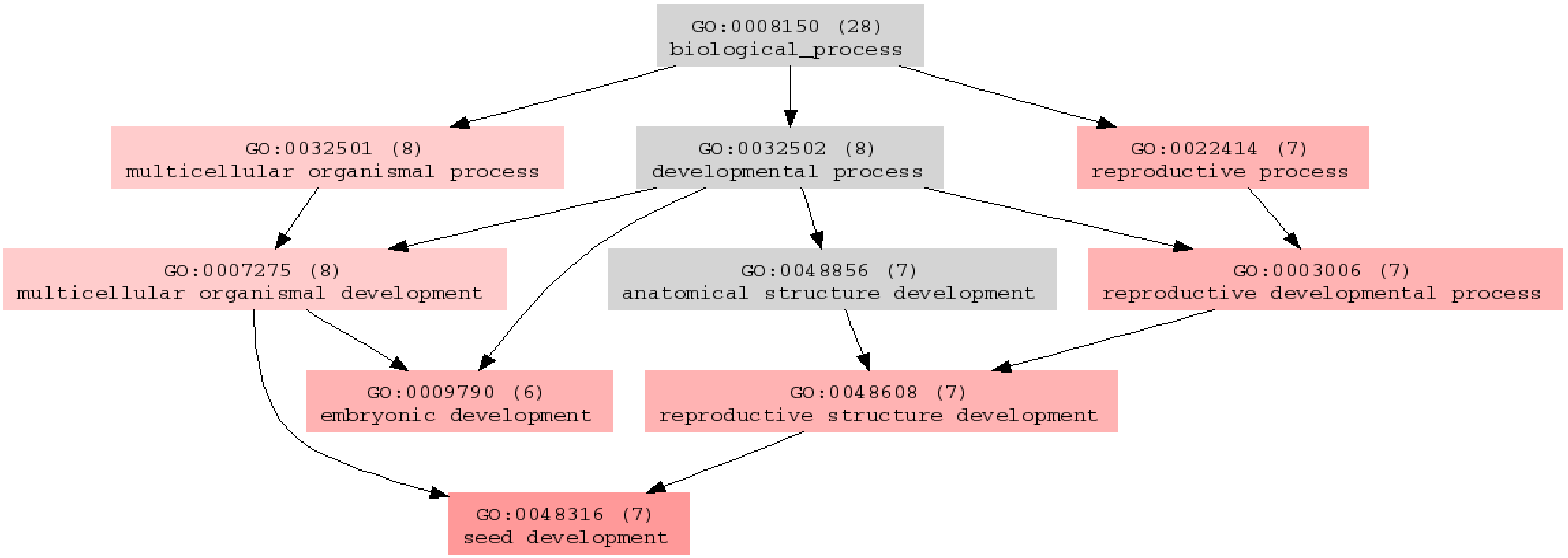

SUPPLEMENTARY DATA SET S4: Gene expression

Group D: genes strongly up-regulated at late time treatment

		fold change														p value													
		ROOTS							SHOOTS							ROOTS							SHOOTS						
		EARLY			MIDDLE		LATE		EARLY			MIDDLE		LATE		EARLY			MIDDLE		LATE		EARLY			MIDDLE		LATE	
		10m	1h	2h	4h	24h	48	96h	10m	1h	2h	4h	24h	48	96h	10m	1h	2h	4h	24h	48	96h	10m	1h	2h	4h	24h	48	96h
Field1	PrimaryAccession																												
A_84_P10559	At1g01560.1	1.202	0.992	0.888	1.025	1.241	0.836	1.791	0.912	1.02	1.413	0.94	0.821	0.768	1.717	0.27	0.941	0.329	0.831	0.0748	0.169	0.0312	0.69	0.92	0.117	0.841	0.218	0.248	0.0234
A_84_P21841	At1g02930.1	1.139	0.815	0.942	1.087	1.067	0.831	1.265	1.312	0.806	1.56	1.549	0.814	0.757	2.127	0.381	0.162	0.733	0.335	0.658	0.0911	0.061	0.221	0.0386	0.112	0.0548	0.757	0.0477	0.0456
A_84_P19923	At1g05680.1	0.93	0.987	0.955	1.362	0.87	0.93	1.278	0.885	0.848	1.389	1.386	0.889	0.689	1.259	0.37	0.88	0.572	0.104	0.165	0.375	0.0301	0.372	0.174	0.0216	0.436	0.618	0.264	0.0277
A_84_P598642	At1g07135.1	0.847	1.038	1.225	0.507	1.057	0.657	1.27	1.203	0.819	0.91	0.745	1.029	0.876	1.94	0.309	0.885	0.317	0.00847	0.845	0.132	0.0314	0.137	0.412	0.665	0.269	0.929	0.591	0.021
A_84_P522242	At1g07985.1	1.043	1.062	0.982	0.955	0.928	1.115	1.08	0.982	0.897	1.036	1.384	0.704	0.754	1.123	0.489	0.538	0.868	0.606	0.419	0.427	0.402	0.866	0.403	0.674	0.0245	0.0385	0.0291	0.287
A_84_P20470	At1g21910.1	0.993	1.036	1.071	0.656	1.146	0.883	1.423	0.903	1.149	1.053	0.78	1.044	1.006	1.313	0.948	0.664	0.399	0.043	0.151	0.195	0.00578	0.478	0.127	0.529	0.0378	0.623	0.944	0.12
A_84_P16097	At1g24140.1	0.946	0.949	1.133	0.648	1.018	0.813	1.255	1.565	0.971	1.26	1.306	0.853	0.853	1.967	0.607	0.72	0.341	0.0169	0.922	0.108	0.0358	0.00108	0.833	0.0769	0.213	0.316	0.131	0.00632
A_84_P19294	At1g26380.1	1.216	0.948	0.729	0.943	0.967	0.564	1.366	2.102	0.594	1.523	1.262	0.474	0.626	3.749	0.493	0.891	0.639	0.497	0.809	0.0655	0.0127	0.00808	0.075	0.276	0.436	0.233	0.0533	0.0207
A_84_P21931	At1g28370.1	0.878	0.967	1.054	0.531	1.182	0.863	1.229	1.251	1.009	1.026	0.919	1.109	0.744	2.065	0.364	0.822	0.77	0.0083	0.347	0.312	0.0437	0.237	0.966	0.831	0.711	0.759	0.109	0.0298
A_84_P16173	At1g28480.1	1.099	1.11	1.096	0.944	1.092	0.798	1.256	1.076	0.92	1.12	1.38	1.062	0.735	1.721	0.256	0.472	0.338	0.583	0.651	0.0381	0.097	0.629	0.749	0.545	0.105	0.868	0.0572	0.0408
A_84_P20998	At1g35140.1	1.139	1.08	1.367	0.635	1.068	0.673	2.182	1.077	0.938	1.021	0.718	0.899	1.109	1.341	0.329	0.598	0.00766	0.0188	0.657	0.0559	0.0608	0.677	0.534	0.873	0.0142	0.505	0.332	0.306
A_84_P12212	At1g56060.1	1.043	0.894	1.054	0.849	0.969	0.827	1.28	0.984	0.875	0.973	1.124	0.869	0.848	1.273	0.548	0.24	0.571	0.114	0.737	0.189	0.0271	0.827	0.239	0.808	0.298	0.322	0.188	0.0411
A_84_P242895	At1g66160.1	1.002	1	1.055	0.756	1.103	0.948	1.28	1.093	0.959	1.11	0.824	0.923	0.967	1.45	0.986	0.996	0.516	0.0408	0.443	0.697	0.0256	0.599	0.683	0.409	0.167	0.703	0.697	0.0366
A_84_P87559	At1g66500.1	0.929	1.113	1.028	0.583	1.419	0.721	1.004	1.218	0.865	0.999	1.038	1.244	0.913	1.371	0.501	0.319	0.804	0.0143	0.00916	0.143	0.977	0.304	0.381	0.995	0.885	0.61	0.593	0.0366
A_84_P210278	At1g66700.1	0.913	0.666	0.788	0.902	0.924	0.651	0.819	3.597	0.805	1.657	3.295	0.64	1.296	2.189	0.492	0.00426	0.0816	0.422	0.65	0.0311	0.0826	0.000724	0.46	0.287	0.013	0.408	0.343	0.0154
A_84_P15113	At1g71520.1	1.051	0.999	1.034	1.113	1.109	0.987	1.274	0.93	0.933	1.306	1.109	1.068	0.723	1.094	0.514	0.995	0.69	0.362	0.23	0.866	0.0243	0.558	0.559	0.0876	0.673	0.672	0.0405	0.416
A_84_P18998	At1g72900.1	0.994	0.977	0.972	0.822	1.087	0.981	1.433	1.128	0.925	1.187	0.926	0.897	0.879	1.703	0.934	0.826	0.745	0.058	0.366	0.879	0.00749	0.326	0.669	0.0771	0.663	0.298	0.278	0.0083
A_84_P19944	At1g72910.1	0.676	1.267	1.612	0.599	1.092	0.711	1.242	1.364	0.955	0.982	0.86	1.121	0.802	1.603	0.274	0.363	0.254	0.279	0.824	0.0339	0.513	0.0204	0.852	0.884	0.601	0.7	0.143	0.0303
A_84_P22782	At1g72950.1	0.981	1.009	0.965	0.747	1.131	0.976	0.993	1.082	0.928	1.03	0.779	1.078	0.972	1.218	0.768	0.916	0.695	0.0157	0.165	0.801	0.933	0.453	0.515	0.726	0.0481	0.716	0.725	0.06
A_84_P14331	At1g76650.1	0.989	1.111	1.095	0.678	1.221	0.779	1.323	1.218	0.786	1.108	0.863	1.021	0.951	2.201	0.958	0.595	0.51	0.0952	0.268	0.287	0.015	0.34	0.257	0.614	0.643	0.95	0.786	0.0223
A_84_P512655	At1g77640.1	0.969	1.021	0.974	0.717	1.089	0.816	1.515	1.078	0.873	0.915	0.742	1.135	1.15	1.305	0.76	0.857	0.823	0.044	0.537	0.173	0.00463	0.72	0.305	0.399	0.175	0.668	0.345	0.0363
A_84_P57250	At1g80160.1	0.947	1.006	1.143	0.951	1.013	0.717	0.919	1.068	0.822	1.182	1.049	0.716	1.267	1.811	0.482	0.965	0.246	0.741	0.898	0.0366	0.486	0.571	0.163	0.504	0.637	0.143	0.365	0.0318
A_84_P13271	At1g80840.1	0.839	0.957	1.043	0.506	1.084	0.671	1.302	1.365	1.059	1.188	0.769	0.941	0.965	2.363	0.309	0.864	0.847	0.0114	0.641	0.091	0.0476	0.174	0.885	0.119	0.474	0.884	0.91	0.0147
A_84_P108502	At2g02120.1	1.185	1.014	1.33	1.338	0.814	0.833	1.944	1.123	1.14	1.165	1.331	0.993	0.693	0.647	0.0415	0.929	0.0434	0.0133	0.101	0.08	0.247	0.193	0.155	0.317	0.0246	0.961	0.018	0.0223
A_84_P17941	At2g07718.1	0.996	0.948	0.999	0.69	1.082	0.695	1.56	1.071	0.867	0.803	1.015	1.195	1.219	1.123	0.978	0.653	0.99	0.0163	0.363	0.0885	0.0294	0.459	0.123	0.182	0.896	0.292	0.0496	0.304
A_84_P599750	At2g07779.1	0.981	0.938	0.89	0.846	1.055	0.722	1.23	0.907	0.954	1.11	1.139	1.102	1.064	1.119	0.906	0.467	0.306	0.173	0.573	0.0252	0.0466	0.449	0.66	0.381	0.264	0.435	0.547	0.325
A_84_P511815	At2g18193.1	0.871	0.943	1.022	1.314	0.943	1.044	1.377	0.882	0.945	1.129	1.111	0.824	0.657	1.145	0.131	0.524	0.836	0.146	0.493	0.574	0.0221	0.294	0.54	0.178	0.791	0.442	0.0266	0.297
A_84_P19231	At2g26150.1	0.804	1.025	1.215	0.949	0.885	0.675	1.507	0.905	1.407	1.19	0.896	0.728	0.77	1.069	0.0382	0.899	0.107	0.711	0.317	0.0465	0.0456	0.503	0.315	0.399	0.579	0.17	0.23	0.43
A_84_P12547	At2g30750.1	1.017	0.766	0.524	1.014	0.613	0.488	1.248	2.543	0.601	1.333	1.127	0.559	0.915	2.961	0.956	0.482	0.33	0.917	0.0359	0.0241	0.0781	0.00997	0.143	0.452	0.778	0.379	0.737	0.0235
A_84_P10657	At2g36800.1	1.048	0.971	0.946	1.274	1.087	1.164	2.791	0.987	0.995	0.92	0.992	0.798	0.786	1.555	0.884	0.898	0.815	0.573	0.599	0.629	0.0338	0.874	0.968	0.494	0.947	0.196	0.158	0.00673
A_84_P11659	At2g39400.1	1.022	1.065	1.218	0.776	1.062	1.005	1.343	1.019	0.901	1.166	0.893	1.018	1.254	1.487	0.834	0.687	0.0566	0.0788	0.716	0.982	0.0102	0.914	0.427	0.155	0.408	0.908	0.09	0.0332
A_84_P17343	At2g44840.1	0.763	0.894	1.09	0.427	1.073	0.635	1.709	1.296	1.38	1.223	1.005	1.097	0.733	2.374	0.171	0.636	0.777	0.0105	0.76	0.0428	0.0591	0.0393	0.521	0.294	0.987	0.735	0.148	0.0376
A_84_P19210	At2g46400.1	0.862	0.933	0.993	0.542	1.029	0.658	1.383	1.144	1.043	0.991	1.026	0.82	0.895	1.59	0.227	0.601	0.961	0.00882	0.88	0.0239	0.127	0.256	0.833	0.921	0.882	0.195	0.207	0.00208
A_84_P155845	At2g47950.1	0.917	0.97	0.949	0.93	0.897	0.689	1.354	0.875	1.338	1.019	1.236	1.018	1.096	1.699	0.276	0.756	0.608	0.602	0.643	0.00571	0.042	0.449	0.439	0.891	0.142	0.93	0.472	0.132
A_84_P66214	At3g02840.1	0.867	0.922	1.331	0.491	1.042	0.591	1.475	1.82	0.763	0.895	1.134	0.77	0.896	2.013	0.555	0.747	0.4	0.0537	0.861	0.095	0.00538	0.0335	0.374	0.493	0.749	0.534	0.584	0.00387
A_84_P15524	At3g23120.1	1.307	1.313	1.162	2.304	1.191	1.248	5.079	0.84	0.857	0.781	0.848	1.253	0.777	2.958	0.554	0.628	0.782	0.0891	0.66	0.689	0.00403	0.732	0.806	0.702	0.795	0.68	0.677	0.0472
A_84_P21194	At3g23240.1	0.858	1.025	1.039	0.734	1.061	0.911	1.405	1.206	0.954	1.356	1.665	0.94	0.79	2.209	0.159	0.785	0.673	0.0183	0.694	0.326	0.00905	0.512	0.834	0.295	0.05	0.718	0.316	0.0061
A_84_P22140	At3g23250.1	0.909	0.864	1.214	0.663	1.138	0.532	1.546	1.824	0.833	1.063	1.275	0.636	1.003	1.681	0.605	0.493	0.269	0.0274	0.629	0.0383	0.0382	0.0595	0.524	0.79	0.393	0.177	0.989	0.0358
A_84_P12725	At3g28510.1	1.28	1.135	1.057	0.975	1.016	0.576	1.969	1.037	1.371	1.057	1.228	1.424	0.396	1.298	0.132	0.577	0.711	0.926	0.842	0.0482	0.347	0.884	0.267	0.827	0.623	0.142	0.000747	0.677
A_84_P504929	At3g29000.1	0.86	0.997	1.066	0.614	1.237	0.858	1.229	1.904	0.956	1.093	0.775	1.412	0.959	2.839	0.239	0.99	0.718	0.0185	0.209	0.438	0.0435	0.0727	0.935	0.743	0.518	0.293	0.905	0.00186
A_84_P10846	At3g44350.1	0.525	0.823	0.853	0.934	0.927	0.773	3.6	0.783	1.338	1.268	1.415	1.103	0.998	3.116	0.241	0.742	0.794	0.904	0.897	0.66	0.0254	0.523	0.433	0.459	0.405	0.819	0.996	0.0253
A_84_P21275	At3g50930.1	0.867	0.952	1.031	0.752	1.14	0.761	1.56	1.265	0.842	1.05	1.01	0.893	0.804	1.719	0.101	0.678	0.737	0.132	0.301	0.108	0.0493	0.0647	0.294	0.549	0.94	0.586	0.102	0.00232
A_84_P14710	At3g56710.1	0.961	0.877	0.803	0.679	1.102	0.67	1.114	1.043	1.211	1.189	1.054	1.089	0.682	1.429	0.745	0.365	0.175	0.00716	0.555	0.0601	0.448	0.755	0.62	0.286	0.831	0.801	0.0153	0.00432
A_84_P156125	At3g61190.1	1.024	1.109	1.176	0.597	1.311	0.787	1.333	1.187	0.882	1.175	0.791	1.48	0.686	2.097	0.858	0.508	0.333	0.00667	0.0686	0.149	0.0153	0.506	0.638	0.482	0.497	0.392	0.163	0.0193
A_84_P17578	At4g10270.1	1.543	0.943	1.051	0.972	1.026	1.046	1.41	0.946	1.057	0.952	1.008	1.125	0.74	1.005	0.0273	0.583	0.664	0.911	0.834	0.784	0.0188	0.499	0.512	0.678	0.978	0.493	0.0432	0.956
A_84_P505907	At4g12735.1	0.904	0.947	1.186	1.572	1.411	0.802	1.788	1.012	0.919	0.85	0.96	1.038	0.472	0.998	0.263	0.611	0.122	0.0139	0.0411	0.388	0.00404	0.972	0.772	0.72	0.932	0.906	0.0776	0.996
A_84_P596516	At4g14450.1	0.977	0.97	1.267	0.702	1.123	0.713	1.293	1.598	0.766	1.398	0.901	1.125	0.929	1.782	0.865	0.881	0.187	0.0271	0.582	0.146	0.034	0.0491	0.128	0.26	0.558	0.641	0.749	0.00698
A_84_P211588	At4g24110.1	1.114	0.966	0.975	0.71	1.02	0.836	1.582	0.959	0.848	0.913	1	0.964	0.813	1.45	0.578	0.676	0.81	0.032	0.824	0.372	0.00864	0.755	0.218	0.289	0.999	0.697	0.179	0.0084
A_84_P15731	At4g25200.1	0.989	0.681	1.184	1.135	1.004	0.791	2.565	0.907	0.785	1.839	1.565	0.771	0.587	1.685	0.95	0.0148	0.316	0.781	0.973	0.214	0.0385	0.533	0.349	0.00831	0.486	0.684	0.238	0.0337
A_84_P15734	At4g25810.1	1.001	0.949	1.221	0.683	1.067	0.913	1.226	1.042	0.946	0.97	0.691	0.945	1.092	1.251	0.994	0.619	0.0495	0.0102	0.439	0.347	0.0439	0.798	0.532	0.729	0.00342	0.806	0.291	0.141
A_84_P11046	At4g34410.1	0.875	1.526	3.874	0.523	1.579	0.776	2.517	1.538	0.464	0.878	0.448	1.892	0.51	11.09	0.708	0.198	0.0344	0.142	0.278	0.557	0.00168	0.449	0.225	0.818	0.326	0.508	0.281	0.0204
A_84_P206058	At4g37290.1	0.837	0.707	0.813	0.948	1.051	0.884	1.531	2.058	1.086	1.112	1.317	0.617	0.693	3.292	0.282	0.0447	0.12	0.766	0.717	0.3	0.0311	0.0486	0.865	0.756	0.546	0.227	0.0856	0.00465
A_84_P15781	At4g37370.1	0.961	0.907	1.02	1.148	1.099	0.849	1.357	0.99	0.932	1.307	1.234	0.803	0.716	1.275	0.605	0.241	0.822	0.225	0.271	0.114	0.0133	0.938	0.432	0.0302	0.291	0.236	0.0895	0.0243
A_84_P554758	At4g37810.1	1.102	0.83	1.274	1.248	0.886	1.263	1.787	0.985	0.962	0.993	1.035	0.852	0.774	0.893	0.543	0.566	0.397	0.62	0.649	0.176	0.0152	0.881	0.629	0.94	0.734	0.355	0.0484	0.203
A_84_P12950	At4g38420.1	0.959	1.003	0.861	0.683	0.951	0.82	2.605	0.922	1.105	1.009	0.771	1.044	1.009	1.158	0.687	0.985	0.562	0.0299	0.728	0.0526	0.00723	0.261	0.24	0.901	0.0198	0.633	0.906	0.119
A_84_P91079	At5g22520.1	0.836	0.68	1.063	1.183	1.001	1.686	4.066	1.139	0.716	0.747	0.82	0.695	0.971	2.488	0.671	0.499	0.902	0.747	0.998	0.0603	0.00456	0.745	0.582	0.627	0.733	0.534	0.953	0.0383
A_84_P128501	At5g23480.1	1.276	1.24	0.903	1.348	1.041	0.882	1.768	0.873	1.23	0.825	0.854	0.773	0.763	0.73	0.327	0.569	0.787	0.403	0.89	0.476	0.0474	0.324	0.158	0.182	0.306	0.33	0.0431	0.0139
A_84_P515859	At5g25250.1	1.116	0.929	0.948	0.81	0.921	0.715	1.127	1.748	0.944	1.334	1.144	0.781	0.656	1.742	0.489	0.679	0.691	0.109	0.514	0.00688	0.386	0.00713	0.678	0.187	0.454	0.368	0.0348	0.123
A_84_P21578	At5g39670.1	0.877	0.797	0.839	0.583	0.965	0.726	1.507	1.574	1.103	1.427	1.151	1.321	0.683	2.312	0.402	0.244	0.423	0.00182	0.807	0.059	0.0781	0.0481	0.745	0.328	0.74	0.255	0.0282	0.0229
A_84_P13077	At5g42380.1	0.95	0.947	1.249	0.458	1.107	0.66	1.925	1.207	1.305	1.026	0.873	0.902	0.734	2.236	0.78	0.795	0.324	0.0223	0.688	0.139	0.00129	0.337	0.499	0.872	0.691	0.823	0.249	0.00272
A_84_P503480	At5g45630.1	1.035	1.082	1.11	1.266	1.152	0.814	1.833	1.163	0.942	0.808	0.815	0.918	0.545	2.601	0.85	0.586	0.486	0.21	0.523	0.333	0.0062	0.571	0.812	0.238	0.536	0.891	0.0823	0.00811
A_84_P14985	At5g47220.1	0.906	1.048	1.055	0.582	1.025	0.698	1.805	0.955	1.043	1.038	1.081	0.881	1.008	1.847	0.498	0.648	0.565	0.0263	0.903	0.00499	0.0204	0.665	0.726	0.748	0.337	0.48	0.959	0.102
A_84_P13104	At5g49520.1	1.079	0.981	1.16	0.71	1.139	0.761	1.624	1.193	0.867	1.103	0.987	0.863	1.109	1.4	0.51	0.812	0.123	0.0152	0.288	0.0218	0.106	0.231	0.259	0.401	0.885	0.31	0.436	0.0186
A_84_P249495	At5g52760.1	0.773	0.899	1.042	1.035	1.458	0.952	6.372	1.213	1.205	0.928	0.934	0.946	1.833	5.993	0.615	0.861	0.941	0.952	0.477	0.894	0.00881	0.667	0.733	0.9	0.905	0.931	0.124	0.00383
A_84_P13130	At5g57220.1	1.276	0.841	0.878	1.098	1.082	0.906	1.216	1.331	0.946	1.497	1.248	1.574	0.834	1.691	0.176	0.375	0.582	0.331	0.579	0.267	0.0487	0.0637	0.562	0.0829	0.0951	0.462	0.074	0.00462
A_84_P537220	At5g57510.1	1.014	1.192	1.166	0.617	1.5	1.025	1.246	1.572	0.669	1.005	1.273	1.227	0.589	1.111	0.921	0.392	0.376	0.0447	0.0371	0.86	0.325	0.224	0.262	0.989	0.594	0.703	0.267	0.831
A_84_P16923	At5g59820.1	0.894	1.058	1.009	0.743	1.102	0.753	1.138	1.201	1.266	1.046	0.983	1.033	0.694	1.773	0.332	0.704	0.945	0.0463	0.564	0.0202	0.142	0.408	0.43	0.786	0.958	0.933	0.139	0.0233

SUPPLEMENTARY DATA SET S4: Functional characterization

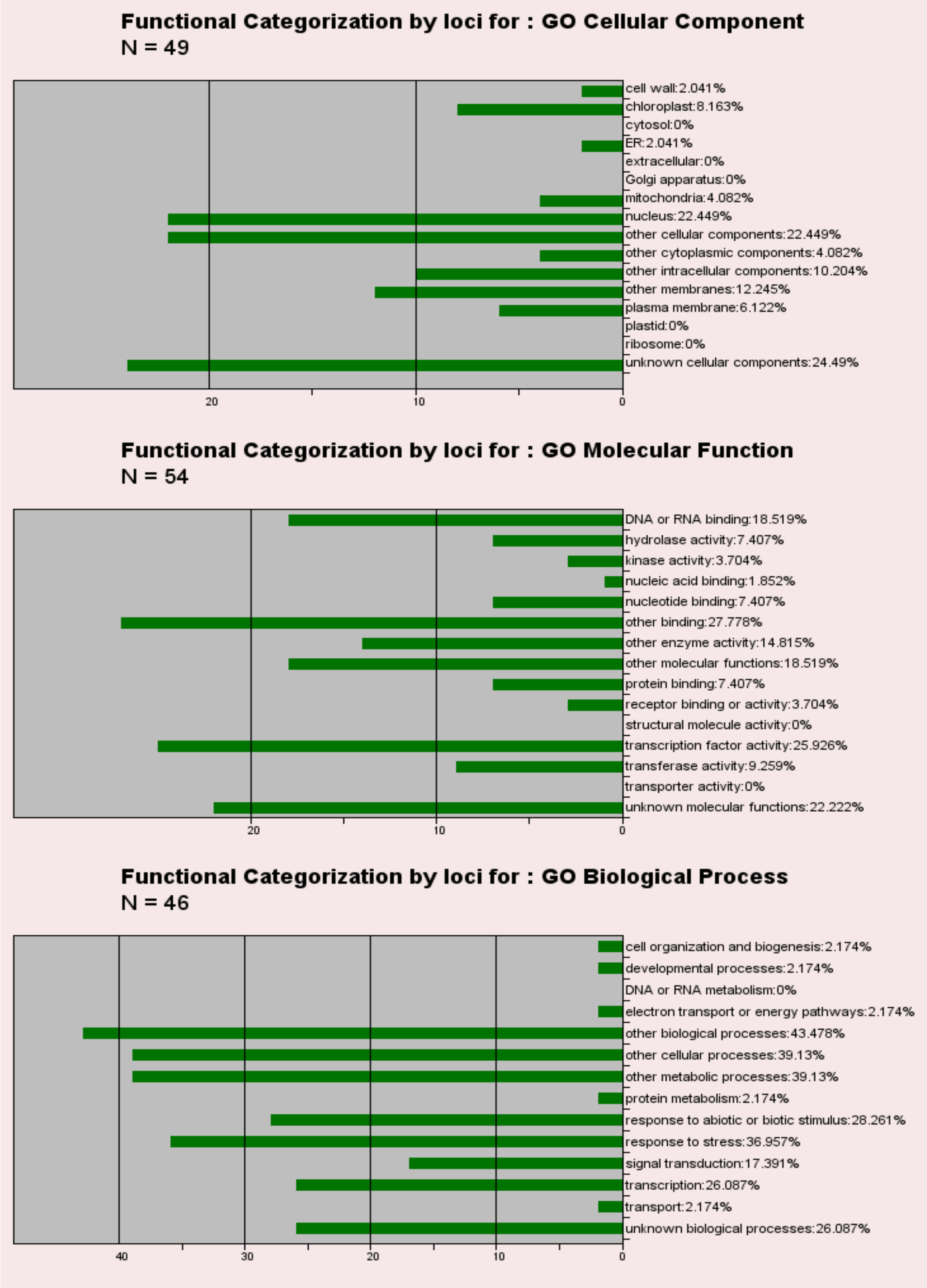

SUPPLEMENTARY DATA SET S4: GO Analysis

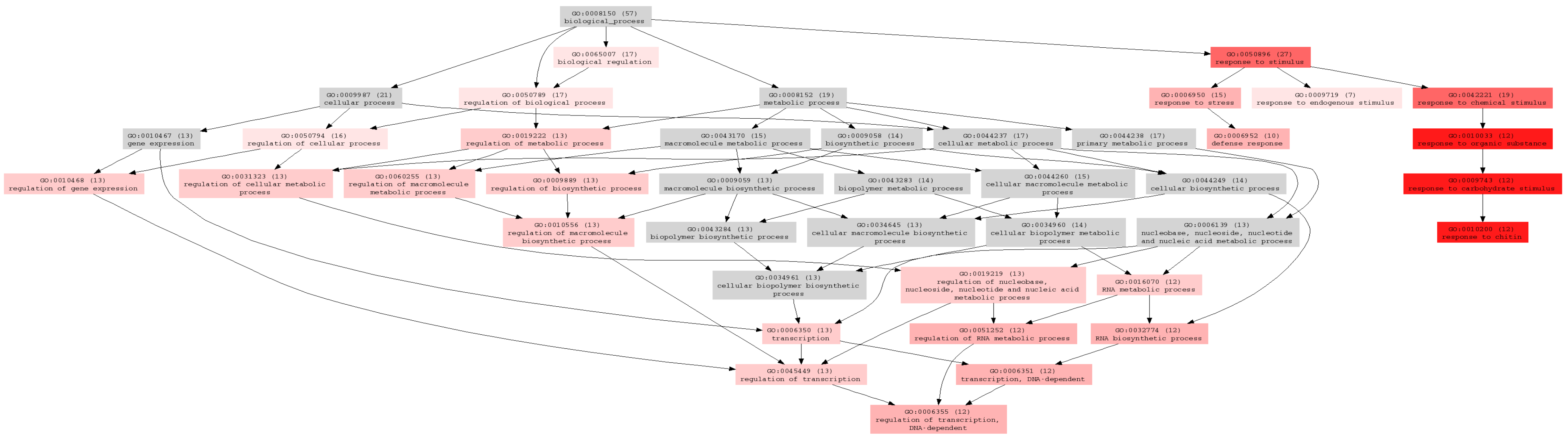

SUPPLEMENTARY DATA SET S5: Gene expression

Group E: genes down-regulated in the root and up-regulated in the shoot at all times

		fold change														p value													
		ROOTS							SHOOTS							ROOTS							SHOOTS						
		EARLY			MIDDLE		LATE		EARLY			MIDDLE		LATE		EARLY			MIDDLE		LATE		EARLY			MIDDLE		LATE	
		10m	1h	2h	4h	24h	48h	96h	10m	1h	2h	4h	24h	48h	96h	10m	1h	2h	4h	24h	48h	96h	10m	1h	2h	4h	24h	48h	96h
Field1	PrimaryAccession																												
A_84_P556826	At1g16410.2	0.923	0.854	0.772	0.792	1.136	0.925	0.870	1.354	2.253	1.884	1.857	2.733	2.387	2.177	0.868	0.800	0.686	0.716	0.826	0.900	0.824	0.525	0.032	0.197	0.244	0.019	0.015	0.014
A_84_P134545	At1g17610.1	0.839	0.798	0.818	0.678	0.918	0.882	1.157	1.644	1.277	1.108	1.192	1.420	2.017	2.112	0.707	0.719	0.754	0.542	0.891	0.842	0.804	0.158	0.555	0.789	0.642	0.183	0.034	0.005
A_84_P17171	At1g19510.1	0.852	0.457	0.919	0.996	0.821	0.726	0.754	1.651	1.582	1.120	1.269	1.683	1.567	1.280	0.729	0.203	0.889	0.995	0.747	0.616	0.654	0.001	0.004	0.253	0.318	0.136	0.006	0.040
A_84_P15258	At1g20120.1	0.686	0.763	0.746	0.991	1.177	0.678	1.081	1.477	1.038	1.542	1.595	2.030	1.852	1.516	0.465	0.660	0.635	0.987	0.763	0.525	0.896	0.339	0.945	0.393	0.328	0.047	0.041	0.010
A_84_P63350	At1g29720.1	0.608	0.675	1.158	0.703	0.954	0.733	0.881	1.955	2.726	2.159	1.259	1.081	1.134	1.209	0.344	0.531	0.825	0.567	0.933	0.611	0.824	0.025	0.021	0.034	0.538	0.457	0.306	0.096
A_84_P547148	At1g41650.1	0.551	0.638	0.601	1.738	1.064	0.476	1.123	0.844	2.852	2.388	2.082	0.964	1.899	2.353	0.265	0.475	0.414	0.264	0.906	0.239	0.816	0.669	0.004	0.074	0.290	0.927	0.041	0.003
A_84_P61530	At1g51840.1	0.808	0.810	0.931	0.732	0.809	0.995	0.883	2.101	1.242	1.088	1.127	1.521	1.142	1.175	0.027	0.153	0.590	0.010	0.255	0.975	0.161	0.002	0.200	0.581	0.471	0.045	0.366	0.403
A_84_P10415	At1g52030.1	0.847	1.053	0.765	0.671	0.812	0.530	0.834	1.705	1.052	1.004	3.080	1.719	1.164	0.944	0.706	0.918	0.637	0.510	0.685	0.343	0.763	0.028	0.811	0.990	0.006	0.035	0.149	0.766
A_84_P19999	At1g52770.1	0.839	0.823	0.800	0.934	0.902	0.861	1.489	2.507	1.359	1.166	2.218	2.038	1.649	1.932	0.730	0.759	0.727	0.910	0.869	0.812	0.497	0.026	0.529	0.787	0.099	0.195	0.025	0.039
A_84_P12256	At1g56710.1	0.777	0.687	0.666	0.584	0.777	0.691	0.663	2.132	2.188	1.646	1.645	2.022	1.804	1.700	0.615	0.566	0.511	0.389	0.688	0.566	0.513	0.025	0.018	0.082	0.030	0.010	0.001	0.008
A_84_P66984	At1g64360.1	0.936	0.834	0.823	0.780	1.065	0.875	0.754	1.406	1.893	1.438	1.528	1.562	2.217	2.064	0.886	0.774	0.763	0.670	0.910	0.834	0.640	0.276	0.114	0.294	0.269	0.085	0.001	0.000
A_84_P19861	At1g64480.1	0.799	0.884	0.827	0.791	0.916	1.006	0.837	1.409	1.689	1.071	1.248	1.774	0.990	1.360	0.028	0.240	0.054	0.043	0.387	0.964	0.071	0.081	0.081	0.762	0.433	0.039	0.961	0.164
A_84_P581848	At1g68480.1	0.824	0.745	0.725	0.702	0.981	1.058	0.875	1.939	1.117	1.671	2.185	1.537	1.532	1.314	0.688	0.599	0.597	0.578	0.974	0.922	0.826	0.016	0.718	0.053	0.005	0.043	0.009	0.116
A_84_P12426	At1g68890.1	0.731	0.600	0.815	0.844	0.636	0.626	0.822	1.782	1.369	1.481	1.863	1.421	1.867	1.957	0.525	0.441	0.726	0.768	0.478	0.473	0.738	0.089	0.364	0.253	0.104	0.341	0.046	0.049
A_84_P580100	At1g77960.1	0.745	0.671	0.853	0.633	0.882	0.912	0.805	1.444	2.691	1.008	1.905	1.538	1.599	1.432	0.557	0.540	0.788	0.472	0.824	0.874	0.703	0.178	0.014	0.970	0.076	0.177	0.009	0.031
A_84_P216568	At1g78030.1	0.855	0.827	0.790	0.813	0.890	0.898	1.005	1.487	2.101	2.228	1.325	1.369	1.843	2.048	0.741	0.761	0.715	0.746	0.847	0.852	0.994	0.159	0.043	0.055	0.503	0.496	0.003	0.002
A_84_P53450	At1g79770.1	0.627	0.555	0.347	0.590	0.575	0.585	0.499	1.378	1.756	1.380	1.554	1.594	1.414	1.704	0.344	0.378	0.119	0.419	0.389	0.416	0.279	0.009	0.004	0.034	0.019	0.006	0.017	0.003
A_84_P17311	At2g04420.1	0.725	0.620	0.845	0.664	0.784	0.820	0.663	1.517	1.634	1.893	1.237	1.607	3.095	1.937	0.529	0.433	0.771	0.512	0.697	0.740	0.505	0.265	0.259	0.166	0.672	0.172	0.019	0.039
A_84_P18175	At2g21610.1	0.751	0.789	0.642	0.848	0.699	1.094	0.608	1.244	1.876	1.605	1.198	1.833	1.231	1.433	0.009	0.034	0.003	0.271	0.054	0.687	0.239	0.352	0.107	0.162	0.568	0.076	0.426	0.285
A_84_P147178	At2g21890.1	0.699	0.665	0.626	0.664	0.566	0.729	0.771	1.530	1.364	1.008	1.051	1.281	1.739	1.418	0.417	0.509	0.452	0.499	0.370	0.599	0.638	0.040	0.149	0.961	0.866	0.138	0.028	0.019
A_84_P558814	At2g25780.1	0.700	0.906	0.786	0.968	0.881	0.842	0.881	1.514	1.851	1.466	1.278	1.589	1.882	2.287	0.465	0.872	0.706	0.956	0.839	0.785	0.839	0.003	0.002	0.012	0.246	0.004	0.033	0.000
A_84_P15388	At2g29300.1	0.786	0.702	0.820	1.330	0.950	0.583	0.605	1.213	1.578	1.234	0.806	0.994	1.537	1.468	0.565	0.567	0.722	0.608	0.932	0.417	0.443	0.081	0.002	0.044	0.325	0.948	0.033	0.004
A_84_P11549	At2g30560.1	0.598	0.589	0.573	0.762	0.645	0.614	0.631	1.797	1.873	1.502	2.087	1.595	2.246	1.825	0.332	0.427	0.406	0.651	0.497	0.457	0.478	0.022	0.015	0.060	0.011	0.029	0.004	0.018
A_84_P600694	At2g30900.1	0.685	0.740	0.880	0.876	0.911	0.996	0.749	1.454	1.223	1.403	0.912	1.343	1.921	1.820	0.430	0.628	0.837	0.831	0.880	0.994	0.606	0.198	0.579	0.223	0.834	0.229	0.010	0.031
A_84_P17327	At2g34210.1	0.690	0.728	0.550	0.886	0.827	1.058	0.760	1.218	1.332	1.223	1.323	1.267	2.237	1.391	0.422	0.584	0.321	0.814	0.713	0.903	0.629	0.503	0.379	0.618	0.436	0.500	0.001	0.045
A_84_P240665	At2g36610.1	0.838	0.825	0.750	0.840	0.900	0.853	0.883	1.692	1.809	1.486	1.286	2.866	1.902	1.905	0.727	0.763	0.651	0.782	0.866	0.801	0.843	0.145	0.068	0.173	0.418	0.007	0.034	0.027
A_84_P556217	At2g38995.1	0.822	0.715	0.685	0.773	0.782	0.952	0.871	1.356	1.958	1.235	2.067	2.268	1.779	1.385	0.674	0.602	0.560	0.680	0.696	0.934	0.857	0.424	0.203	0.682	0.156	0.038	0.001	0.007
A_84_P111562	At2g42830.1	0.546	0.572	0.484	0.798	0.812	0.601	0.749	1.540	1.174	1.258	1.115	1.525	2.058	1.884	0.250	0.388	0.290	0.689	0.713	0.421	0.574	0.236	0.660	0.468	0.814	0.176	0.014	0.007
A_84_P175924	At2g44745.1	0.521	0.556	0.648	0.593	0.699	0.690	0.707	1.280	1.341	1.084	1.476	1.426	1.789	1.634	0.195	0.282	0.506	0.413	0.536	0.510	0.583	0.239	0.279	0.713	0.195	0.061	0.004	0.003
A_84_P602531	At2g46880.1	1.203	1.453	0.812	1.226	0.921	0.875	0.918	1.291	1.894	1.604	5.098	1.691	3.309	2.978	0.664	0.558	0.744	0.747	0.895	0.831	0.891	0.530	0.223	0.442	0.034	0.411	0.000	0.029
A_84_P603414	At2g46940.1	0.849	0.842	0.858	0.614	0.928	0.880	0.889	1.613	1.179	1.271	1.517	1.712	1.709	1.197	0.043	0.078	0.182	0.002	0.396	0.199	0.227	0.016	0.372	0.204	0.042	0.083	0.051	0.147
A_84_P301610	At3g12470.1	0.761	0.817	0.658	0.830	0.723	0.700	0.734	1.926	1.788	1.419	1.891	2.637	1.922	2.401	0.572	0.737	0.462	0.754	0.609	0.577	0.622	0.014	0.034	0.096	0.031	0.009	0.022	0.090
A_84_P23096	At3g26200.1	1.053	0.729	0.650	0.777	0.540	0.662	1.416	1.458	1.334	1.141	1.650	1.775	2.445	1.724	0.907	0.612	0.489	0.655	0.289	0.511	0.557	0.350	0.596	0.796	0.305	0.123	0.000	0.001
A_84_P169223	At3g48310.1	0.900	0.813	0.975	1.144	0.803	0.758	0.642	1.221	1.689	1.118	1.139	1.154	1.247	1.509	0.781	0.733	0.963	0.796	0.723	0.662	0.437	0.158	0.002	0.245	0.376	0.190	0.037	0.002
A_84_P519134	At3g50450.1	0.852	0.665	0.873	0.822	0.889	0.901	0.872	1.712	2.594	1.843	1.529	1.270	2.636	4.111	0.750	0.521	0.826	0.757	0.850	0.866	0.827	0.212	0.073	0.232	0.411	0.610	0.047	0.003
A_84_P15628	At3g57240.1	0.713	0.677	0.689	0.773	0.736	0.736	1.124	1.444	1.639	2.130	1.456	1.954	1.378	2.104	0.496	0.551	0.564	0.679	0.627	0.630	0.846	0.340	0.294	0.068	0.266	0.041	0.027	0.015
A_84_P550572	At3g59230.1	0.821	0.810	0.782	0.805	0.883	0.841	0.886	1.886	1.540	1.868	1.325	2.923	2.457	3.004	0.698	0.740	0.700	0.733	0.841	0.783	0.845	0.039	0.373	0.193	0.519	0.010	0.026	0.013
A_84_P10911	At3g59510.1	0.805	0.807	0.773	0.859	0.869	0.821	0.883	1.816	2.218	1.478	2.408	1.967	1.613	1.721	0.673	0.738	0.690	0.808	0.823	0.758	0.842	0.008	0.007	0.049	0.006	0.022	0.090	0.014
A_84_P591116	At3g60890.1	0.808	0.901	0.779	0.816	0.919	0.834	0.860	1.339	1.591	1.938	1.481	2.616	2.691	2.369	0.678	0.865	0.698	0.749	0.891	0.775	0.811	0.513	0.393	0.213	0.472	0.040	0.008	0.001
A_84_P22322	At4g13810.1	0.911	0.852	0.710	0.865	0.831	0.720	0.879	1.880	1.546	1.228	1.525	1.422	1.627	1.692	0.848	0.798	0.583	0.816	0.762	0.588	0.837	0.012	0.008	0.095	0.020	0.029	0.001	0.002
A_84_P22327	At4g18960.1	0.787	0.781	0.848	1.139	0.798	0.665	0.936	1.209	2.181	1.103	1.234	1.165	1.979	1.823	0.620	0.675	0.791	0.820	0.704	0.510	0.907	0.613	0.140	0.798	0.673	0.673	0.043	0.014
A_84_P312233	At4g23230.1	0.777	0.797	0.764	0.782	0.770	0.981	0.859	1.947	1.376	2.617	1.949	2.073	2.160	2.544	0.607	0.705	0.674	0.691	0.666	0.974	0.808	0.041	0.517	0.018	0.154	0.046	0.014	0.002
A_84_P19506	At4g23250.1	0.492	0.740	0.764	0.632	0.618	0.776	0.995	1.177	1.131	1.368	1.024	1.024	1.358	1.424	0.140	0.590	0.658	0.464	0.413	0.658	0.993	0.092	0.277	0.018	0.848	0.823	0.021	0.011
A_84_P16712	At4g32990.1	0.804	0.628	0.753	0.624	0.690	0.780	0.715	2.117	1.415	1.920	1.061	1.469	2.453	1.714	0.646	0.478	0.628	0.472	0.560	0.655	0.592	0.010	0.154	0.035	0.870	0.071	0.011	0.012
A_84_P19619	At5g07700.1	0.783	0.747	0.735	0.928	0.594	1.393	1.008	1.314	1.641	1.514	3.127	1.667	1.684	1.312	0.625	0.649	0.632	0.900	0.409	0.500	0.990	0.407	0.037	0.060	0.017	0.022	0.002	0.174
A_84_P13010	At5g11190.1	0.546	0.578	0.703	0.655	0.661	0.549	0.876	1.354	1.347	1.579	1.434	1.467	1.244	1.267	0.243	0.406	0.571	0.509	0.516	0.351	0.826	0.299	0.107	0.044	0.101	0.030	0.043	0.026
A_84_P511391	At5g17830.1	0.826	0.859	0.702	0.716	0.758	0.702	0.705	1.804	1.489	1.506	1.327	2.102	2.011	1.409	0.682	0.795	0.578	0.561	0.657	0.581	0.571	0.024	0.080	0.152	0.261	0.024	0.016	0.046
A_84_P66174	At5g18000.1	0.919	0.684	0.472	0.928	0.702	0.545	0.743	1.724	1.238	1.089	1.185	0.824	2.403	1.727	0.830	0.533	0.222	0.876	0.545	0.321	0.568	0.011	0.300	0.713	0.378	0.253	0.002	0.013
A_84_P586001	At5g25970.1	0.821	0.857	0.800	0.759	0.938	0.895	0.857	1.651	2.415	1.658	1.476	1.867	1.401	1.604	0.686	0.808	0.704	0.652	0.918	0.860	0.802	0.275	0.085	0.194	0.198	0.026	0.024	0.014
A_84_P553076	At5g34830.1	1.111	0.664	0.956	1.089	0.834	0.546	0.795	1.453	1.390	1.287	1.450	0.857	1.471	1.375	0.817	0.470	0.925	0.869	0.723	0.335	0.677	0.152	0.245	0.238	0.338	0.410	0.048	0.024
A_84_P606500	At5g40155.1	0.708	0.629	0.621	0.519	0.735	0.614	0.759	1.866	2.170	1.929	1.879	1.722	2.485	2.721	0.469	0.467	0.434	0.316	0.610	0.374	0.634	0.047	0.083	0.165	0.134	0.149	0.025	0.032
A_84_P137009	At5g44420.1	0.839	0.675	0.594	0.783	0.735	0.779	1.197	1.452	1.069	1.145	1.208	2.353	2.531	2.232	0.722	0.543	0.424	0.665	0.625	0.689	0.744	0.335	0.903	0.791	0.707	0.016	0.000	0.020
A_84_P594938	At5g46490.2	0.776	0.584	0.840	0.499	1.510	1.131	0.644	1.298	1.570	1.753	1.628	1.022	1.344	1.379	0.530	0.356	0.740	0.271	0.352	0.802	0.457	0.365	0.049	0.092	0.121	0.887	0.043	0.029
A_84_P21605	At5g46830.1	0.720	1.091	0.902	0.600	0.636	1.012	0.938	1.392	1.271	1.217	2.779	2.054	0.787	0.872	0.470	0.865	0.854	0.389	0.442	0.983	0.904	0.394	0.317	0.424	0.006	0.039	0.221	0.589
A_84_P22552	At5g47130.1	0.785	0.649	0.699	0.646	0.712	0.828	0.704	1.448	1.631	1.697	2.038	1.370	1.628	1.563	0.619	0.508	0.569	0.502	0.592	0.752	0.581	0.123	0.095	0.058	0.030	0.326	0.018	0.031
A_84_P15944	At5g50790.1	0.747	0.773	0.817	0.904	0.915	0.877	0.454	1.651	2.585	2.245	1.382	3.324	3.641	2.454	0.561	0.684	0.754	0.871	0.887	0.836	0.229	0.125	0.046	0.016	0.259	0.070	0.000	0.000
A_84_P582570	At5g51530.1	0.756	0.661	0.771	0.654	0.811	1.001	0.766	2.000	1.225	1.319	0.903	1.164	2.571	2.011	0.545	0.524	0.669	0.515	0.729	0.998	0.658	0.052	0.641	0.554	0.818	0.701	0.014	0.007
A_84_P110392	At5g55450.1	0.843	0.777	0.774	0.903	0.859	0.813	0.867	1.652	1.839	1.498	1.876	1.795	2.306	2.022	0.735	0.689	0.692	0.868	0.809	0.745	0.807	0.231	0.247	0.424	0.245	0.235	0.029	0.008
A_84_P567338	At5g55570.1	0.717	0.680	0.631	1.058	1.018	0.727	1.380	1.236	1.761	0.934	1.883	1.229	1.580	1.418	0.491	0.531	0.423	0.914	0.968	0.526	0.492	0.311	0.025	0.610	0.046	0.069	0.004	0.012
A_84_P10289	At5g55720.1	0.839	0.772	0.694	0.858	0.659	1.001	1.357	1.181	1.430	1.335	1.863	1.673	1.174	1.114	0.705	0.669	0.554	0.793	0.513	0.998	0.658	0.695	0.524	0.590	0.042	0.018	0.087	0.206
A_84_P230519	At5g56490.1	0.691	0.638	0.722	0.594	0.819	0.832	0.769	1.691	1.512	1.252	1.322	1.361	1.981	1.542	0.475	0.485	0.605	0.414	0.743	0.745	0.672	0.017	0.097	0.304	0.247	0.163	0.001	0.004
A_84_P21650	At5g59330.1	0.903	0.843	0.763	0.637	0.772	0.842	0.921	1.037	1.135	1.187	1.153	3.562	1.590	1.983	0.838	0.782	0.667	0.496	0.680	0.785	0.891	0.939	0.813	0.750	0.793	0.001	0.004	0.002
A_84_P530196	At5g60130.1	0.698	0.647	0.719	0.653	0.716	0.773	0.670	1.447	1.404	1.390	1.881	1.526	2.033	2.223	0.446	0.502	0.594	0.508	0.594	0.672	0.534	0.187	0.099	0.111	0.097	0.099	0.007	0.009
A_84_P233609	At5g62080.1	0.626	0.621	0.603	0.611	0.679	0.647	0.667	2.559	2.169	1.757	2.310	2.491	1.885	3.024	0.374	0.471	0.446	0.457	0.546	0.504	0.530	0.012	0.032	0.076	0.048	0.036	0.183	0.027

SUPPLEMENTARY DATA SET S5: Functional characterization

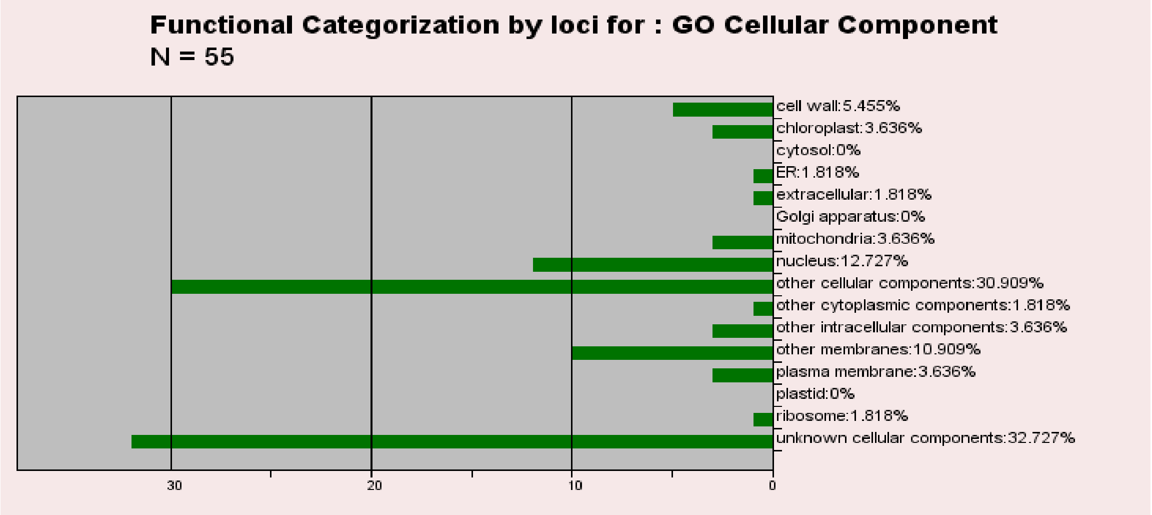

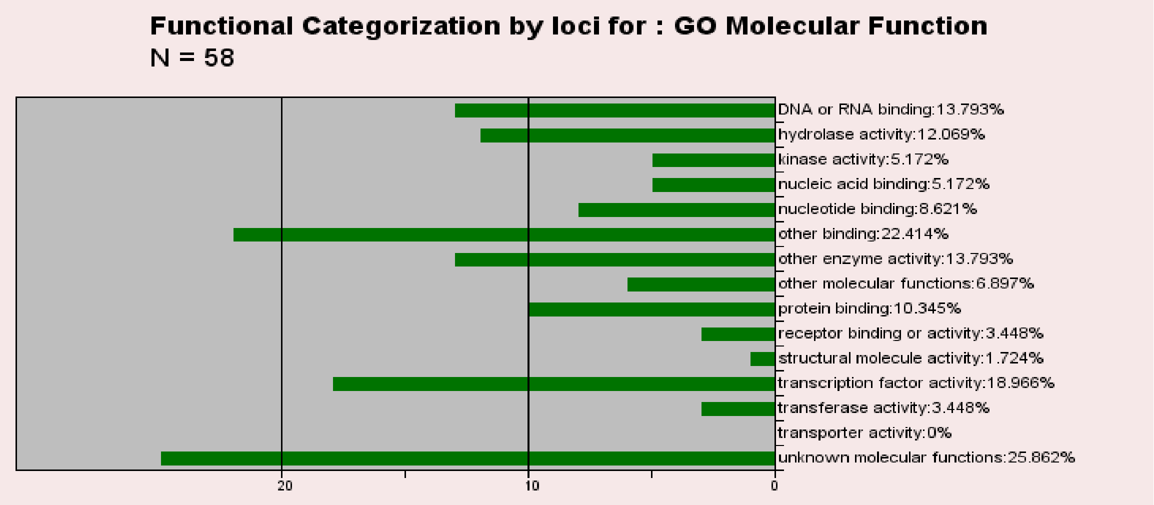

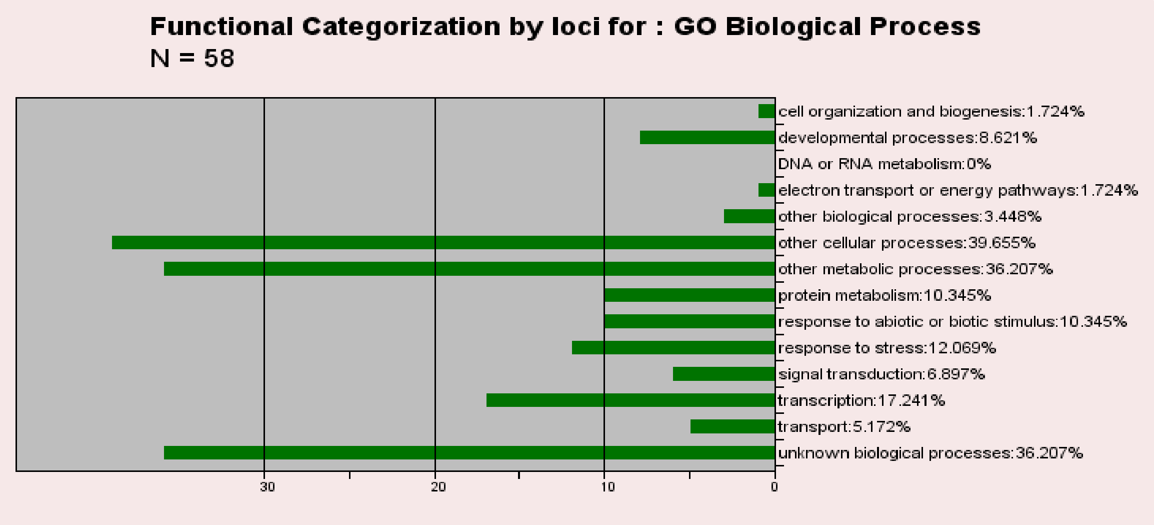

SUPPLEMENTARY DATA SET S5: GO Analysis

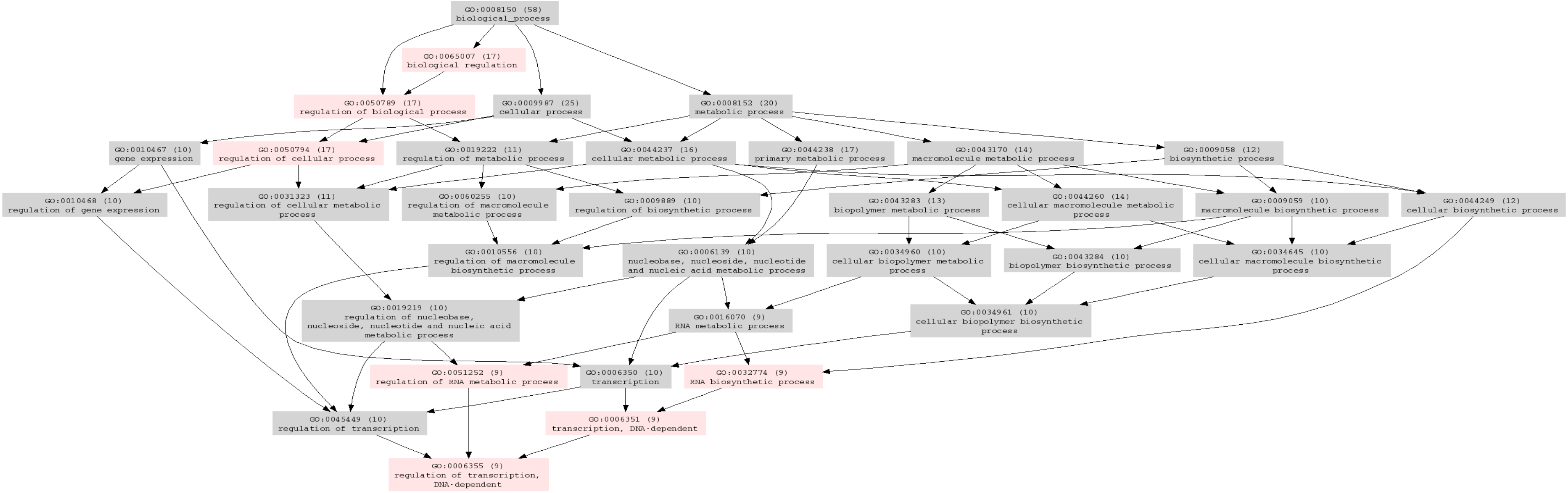

SUPPLEMENTARY DATA SET S6: Gene expression

Group F genes which were characterized by a late upregulation in the shoots

fold change

		ROOTS							SHOOTS						
		EARLY			MIDDLE		LATE		EARLY			MIDDLE		LATE	
		10m	1h	2h	4h	24h	48h	96h	10m	1h	2h	4h	24h	48h	96h
Field1	PrimaryAccession														
A_84_P21649	At1g01670.1	0.89	0.938	0.976	0.968	0.947	0.896	1.079	1.088	1.2	0.929	1.069	0.872	1.268	1.34
A_84_P18115	At1g01980.1	0.881	0.843	0.832	0.839	1.803	0.879	1.003	1.5	0.99	1.216	1.263	3.33	4.13	3.278
A_84_P55630	At1g02300.1	0.951	1.031	1.018	0.778	0.943	1.004	0.911	1.095	1.083	0.956	0.905	0.913	1.351	1.295
A_84_P220938	At1g02470.1	1.073	0.967	0.939	0.88	1.258	0.923	0.906	1.165	0.97	0.96	0.682	0.86	1.38	1.379
A_84_P507142	At1g05550.1	0.924	0.933	0.821	1.086	1.305	1.391	1.505	0.902	0.846	0.9	1.198	1.262	2.827	2.916
A_84_P18029	At1g10550.1	1.014	1.034	1.153	0.711	0.994	1.072	0.821	0.9	1.031	0.983	0.678	1.029	1.372	1.24
A_84_P21829	At1g14185.1	1.004	1.188	1.002	0.809	1.008	1.516	0.591	1.046	1.085	0.929	0.907	1.02	1.433	1.079
A_84_P21728	At1g20490.1	0.924	0.973	0.929	1.126	1.021	1.024	0.761	1.265	1.105	0.865	0.999	0.838	1.398	1.277
A_84_P537376	At1g29420.1	1.095	1.538	0.953	0.839	0.701	0.588	0.521	0.876	0.931	1.067	1.116	1.302	1.62	1.414
A_84_P523196	At1g33050.2	0.869	0.982	1.13	0.883	1.157	1.047	0.812	1.092	1.077	1.067	0.912	0.85	1.426	1.476
A_84_P17092	At1g64720.1	0.844	0.952	0.95	0.792	0.831	0.947	0.741	1.076	1.087	0.988	0.922	1.015	1.299	1.322
A_84_P297064	At1g65310.1	1.017	1.049	1.12	0.716	1.027	1.314	0.937	1.202	0.744	0.875	0.747	1.125	1.487	1.33
A_84_P531256	At1g67865.1	0.963	1.012	1.033	0.942	1.034	0.984	1.037	0.922	1.41	0.887	0.965	2.599	4.349	2.235
A_84_P21360	At1g69570.1	0.959	1.206	1.115	0.896	1.025	1.343	0.566	1.002	1.069	0.958	0.901	1.027	1.459	1.17
A_84_P17137	At1g71330.1	0.962	0.952	1.066	0.945	1.154	0.89	1.023	1.295	1.456	1.018	0.609	0.796	1.359	1.297
A_84_P15904	At1g72140.1	0.974	1.106	0.926	0.944	0.927	1.139	0.771	1.157	0.918	0.905	0.977	1.134	1.369	1.171
A_84_P156015	At1g73120.1	0.996	0.982	0.966	0.978	0.923	1.116	0.66	1.001	0.836	1.039	1.128	0.905	1.171	1.56
A_84_P16177	At1g78440.1	1.042	1.323	0.96	0.985	0.818	1.536	0.5	1.305	0.692	0.823	1.055	1.143	1.261	0.897
A_84_P21028	At2g33270.1	0.65	1.254	0.98	0.925	1.084	0.949	0.981	1.011	1.05	1.29	1.31	1.193	2.231	2.888
A_84_P11678	At2g33810.1	0.853	1.242	0.899	1.08	0.846	0.966	1.138	1.186	1.102	1.01	0.84	0.917	1.5	1.227
A_84_P22091	At3g03470.1	1.041	1.015	0.998	1.002	0.936	1.112	0.864	1.082	0.956	1.051	0.956	0.934	1.443	1.469
A_84_P511053	At3g04510.1	0.809	1.067	0.784	0.807	1.081	0.96	1.35	1.074	1.424	0.863	0.985	1.827	2.483	2.214
A_84_P12653	At3g08660.1	1.028	1.124	0.868	0.957	0.977	0.94	0.626	1.067	0.944	1.072	1.016	1.029	1.27	1.11
A_84_P18390	At3g18550.1	0.908	0.949	1.099	1.042	1.442	0.989	0.913	0.949	1.008	1.462	0.901	1.309	2.837	3.06
A_84_P20259	At3g26170.1	0.986	0.829	1.102	0.684	1.025	1.012	1.458	0.98	0.819	0.903	1.046	0.765	1.509	1.434
A_84_P522670	At3g28917.1	0.905	0.941	1.001	0.929	0.903	1.07	0.854	1.419	0.79	0.935	1.166	1.248	1.526	1.356
A_84_P538662	At3g49230.1	0.881	0.786	0.872	0.899	0.979	1.083	1.124	1.237	0.779	0.912	1.216	1.227	3.037	2.944
A_84_P16568	At3g55840.1	0.877	1.023	1.044	0.757	0.914	0.942	1.147	1.067	0.94	0.935	0.855	0.963	1.264	1.345
A_84_P11072	At4g15260.1	0.933	1.046	1.091	0.864	1.078	0.995	1.059	1.081	0.952	1.004	0.779	0.848	1.242	1.465
A_84_P260800	At4g19430.1	0.917	0.908	0.874	0.853	1.086	0.971	0.95	0.881	1.04	1.035	1	2.209	7.013	4.207
A_84_P19508	At4g23680.1	0.857	0.991	0.966	0.87	0.858	1.02	0.714	0.985	0.905	1.028	1.044	1.021	1.262	1.373
A_84_P23322	At4g30270.1	0.987	1.068	1.108	0.79	1.063	1.165	0.987	1.156	0.926	1.051	0.865	1.17	1.448	1.635
A_84_P19543	At4g31240.1	0.975	1.016	1.012	0.852	1.101	1.143	1.058	1.039	1.041	1.142	1.108	1.039	1.369	1.307
A_84_P21493	At5g02540.1	0.928	0.966	1.02	1.034	1.185	0.845	1.159	0.82	1.086	0.982	1.03	1.23	1.396	1.261
A_84_P16777	At5g07010.1	0.913	1.091	0.954	0.907	0.946	0.919	1.17	1.085	0.784	0.964	1.011	1.163	1.523	1.426
A_84_P14925	At5g23260.1	0.926	0.903	0.916	0.941	1.202	0.981	0.843	1.221	0.921	0.925	1.31	1.168	3.263	3.695
A_84_P507895	At5g43240.1	0.938	1.094	0.878	1.094	0.993	0.941	1.071	0.875	0.892	0.875	0.938	2.032	3.271	1.984
A_84_P18763	At5g45820.1	0.695	1.215	1.208	0.933	0.962	1.679	0.419	0.877	0.936	1.023	0.851	1.04	1.841	1.114
A_84_P20655	At5g45830.1	0.741	0.993	1.205	0.713	0.839	1.072	1.063	0.943	0.925	1.043	0.931	0.963	1.742	1.343
A_84_P520350	At5g55420.1	1.045	0.917	0.889	0.93	1.003	0.955	1.13	0.886	1.07	0.966	1.065	1.206	3.559	2.034
A_84_P12184	At5g56870.1	0.909	1.081	0.984	0.799	0.984	1.36	0.717	1.046	0.891	1.001	0.727	0.951	1.302	1.697
A_84_P20775	At5g58400.1	0.817	1.013	0.738	0.698	0.994	0.967	1.002	1.108	1.022	0.856	1.348	1.677	1.825	2.186
A_84_P18814	At5g59670.1	0.994	1.003	1.021	1.106	0.966	0.856	0.958	0.971	0.906	0.911	0.932	1.01	1.343	1.33
A_84_P554054	At5g60140.1	0.821	0.92	1.226	1.158	1.237	0.937	0.868	1.129	1	0.964	0.895	1.412	2.886	3.619
A_84_P506182	At5g61080.1	0.738	0.942	0.902	0.93	1.177	1.104	1.014	1.06	1.197	1.582	1.156	0.961	2.373	3.204
A_84_P15993	At5g63450.1	0.972	1.228	1.03	0.927	0.991	1.013	0.76	1.124	1.007	0.925	1.064	1.04	1.298	1.24
A_84_P23567	At5g65060.1	0.904	1.061	0.925	0.98	0.984	0.842	1.051	1.068	1.016	0.965	0.938	0.799	1.325	1.282

p value

		ROOTS							SHOOTS						
		EARLY			MIDDLE		LATE		EARLY			MIDDLE		LATE	
		10m	1h	2h	4h	24h	48h	96h	10m	1h	2h	4h	24h	48h	96h
Field1	PrimaryAccession														
A_84_P21649	At1g01670.1	0.129	0.553	0.788	0.799	0.552	0.333	0.341	0.453	0.0946	0.421	0.711	0.366	0.0303	0.0154
A_84_P18115	At1g01980.1	0.8	0.788	0.771	0.781	0.279	0.834	0.996	0.374	0.987	0.699	0.688	0.0219	0.00885	0.0313
A_84_P55630	At1g02300.1	0.493	0.719	0.813	0.133	0.655	0.964	0.56	0.299	0.321	0.615	0.421	0.366	0.00984	0.021
A_84_P220938	At1g02470.1	0.435	0.707	0.645	0.465	0.0455	0.527	0.354	0.153	0.744	0.676	0.0418	0.31	0.0115	0.0254
A_84_P507142	At1g05550.1	0.875	0.913	0.737	0.892	0.652	0.561	0.459	0.833	0.78	0.868	0.762	0.704	0.0397	0.0322
A_84_P18029	At1g10550.1	0.914	0.767	0.115	0.102	0.969	0.501	0.396	0.343	0.716	0.866	0.0029	0.8	0.0169	0.045
A_84_P21829	At1g14185.1	0.969	0.233	0.987	0.1	0.962	0.0352	0.14	0.702	0.387	0.426	0.463	0.959	0.0489	0.581
A_84_P21728	At1g20490.1	0.274	0.732	0.454	0.342	0.8	0.778	0.0288	0.045	0.381	0.193	0.998	0.176	0.0121	0.0298
A_84_P537376	At1g29420.1	0.791	0.34	0.924	0.73	0.528	0.399	0.327	0.206	0.46	0.501	0.331	0.0805	0.0187	0.0471
A_84_P523196	At1g33050.2	0.134	0.877	0.347	0.331	0.134	0.576	0.188	0.581	0.552	0.615	0.594	0.213	0.0334	0.0166
A_84_P17092	At1g64720.1	0.1	0.6	0.574	0.0841	0.19	0.6	0.169	0.252	0.306	0.917	0.355	0.948	0.0225	0.0172
A_84_P297064	At1g65310.1	0.832	0.691	0.192	0.0461	0.794	0.325	0.768	0.375	0.0837	0.41	0.0129	0.761	0.0058	0.102
A_84_P531256	At1g67865.1	0.94	0.984	0.957	0.925	0.957	0.979	0.953	0.877	0.57	0.851	0.955	0.122	0.000514	0.000482
A_84_P21360	At1g69570.1	0.566	0.141	0.202	0.421	0.784	0.0439	0.184	0.983	0.404	0.628	0.214	0.907	0.00698	0.0973
A_84_P17137	At1g71330.1	0.699	0.566	0.511	0.664	0.147	0.203	0.792	0.21	0.0401	0.899	0.0952	0.101	0.047	0.0309
A_84_P15904	At1g72140.1	0.782	0.399	0.44	0.595	0.724	0.317	0.0252	0.0879	0.425	0.366	0.789	0.731	0.0117	0.144
A_84_P156015	At1g73120.1	0.973	0.822	0.675	0.924	0.655	0.262	0.0204	0.988	0.0872	0.768	0.328	0.642	0.261	0.0275
A_84_P16177	At1g78440.1	0.664	0.0767	0.76	0.862	0.113	0.0355	0.0229	0.00888	0.104	0.0624	0.519	0.634	0.118	0.37
A_84_P21028	At2g33270.1	0.402	0.698	0.973	0.899	0.894	0.934	0.975	0.979	0.934	0.67	0.616	0.768	0.049	0.036
A_84_P11678	At2g33810.1	0.426	0.422	0.676	0.757	0.556	0.882	0.3	0.327	0.286	0.944	0.296	0.423	0.0123	0.0487
A_84_P22091	At3g03470.1	0.61	0.855	0.978	0.987	0.464	0.263	0.255	0.336	0.646	0.564	0.56	0.518	0.00733	0.00378
A_84_P511053	At3g04510.1	0.681	0.907	0.707	0.738	0.886	0.946	0.593	0.879	0.522	0.814	0.98	0.209	0.014	0.0168
A_84_P12653	At3g08660.1	0.865	0.492	0.411	0.751	0.838	0.587	0.0222	0.503	0.472	0.412	0.873	0.792	0.0286	0.265
A_84_P18390	At3g18550.1	0.836	0.934	0.875	0.947	0.539	0.986	0.881	0.912	0.99	0.504	0.864	0.666	0.0211	0.0177
A_84_P20259	At3g26170.1	0.924	0.19	0.539	0.0569	0.88	0.965	0.348	0.863	0.145	0.459	0.759	0.223	0.0127	0.0082
A_84_P522670	At3g28917.1	0.199	0.538	0.992	0.412	0.387	0.584	0.371	0.15	0.457	0.841	0.677	0.364	0.0131	0.0302
A_84_P538662	At3g49230.1	0.799	0.705	0.83	0.867	0.972	0.895	0.846	0.632	0.698	0.878	0.739	0.713	0.0311	0.0351
A_84_P16568	At3g55840.1	0.14	0.798	0.572	0.034	0.496	0.56	0.127	0.697	0.58	0.509	0.242	0.937	0.0329	0.019
A_84_P11072	At4g15260.1	0.306	0.698	0.29	0.201	0.365	0.953	0.559	0.382	0.763	0.972	0.0511	0.292	0.0447	0.00527
A_84_P260800	At4g19430.1	0.864	0.878	0.832	0.8	0.892	0.962	0.929	0.806	0.948	0.955	1	0.176	8.25E-05	0.00019
A_84_P19508	At4g23680.1	0.22	0.94	0.727	0.444	0.573	0.813	0.0908	0.88	0.301	0.797	0.798	0.833	0.029	0.0146
A_84_P23322	At4g30270.1	0.881	0.59	0.358	0.133	0.644	0.434	0.942	0.414	0.589	0.781	0.162	0.453	0.00542	0.049
A_84_P19543	At4g31240.1	0.703	0.894	0.877	0.144	0.371	0.284	0.47	0.685	0.647	0.245	0.429	0.716	0.0158	0.0399
A_84_P21493	At5g02540.1	0.547	0.721	0.872	0.773	0.135	0.306	0.294	0.173	0.409	0.87	0.857	0.401	0.00991	0.0272
A_84_P16777	At5g07010.1	0.24	0.471	0.687	0.394	0.506	0.384	0.0954	0.748	0.128	0.846	0.936	0.557	0.00759	0.0134
A_84_P14925	At5g23260.1	0.862	0.87	0.891	0.923	0.758	0.975	0.772	0.589	0.897	0.891	0.625	0.797	0.0103	0.00523
A_84_P507895	At5g43240.1	0.899	0.881	0.838	0.881	0.991	0.923	0.911	0.796	0.858	0.834	0.919	0.143	0.00992	0.035
A_84_P18763	At5g45820.1	0.0334	0.362	0.278	0.822	0.886	0.038	0.206	0.332	0.463	0.762	0.105	0.914	0.00777	0.205
A_84_P20655	At5g45830.1	0.0223	0.964	0.16	0.129	0.524	0.685	0.779	0.672	0.499	0.667	0.374	0.82	0.00204	0.0341
A_84_P520350	At5g55420.1	0.928	0.891	0.852	0.908	0.996	0.941	0.839	0.813	0.911	0.956	0.916	0.74	0.0221	0.0209
A_84_P12184	At5g56870.1	0.335	0.521	0.885	0.0461	0.889	0.26	0.431	0.812	0.429	0.996	0.012	0.869	0.11	0.0531
A_84_P20775	At5g58400.1	0.675	0.982	0.618	0.552	0.992	0.958	0.998	0.82	0.972	0.801	0.598	0.366	0.0323	0.0115
A_84_P18814	At5g59670.1	0.947	0.979	0.828	0.336	0.748	0.159	0.622	0.819	0.556	0.499	0.692	0.946	0.0147	0.0166
A_84_P554054	At5g60140.1	0.689	0.893	0.721	0.792	0.714	0.906	0.8	0.795	1	0.952	0.86	0.412	0.0104	0.00144
A_84_P506182	At5g61080.1	0.552	0.925	0.871	0.909	0.786	0.87	0.982	0.9	0.762	0.449	0.769	0.947	0.047	0.0232
A_84_P15993	At5g63450.1	0.764	0.0965	0.708	0.404	0.963	0.891	0.016	0.283	0.946	0.447	0.449	0.923	0.0175	0.315
A_84_P23567	At5g65060.1	0.193	0.451	0.397	0.79	0.894	0.146	0.525	0.412	0.831	0.646	0.51	0.143	0.013	0.0253

SUPPLEMENTARY DATA SET S6:Functional characterization

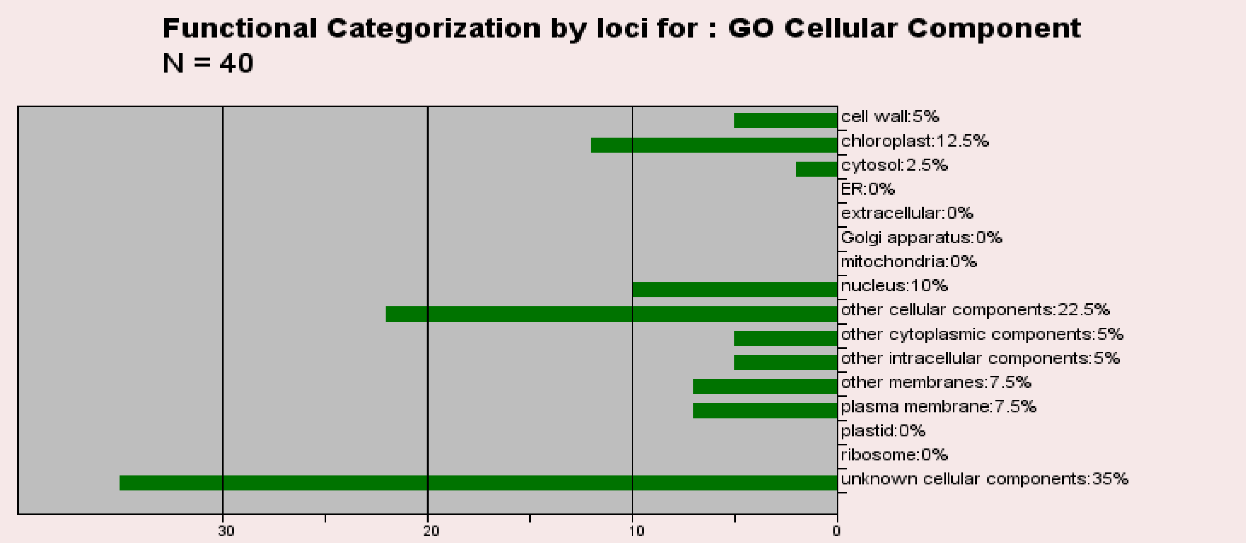

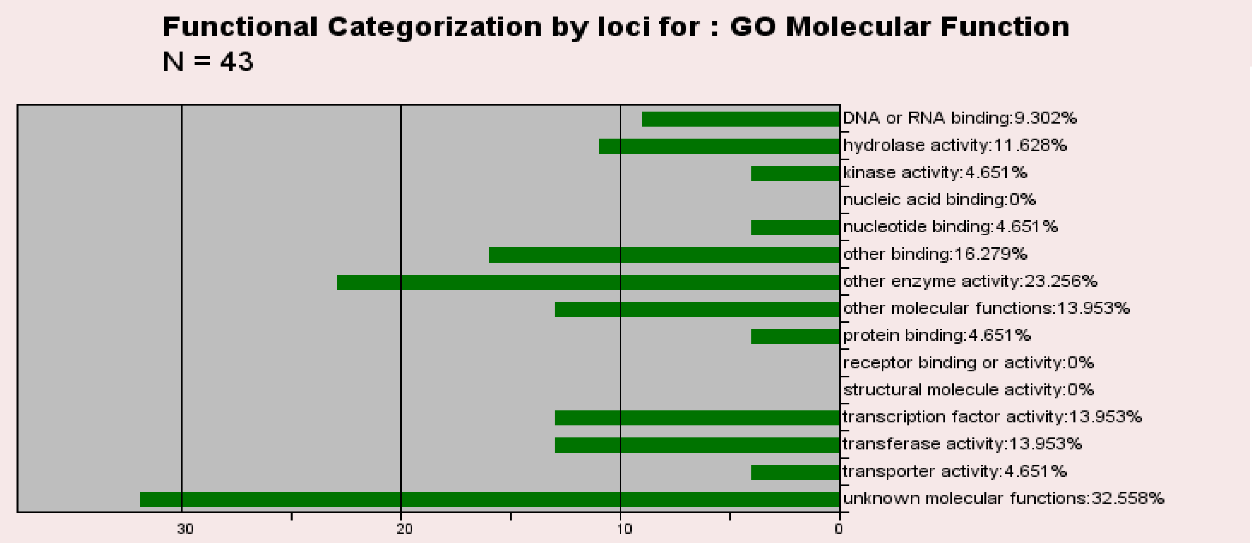

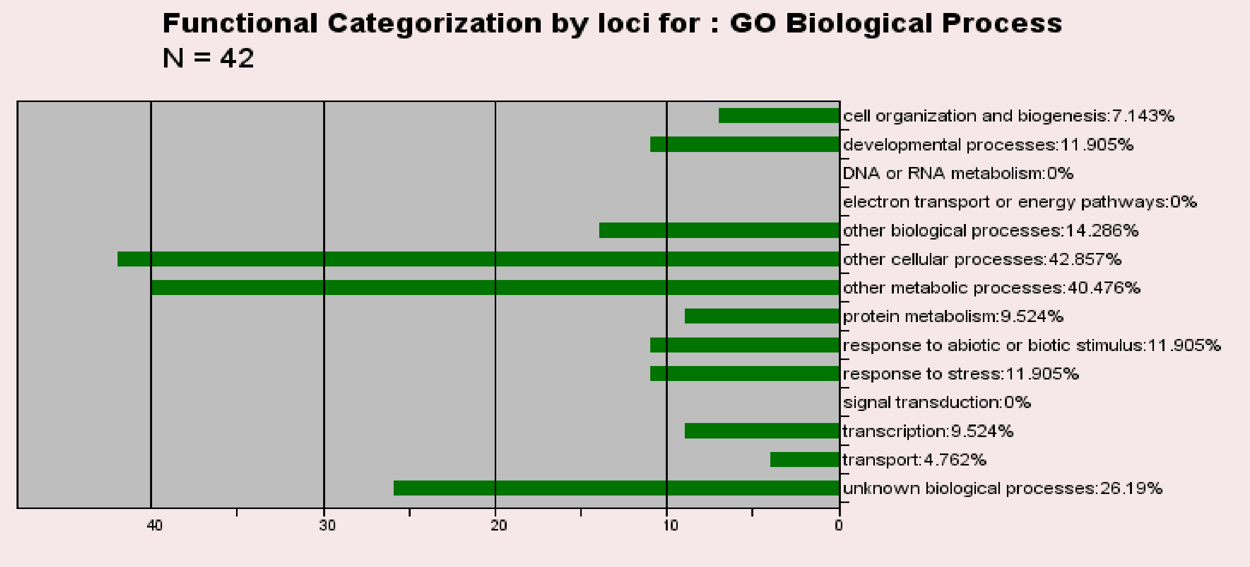

Supplementary Data Set S8 - Analysis of promoter sequences of genes responsive to a 10 min NNMF exposure showing that NAC transcription factors can be important players in the regulation of genes responsive to MFs

Regulator	Symbol	CO	PD	PWM_num	PWM_source	PWM_name	rank	p-value	q-value	#hits	hits
AT1G54330	ANAC020	0	0	PWM3357	DAP-seq	ANAC020_col	1	6.70E-11	1.20E-07	245	AT1G04450,AT1G04500,AT1G05615,AT1G07850,AT1G11990,AT1G13150,AT1G16980,AT1G17150,AT1G19460,AT1G20750,AT1G22000,AT1G23240,AT1G23520,AT1G23810,AT1G27260,AT1G27270,AT1G27860,AT1G28020,AT1G29570,AT1G29580,AT1G30670,AT1G31080,AT1G31090,AT1G32020,AT1G32980,AT1G35820,AT1G44980,AT1G47280,AT1G47625,AT1G47780,AT1G50220,AT1G51900,AT1G52460,AT1G55070,AT1G55570,AT1G56040,AT1G57906,AT1G58242,AT1G58265,AT1G58300,AT1G58430,AT1G65140,AT1G65170,AT1G65740,AT1G66960,AT1G68960,AT1G69500,AT1G70540,AT1G71290,AT1G72460,AT1G75940,AT1G76370,AT1G78160,AT1G78980,AT2G02890,AT2G05360,AT2G07020,AT2G13620,AT2G14540,AT2G14700,AT2G14830,AT2G15170,AT2G15345,AT2G15640,AT2G16140,AT2G17090,AT2G18460,AT2G18810,AT2G19420,AT2G20465,AT2G20625,AT2G21480,AT2G23660,AT2G24320,AT2G24340,AT2G25370,AT2G26850,AT2G28090,AT2G29040,AT2G29800,AT2G30290,AT2G32360,AT2G32370,AT2G33030,AT2G33090,AT2G33270,AT2G33420,AT2G34270,AT2G36700,AT2G38910,AT2G40560,AT2G42730,AT2G44550,AT2G46960,AT3G01085,AT3G02440,AT3G03405,AT3G03480,AT3G04150,AT3G06280,AT3G09450,AT3G09510,AT3G10990,AT3G11405,AT3G11480,AT3G13390,AT3G15490,AT3G18180,AT3G18810,AT3G18900,AT3G19070,AT3G19410,AT3G20750,AT3G20760,AT3G21840,AT3G23172,AT3G23245,AT3G23770,AT3G24270,AT3G24620,AT3G25650,AT3G26880,AT3G26940,AT3G28190,AT3G28980,AT3G29050,AT3G42550,AT3G43710,AT3G43750,AT3G44250,AT3G44800,AT3G44910,AT3G44920,AT3G44930,AT3G44935,AT3G45580,AT3G45760,AT3G45800,AT3G46350,AT3G46840,AT3G47190,AT3G47760,AT3G48220,AT3G49270,AT3G49305,AT3G52350,AT3G53050,AT3G53060,AT3G53790,AT3G58280,AT3G58300,AT3G58330,AT3G58410,AT3G59160,AT3G59170,AT3G59845,AT3G60060,AT3G60560,AT4G00540,AT4G01160,AT4G03620,AT4G04402,AT4G04510,AT4G04690,AT4G04985,AT4G05230,AT4G05240,AT4G10660,AT4G10680,AT4G11340,AT4G11730,AT4G12220,AT4G15200,AT4G15250,AT4G19260,AT4G19340,AT4G19800,AT4G19910,AT4G21260,AT4G21630,AT4G23882,AT4G26350,AT4G27420,AT4G28090,AT4G28395,AT4G30300,AT4G31690,AT4G32090,AT4G33840,AT4G35280,AT4G39020,AT5G01440,AT5G03580,AT5G03590,AT5G04400,AT5G05030,AT5G05150,AT5G06010,AT5G11130,AT5G12000,AT5G12180,AT5G13130,AT5G14290,AT5G16020,AT5G16090,AT5G17970,AT5G20310,AT5G22380,AT5G22900,AT5G23180,AT5G23960,AT5G25340,AT5G27310,AT5G28885,AT5G35540,AT5G36680,AT5G37320,AT5G39010,AT5G39390,AT5G39460,AT5G39470,AT5G39620,AT5G40360,AT5G41765,AT5G43390,AT5G44760,AT5G44960,AT5G48700,AT5G48750,AT5G52350,AT5G53040,AT5G53510,AT5G56110,AT5G56390,AT5G56400,AT5G56640,AT5G58170,AT5G59280,AT5G59810,AT5G60090,AT5G60740,AT5G61110,AT5G61700,AT5G61710,AT5G63720

AT3G04060	anac046	15	0	PWM3365	DAP-seq	ANAC046_col	2	4.77E-10	4.28E-07	241	AT1G04450,AT1G04500,AT1G05615,AT1G07850,AT1G11990,AT1G13150,AT1G17150,AT1G18130,AT1G19460,AT1G20730,AT1G20750,AT1G22000,AT1G23240,AT1G23520,AT1G23810,AT1G27260,AT1G27270,AT1G27860,AT1G28020,AT1G30670,AT1G31080,AT1G31090,AT1G32980,AT1G33920,AT1G35820,AT1G44980,AT1G47280,AT1G47390,AT1G47780,AT1G48590,AT1G50220,AT1G51040,AT1G51900,AT1G52460,AT1G55070,AT1G55570,AT1G56040,AT1G58265,AT1G58300,AT1G58430,AT1G58766,AT1G65140,AT1G65170,AT1G65740,AT1G66960,AT1G68040,AT1G69470,AT1G70540,AT1G70960,AT1G71290,AT1G72290,AT1G72460,AT1G75940,AT1G76290,AT1G78160,AT1G78980,AT2G02890,AT2G05360,AT2G06960,AT2G13620,AT2G14540,AT2G14830,AT2G15170,AT2G15345,AT2G17090,AT2G18460,AT2G18810,AT2G19420,AT2G20465,AT2G20625,AT2G23660,AT2G24320,AT2G24340,AT2G25370,AT2G26850,AT2G28090,AT2G29040,AT2G29800,AT2G30290,AT2G32360,AT2G32370,AT2G33030,AT2G33090,AT2G33270,AT2G33420,AT2G34270,AT2G36700,AT2G38910,AT2G40560,AT2G40955,AT2G42730,AT2G44070,AT2G44550,AT2G46960,AT3G01085,AT3G02440,AT3G03405,AT3G03480,AT3G04150,AT3G06280,AT3G09510,AT3G10595,AT3G10990,AT3G11405,AT3G11480,AT3G13390,AT3G15490,AT3G18180,AT3G18810,AT3G18900,AT3G19070,AT3G19410,AT3G20980,AT3G21840,AT3G23172,AT3G23770,AT3G23950,AT3G24270,AT3G24620,AT3G26616,AT3G26880,AT3G26940,AT3G28190,AT3G28980,AT3G43710,AT3G44250,AT3G44800,AT3G44910,AT3G44920,AT3G44930,AT3G44935,AT3G45580,AT3G45760,AT3G45800,AT3G46350,AT3G46840,AT3G47190,AT3G47760,AT3G47870,AT3G48220,AT3G48840,AT3G49270,AT3G49305,AT3G50510,AT3G52350,AT3G53050,AT3G53060,AT3G53790,AT3G56180,AT3G58280,AT3G58300,AT3G58910,AT3G59160,AT3G59170,AT3G60060,AT3G60560,AT4G00540,AT4G01160,AT4G03620,AT4G04510,AT4G04690,AT4G04985,AT4G05230,AT4G05240,AT4G10440,AT4G10660,AT4G10680,AT4G11340,AT4G12220,AT4G15200,AT4G15250,AT4G16730,AT4G19250,AT4G19260,AT4G19340,AT4G19800,AT4G19910,AT4G21630,AT4G26350,AT4G27420,AT4G30300,AT4G31690,AT4G32090,AT4G33840,AT4G34940,AT4G35280,AT4G39020,AT5G01440,AT5G04400,AT5G05030,AT5G08141,AT5G11130,AT5G11660,AT5G12000,AT5G12180,AT5G14290,AT5G15110,AT5G15480,AT5G16020,AT5G16090,AT5G17970,AT5G22900,AT5G23180,AT5G23960,AT5G24180,AT5G25340,AT5G27310,AT5G28885,AT5G35540,AT5G37320,AT5G39010,AT5G39390,AT5G39460,AT5G39470,AT5G39620,AT5G41765,AT5G43390,AT5G44345,AT5G44760,AT5G44960,AT5G48700,AT5G49750,AT5G52350,AT5G53040,AT5G53510,AT5G53600,AT5G53780,AT5G56110,AT5G56390,AT5G56400,AT5G56640,AT5G58170,AT5G59280,AT5G59810,AT5G60090,AT5G60470,AT5G60740,AT5G61110,AT5G61700,AT5G61710,AT5G63720

AT5G18270	ANAC087	35	0	PWM3388	DAP-seq	ANAC087_col	3	1.97E-09	1.17859E-06	238	AT1G04450,AT1G04500,AT1G05615,AT1G05800,AT1G07850,AT1G17150,AT1G19460,AT1G20730,AT1G20750,AT1G22000,AT1G23240,AT1G23520,AT1G23590,AT1G23810,AT1G27260,AT1G27270,AT1G27860,AT1G28020,AT1G30670,AT1G31080,AT1G31090,AT1G32020,AT1G32980,AT1G34575,AT1G35820,AT1G47280,AT1G47390,AT1G47780,AT1G48590,AT1G50220,AT1G51900,AT1G52460,AT1G55070,AT1G55570,AT1G58242,AT1G58265,AT1G58300,AT1G58430,AT1G65140,AT1G65170,AT1G65740,AT1G66960,AT1G68960,AT1G70540,AT1G70960,AT1G72290,AT1G72460,AT1G75940,AT1G76290,AT1G78160,AT1G78980,AT2G02890,AT2G05360,AT2G06960,AT2G13620,AT2G14540,AT2G14830,AT2G15170,AT2G15345,AT2G16140,AT2G17090,AT2G18460,AT2G18810,AT2G19420,AT2G20465,AT2G20625,AT2G23660,AT2G24320,AT2G24340,AT2G25370,AT2G26850,AT2G28090,AT2G29040,AT2G29800,AT2G30290,AT2G32360,AT2G32370,AT2G33030,AT2G33090,AT2G33270,AT2G33420,AT2G34270,AT2G36700,AT2G38910,AT2G39030,AT2G39640,AT2G40560,AT2G42730,AT2G44550,AT2G46960,AT3G01085,AT3G02440,AT3G03405,AT3G03480,AT3G04150,AT3G06280,AT3G09510,AT3G10595,AT3G10990,AT3G11405,AT3G11480,AT3G13390,AT3G15490,AT3G18180,AT3G18810,AT3G18900,AT3G19070,AT3G20980,AT3G21840,AT3G23172,AT3G23770,AT3G24620,AT3G25650,AT3G26880,AT3G26940,AT3G28190,AT3G28980,AT3G43710,AT3G43750,AT3G44250,AT3G44800,AT3G44910,AT3G44920,AT3G44930,AT3G44935,AT3G45580,AT3G45760,AT3G45800,AT3G46350,AT3G46840,AT3G47190,AT3G47760,AT3G48220,AT3G49270,AT3G49305,AT3G50510,AT3G52350,AT3G53050,AT3G53060,AT3G53790,AT3G56180,AT3G57250,AT3G58280,AT3G58300,AT3G58410,AT3G58910,AT3G59160,AT3G59170,AT3G60060,AT3G60560,AT4G00540,AT4G01160,AT4G03620,AT4G04510,AT4G04690,AT4G04985,AT4G05230,AT4G05240,AT4G10440,AT4G10660,AT4G10680,AT4G11340,AT4G12220,AT4G15200,AT4G15250,AT4G15650,AT4G16730,AT4G19250,AT4G19260,AT4G19340,AT4G19800,AT4G19910,AT4G21630,AT4G26350,AT4G27420,AT4G28090,AT4G28395,AT4G30300,AT4G31690,AT4G32090,AT4G33840,AT4G34940,AT4G35280,AT4G39020,AT5G03580,AT5G03590,AT5G04400,AT5G05030,AT5G05150,AT5G08141,AT5G11130,AT5G12000,AT5G12180,AT5G14290,AT5G15110,AT5G15480,AT5G16020,AT5G16090,AT5G17970,AT5G20310,AT5G22900,AT5G23180,AT5G23960,AT5G25340,AT5G26130,AT5G27310,AT5G28885,AT5G31412,AT5G35540,AT5G37320,AT5G39010,AT5G39390,AT5G39460,AT5G39470,AT5G39620,AT5G41765,AT5G43390,AT5G44960,AT5G48700,AT5G49750,AT5G52350,AT5G53040,AT5G53780,AT5G56110,AT5G56390,AT5G56400,AT5G56560,AT5G56640,AT5G58170,AT5G59280,AT5G59810,AT5G60090,AT5G60470,AT5G60740,AT5G61110,AT5G61700,AT5G61710,AT5G63720

AT2G24430	ANAC038	21	0	PWM3360	DAP-seq	ANAC038_col	4	4.49E-08	2.01257E-05	231	AT1G04450,AT1G04500,AT1G05615,AT1G07150,AT1G07473,AT1G07476,AT1G07850,AT1G11145,AT1G13290,AT1G20730,AT1G20750,AT1G22000,AT1G23240,AT1G23520,AT1G23810,AT1G26350,AT1G26797,AT1G27260,AT1G27270,AT1G27570,AT1G28020,AT1G30670,AT1G31250,AT1G32020,AT1G34575,AT1G47280,AT1G47390,AT1G47780,AT1G49800,AT1G50050,AT1G50880,AT1G51230,AT1G51900,AT1G52460,AT1G55070,AT1G55570,AT1G56040,AT1G58265,AT1G58300,AT1G58430,AT1G58766,AT1G61095,AT1G62080,AT1G65170,AT1G65740,AT1G66960,AT1G70540,AT1G70960,AT1G72290,AT1G72460,AT1G74290,AT1G75790,AT1G78160,AT1G78980,AT2G02890,AT2G05360,AT2G13620,AT2G14540,AT2G14830,AT2G15345,AT2G16140,AT2G17090,AT2G18460,AT2G18810,AT2G19420,AT2G20465,AT2G20625,AT2G22520,AT2G23660,AT2G24320,AT2G25370,AT2G26850,AT2G28090,AT2G29040,AT2G29800,AT2G30290,AT2G31030,AT2G31035,AT2G32360,AT2G33030,AT2G33270,AT2G33420,AT2G34270,AT2G36700,AT2G38910,AT2G39030,AT2G40560,AT2G40955,AT2G44550,AT2G46960,AT2G47280,AT3G01085,AT3G02440,AT3G03405,AT3G03480,AT3G04150,AT3G06090,AT3G06280,AT3G07250,AT3G09510,AT3G10990,AT3G11405,AT3G11480,AT3G13390,AT3G15490,AT3G18180,AT3G18810,AT3G18900,AT3G20980,AT3G21840,AT3G22580,AT3G23770,AT3G24620,AT3G25650,AT3G26880,AT3G26940,AT3G28190,AT3G32400,AT3G43710,AT3G44250,AT3G44805,AT3G44910,AT3G44920,AT3G44930,AT3G44935,AT3G45580,AT3G45760,AT3G45800,AT3G46840,AT3G47190,AT3G47760,AT3G48220,AT3G49270,AT3G49440,AT3G49450,AT3G52350,AT3G53050,AT3G53060,AT3G56180,AT3G56670,AT3G57850,AT3G58300,AT3G58330,AT3G59170,AT3G60060,AT3G60560,AT4G00540,AT4G01160,AT4G03450,AT4G03620,AT4G04690,AT4G05230,AT4G05240,AT4G10660,AT4G10680,AT4G11340,AT4G12220,AT4G13610,AT4G15200,AT4G15250,AT4G15650,AT4G19260,AT4G19340,AT4G21010,AT4G21630,AT4G26350,AT4G27420,AT4G29620,AT4G31690,AT4G32090,AT4G33840,AT4G34940,AT4G39020,AT5G01440,AT5G03580,AT5G03590,AT5G04400,AT5G05030,AT5G05050,AT5G08141,AT5G11130,AT5G11940,AT5G12000,AT5G12180,AT5G13130,AT5G14290,AT5G15480,AT5G16020,AT5G17970,AT5G22420,AT5G22900,AT5G23180,AT5G23960,AT5G27310,AT5G28885,AT5G29613,AT5G31412,AT5G35230,AT5G35540,AT5G35945,AT5G37320,AT5G38190,AT5G38250,AT5G38960,AT5G39010,AT5G39390,AT5G39460,AT5G39470,AT5G39620,AT5G40050,AT5G41765,AT5G43390,AT5G44630,AT5G44960,AT5G48140,AT5G48700,AT5G49260,AT5G52000,AT5G52350,AT5G53040,AT5G56110,AT5G56390,AT5G56400,AT5G56560,AT5G56640,AT5G59280,AT5G59810,AT5G60090,AT5G60830,AT5G61700,AT5G61710

AT3G10480	ANAC050	0	na	PWM3368	DAP-seq	ANAC050_col	5	4.49E-08	2.01257E-05	231	AT1G04450,AT1G04500,AT1G07850,AT1G17150,AT1G20320,AT1G20730,AT1G20750,AT1G20800,AT1G21950,AT1G22000,AT1G23240,AT1G23520,AT1G23570,AT1G23810,AT1G26350,AT1G26797,AT1G27220,AT1G27260,AT1G27270,AT1G28020,AT1G29570,AT1G29870,AT1G31080,AT1G32020,AT1G33010,AT1G33020,AT1G35820,AT1G47390,AT1G47625,AT1G47810,AT1G50880,AT1G51040,AT1G51900,AT1G58430,AT1G61095,AT1G64070,AT1G65170,AT1G65740,AT1G66960,AT1G68960,AT1G69470,AT1G70540,AT1G70960,AT1G72460,AT1G76290,AT1G78160,AT1G78980,AT2G01240,AT2G04910,AT2G04920,AT2G05360,AT2G10602,AT2G12880,AT2G13620,AT2G14830,AT2G15345,AT2G16140,AT2G17090,AT2G18810,AT2G19420,AT2G20465,AT2G20625,AT2G22520,AT2G23660,AT2G24255,AT2G24320,AT2G25370,AT2G26850,AT2G28090,AT2G29040,AT2G29800,AT2G30290,AT2G31030,AT2G31035,AT2G32360,AT2G33030,AT2G33270,AT2G33420,AT2G34270,AT2G36700,AT2G38910,AT2G40560,AT2G42730,AT2G44550,AT2G45110,AT2G46190,AT2G46960,AT2G47280,AT3G01085,AT3G03405,AT3G03480,AT3G04150,AT3G09510,AT3G10990,AT3G11405,AT3G11480,AT3G13390,AT3G15490,AT3G17500,AT3G18180,AT3G18810,AT3G18900,AT3G19410,AT3G21170,AT3G23770,AT3G23950,AT3G24620,AT3G25650,AT3G26940,AT3G27720,AT3G28190,AT3G28980,AT3G32400,AT3G33494,AT3G43710,AT3G44250,AT3G44800,AT3G44805,AT3G44910,AT3G44920,AT3G44930,AT3G44935,AT3G45760,AT3G45800,AT3G46240,AT3G46840,AT3G47870,AT3G48220,AT3G49270,AT3G49305,AT3G52350,AT3G53050,AT3G53060,AT3G53790,AT3G56180,AT3G56670,AT3G58280,AT3G58300,AT3G58330,AT3G58410,AT3G59160,AT3G59170,AT3G60060,AT4G00540,AT4G01985,AT4G03380,AT4G03620,AT4G04690,AT4G08097,AT4G08395,AT4G10660,AT4G11340,AT4G12220,AT4G13610,AT4G14260,AT4G15200,AT4G15250,AT4G15650,AT4G19250,AT4G19260,AT4G19340,AT4G20810,AT4G21010,AT4G21630,AT4G23370,AT4G26350,AT4G27420,AT4G29970,AT4G30300,AT4G31690,AT4G32090,AT4G38120,AT4G39020,AT4G39745,AT5G01440,AT5G01680,AT5G02730,AT5G03580,AT5G03590,AT5G04400,AT5G05030,AT5G05150,AT5G08141,AT5G11130,AT5G12000,AT5G13130,AT5G15480,AT5G16020,AT5G16090,AT5G17970,AT5G19610,AT5G22900,AT5G23180,AT5G23260,AT5G23960,AT5G27310,AT5G28885,AT5G31412,AT5G35540,AT5G37320,AT5G38960,AT5G39010,AT5G39390,AT5G39620,AT5G40050,AT5G40750,AT5G41765,AT5G43390,AT5G47290,AT5G47300,AT5G48140,AT5G48700,AT5G49070,AT5G49750,AT5G52350,AT5G52610,AT5G53040,AT5G53780,AT5G56110,AT5G56390,AT5G56400,AT5G59810,AT5G60090,AT5G60470,AT5G60500,AT5G60740,AT5G60830,AT5G61110,AT5G61700,AT5G61710,AT5G62060

AT5G07680	ANAC079	21	0	PWM3384	DAP-seq	ANAC079_col	6	1.58E-07	4.7285E-05	228	AT1G04450,AT1G04500,AT1G05615,AT1G05800,AT1G07850,AT1G11770,AT1G11990,AT1G13150,AT1G17150,AT1G19460,AT1G20750,AT1G22000,AT1G23240,AT1G23520,AT1G23590,AT1G23810,AT1G27260,AT1G27270,AT1G27860,AT1G28020,AT1G30670,AT1G31080,AT1G32980,AT1G35820,AT1G47280,AT1G47390,AT1G47780,AT1G48590,AT1G50220,AT1G51900,AT1G52460,AT1G55070,AT1G55570,AT1G56040,AT1G58242,AT1G58265,AT1G58300,AT1G58430,AT1G61095,AT1G63280,AT1G65140,AT1G65170,AT1G65740,AT1G66960,AT1G69500,AT1G70540,AT1G70960,AT1G72290,AT1G72460,AT1G75940,AT1G78160,AT1G78980,AT2G02890,AT2G05360,AT2G13620,AT2G14540,AT2G14700,AT2G14830,AT2G15170,AT2G15345,AT2G15640,AT2G16140,AT2G17090,AT2G18460,AT2G18810,AT2G19420,AT2G20465,AT2G20625,AT2G23660,AT2G24320,AT2G25370,AT2G26850,AT2G28090,AT2G29040,AT2G29800,AT2G30290,AT2G32360,AT2G32370,AT2G33090,AT2G33270,AT2G33420,AT2G34120,AT2G34270,AT2G36700,AT2G38910,AT2G39030,AT2G40560,AT2G42730,AT2G44550,AT2G46960,AT3G01085,AT3G02440,AT3G03405,AT3G03480,AT3G04150,AT3G06280,AT3G06630,AT3G09510,AT3G10990,AT3G11405,AT3G11480,AT3G13390,AT3G15490,AT3G18180,AT3G18810,AT3G18900,AT3G19070,AT3G19410,AT3G21840,AT3G23480,AT3G23770,AT3G24270,AT3G24620,AT3G26880,AT3G26940,AT3G28190,AT3G28980,AT3G43710,AT3G43750,AT3G44250,AT3G44800,AT3G44910,AT3G44920,AT3G44930,AT3G44935,AT3G45580,AT3G45760,AT3G46350,AT3G46840,AT3G47190,AT3G47760,AT3G48220,AT3G49270,AT3G49305,AT3G52350,AT3G53050,AT3G53060,AT3G58280,AT3G58300,AT3G58330,AT3G59160,AT3G59170,AT3G60060,AT3G60560,AT4G00540,AT4G01160,AT4G03620,AT4G04510,AT4G04690,AT4G04985,AT4G05230,AT4G05240,AT4G10660,AT4G10680,AT4G11340,AT4G11730,AT4G12220,AT4G15200,AT4G15250,AT4G16730,AT4G19250,AT4G19260,AT4G19340,AT4G19910,AT4G21240,AT4G21630,AT4G26350,AT4G27420,AT4G28090,AT4G28395,AT4G30300,AT4G31690,AT4G32090,AT4G33840,AT4G34940,AT4G35280,AT4G39020,AT5G04400,AT5G05030,AT5G05150,AT5G11130,AT5G12000,AT5G12180,AT5G14290,AT5G15480,AT5G16020,AT5G16090,AT5G17970,AT5G20310,AT5G23180,AT5G23960,AT5G25340,AT5G26130,AT5G27310,AT5G28410,AT5G28885,AT5G35540,AT5G36680,AT5G37320,AT5G39010,AT5G39390,AT5G39460,AT5G39470,AT5G39620,AT5G39770,AT5G40360,AT5G41765,AT5G43390,AT5G44630,AT5G44760,AT5G44960,AT5G48700,AT5G52350,AT5G53040,AT5G53510,AT5G56110,AT5G56390,AT5G56400,AT5G56640,AT5G58170,AT5G59280,AT5G59810,AT5G60090,AT5G60470,AT5G60740,AT5G61700,AT5G61710,AT5G63720

AT1G34190	anac017	0	0	PWM3355	DAP-seq	ANAC017_col	7	5.31E-07	0.000136092	225	AT1G04450,AT1G04500,AT1G05615,AT1G07150,AT1G07850,AT1G11990,AT1G13150,AT1G17150,AT1G17800,AT1G20320,AT1G20730,AT1G20750,AT1G20800,AT1G22000,AT1G23240,AT1G23520,AT1G23570,AT1G23810,AT1G26350,AT1G27220,AT1G27260,AT1G27270,AT1G29570,AT1G29580,AT1G31080,AT1G32020,AT1G33020,AT1G35400,AT1G35820,AT1G35900,AT1G36960,AT1G46408,AT1G47390,AT1G47810,AT1G49800,AT1G51040,AT1G51230,AT1G51900,AT1G52460,AT1G58430,AT1G60350,AT1G61095,AT1G65140,AT1G65170,AT1G65740,AT1G66960,AT1G68960,AT1G70540,AT1G72460,AT1G74220,AT1G78980,AT2G01240,AT2G05360,AT2G07280,AT2G10602,AT2G13620,AT2G14540,AT2G14830,AT2G16450,AT2G17090,AT2G18810,AT2G19420,AT2G20625,AT2G22520,AT2G23660,AT2G24255,AT2G28090,AT2G29040,AT2G29800,AT2G30290,AT2G31030,AT2G31035,AT2G31760,AT2G32360,AT2G32370,AT2G33030,AT2G33270,AT2G33420,AT2G34270,AT2G40560,AT2G42730,AT2G44550,AT2G46190,AT2G46960,AT2G47280,AT2G47660,AT3G01085,AT3G03480,AT3G04150,AT3G09110,AT3G09510,AT3G10990,AT3G11405,AT3G11480,AT3G13390,AT3G15490,AT3G17500,AT3G18180,AT3G18810,AT3G18900,AT3G20980,AT3G23770,AT3G23950,AT3G24620,AT3G25650,AT3G26390,AT3G26940,AT3G27720,AT3G29050,AT3G30220,AT3G30770,AT3G32400,AT3G33494,AT3G43710,AT3G44250,AT3G44910,AT3G44920,AT3G44930,AT3G44935,AT3G45580,AT3G45760,AT3G45800,AT3G46310,AT3G46840,AT3G47760,AT3G49270,AT3G49305,AT3G52350,AT3G53050,AT3G53060,AT3G56670,AT3G58280,AT3G58300,AT3G58330,AT3G59845,AT3G59960,AT3G60060,AT4G00540,AT4G01985,AT4G03380,AT4G03620,AT4G04402,AT4G04690,AT4G06479,AT4G08097,AT4G08395,AT4G10440,AT4G10660,AT4G10680,AT4G11340,AT4G12220,AT4G13610,AT4G14260,AT4G15200,AT4G15250,AT4G15650,AT4G19250,AT4G19260,AT4G19340,AT4G20790,AT4G21630,AT4G21690,AT4G26350,AT4G27420,AT4G29970,AT4G30300,AT4G32090,AT4G35210,AT4G38070,AT4G38120,AT4G39020,AT4G39745,AT5G01130,AT5G01440,AT5G01680,AT5G03580,AT5G03590,AT5G03980,AT5G04400,AT5G05030,AT5G05150,AT5G08141,AT5G11130,AT5G12000,AT5G12180,AT5G13130,AT5G16020,AT5G16090,AT5G17750,AT5G17970,AT5G22900,AT5G23260,AT5G23960,AT5G24180,AT5G25340,AT5G28885,AT5G35230,AT5G35540,AT5G37320,AT5G39010,AT5G39460,AT5G40050,AT5G41765,AT5G43390,AT5G44960,AT5G47290,AT5G47300,AT5G48140,AT5G49070,AT5G49260,AT5G49750,AT5G52350,AT5G53040,AT5G53510,AT5G53600,AT5G53780,AT5G56110,AT5G56390,AT5G56400,AT5G59810,AT5G60090,AT5G60740,AT5G61700,AT5G61710,AT5G63720

AT3G15510	ANAC056	22	0	PWM3406	DAP-seq	NAC2_col	8	7.87E-07	0.0001764	224	AT1G04450,AT1G04500,AT1G07150,AT1G07850,AT1G11990,AT1G13150,AT1G17150,AT1G20730,AT1G20750,AT1G20800,AT1G22000,AT1G23240,AT1G23520,AT1G23810,AT1G26350,AT1G27220,AT1G27260,AT1G27270,AT1G29570,AT1G29580,AT1G31080,AT1G32020,AT1G32980,AT1G33010,AT1G33020,AT1G35820,AT1G36960,AT1G46408,AT1G46984,AT1G51040,AT1G51230,AT1G51900,AT1G55570,AT1G57906,AT1G58430,AT1G60240,AT1G60350,AT1G60830,AT1G61095,AT1G65170,AT1G65740,AT1G66960,AT1G68960,AT1G69100,AT1G72290,AT1G74220,AT1G76370,AT1G78160,AT1G78980,AT2G01240,AT2G02890,AT2G04870,AT2G05360,AT2G13620,AT2G14830,AT2G15170,AT2G15345,AT2G15640,AT2G16450,AT2G17090,AT2G17750,AT2G19420,AT2G20625,AT2G22520,AT2G23660,AT2G24255,AT2G24320,AT2G28090,AT2G29040,AT2G29800,AT2G30290,AT2G30730,AT2G32360,AT2G33030,AT2G33270,AT2G33420,AT2G34270,AT2G40560,AT2G42730,AT2G43730,AT2G44175,AT2G44550,AT2G46190,AT2G46960,AT2G47280,AT3G01085,AT3G03405,AT3G04150,AT3G09510,AT3G10990,AT3G11405,AT3G11480,AT3G12000,AT3G13390,AT3G15490,AT3G17500,AT3G18180,AT3G18810,AT3G18900,AT3G19410,AT3G20155,AT3G21880,AT3G23770,AT3G23850,AT3G23950,AT3G24620,AT3G25650,AT3G26390,AT3G26880,AT3G26940,AT3G27720,AT3G28190,AT3G30770,AT3G43710,AT3G44250,AT3G44805,AT3G44910,AT3G44920,AT3G44930,AT3G44935,AT3G45580,AT3G45760,AT3G45800,AT3G46840,AT3G47190,AT3G47760,AT3G48220,AT3G49270,AT3G49305,AT3G52350,AT3G53050,AT3G53060,AT3G58280,AT3G58300,AT3G58330,AT3G59170,AT3G59180,AT3G60060,AT3G60920,AT4G00540,AT4G03620,AT4G04690,AT4G04985,AT4G06479,AT4G08097,AT4G10680,AT4G11340,AT4G11730,AT4G12220,AT4G13610,AT4G14260,AT4G15200,AT4G15250,AT4G15650,AT4G19250,AT4G19260,AT4G19340,AT4G19800,AT4G20790,AT4G21630,AT4G26350,AT4G27420,AT4G28090,AT4G30300,AT4G32090,AT4G35210,AT4G35820,AT4G38120,AT4G39020,AT4G39745,AT5G01440,AT5G03580,AT5G03590,AT5G04045,AT5G04400,AT5G05030,AT5G05150,AT5G08141,AT5G11130,AT5G12000,AT5G12180,AT5G13130,AT5G16020,AT5G16090,AT5G17180,AT5G17750,AT5G17970,AT5G19610,AT5G22900,AT5G23260,AT5G23960,AT5G25340,AT5G25390,AT5G27310,AT5G28885,AT5G31412,AT5G35540,AT5G36680,AT5G37320,AT5G39010,AT5G39620,AT5G40050,AT5G41765,AT5G43390,AT5G44760,AT5G48140,AT5G49260,AT5G49750,AT5G52350,AT5G53040,AT5G53510,AT5G53780,AT5G56110,AT5G56390,AT5G56400,AT5G56640,AT5G59810,AT5G60090,AT5G60740,AT5G60830,AT5G61110,AT5G61700,AT5G61710,AT5G63720

AT3G18400	anac058	22	0	PWM3375	DAP-seq	ANAC058_col	9	1.15961E-06	0.00023102	223	AT1G04450,AT1G04500,AT1G07150,AT1G07850,AT1G13290,AT1G20730,AT1G20750,AT1G20800,AT1G22000,AT1G23240,AT1G23520,AT1G23810,AT1G26350,AT1G26797,AT1G27260,AT1G27270,AT1G28000,AT1G28020,AT1G30670,AT1G32020,AT1G33020,AT1G35820,AT1G47780,AT1G51040,AT1G51230,AT1G51900,AT1G52460,AT1G55070,AT1G58265,AT1G58300,AT1G58430,AT1G65140,AT1G65170,AT1G66960,AT1G70540,AT1G70960,AT1G72290,AT1G72460,AT1G74290,AT1G75790,AT1G78160,AT1G78980,AT2G02890,AT2G04910,AT2G05360,AT2G13620,AT2G14540,AT2G14830,AT2G15345,AT2G17090,AT2G18810,AT2G19420,AT2G20465,AT2G20625,AT2G23660,AT2G24255,AT2G24320,AT2G25370,AT2G26850,AT2G28090,AT2G29040,AT2G29800,AT2G30290,AT2G31030,AT2G31035,AT2G32360,AT2G33270,AT2G33420,AT2G34270,AT2G36260,AT2G36700,AT2G38910,AT2G40560,AT2G42730,AT2G44550,AT2G46190,AT2G46960,AT2G47280,AT3G01085,AT3G03405,AT3G03480,AT3G04150,AT3G06090,AT3G06280,AT3G07250,AT3G09510,AT3G10990,AT3G11405,AT3G11480,AT3G13390,AT3G15490,AT3G17500,AT3G18180,AT3G18810,AT3G18900,AT3G20155,AT3G20980,AT3G21840,AT3G23770,AT3G24620,AT3G25650,AT3G26940,AT3G28190,AT3G28980,AT3G32400,AT3G33494,AT3G43710,AT3G44250,AT3G44800,AT3G44805,AT3G44910,AT3G44920,AT3G44930,AT3G44935,AT3G45580,AT3G45760,AT3G45800,AT3G46240,AT3G46840,AT3G47760,AT3G47870,AT3G48220,AT3G49270,AT3G49440,AT3G49450,AT3G52350,AT3G53050,AT3G53060,AT3G56180,AT3G56670,AT3G57850,AT3G58280,AT3G58300,AT3G58330,AT3G59170,AT3G59845,AT3G60060,AT4G00540,AT4G01160,AT4G03620,AT4G04510,AT4G04690,AT4G05230,AT4G05240,AT4G08097,AT4G08395,AT4G10660,AT4G11340,AT4G12220,AT4G13610,AT4G15200,AT4G15250,AT4G15650,AT4G19260,AT4G19340,AT4G21010,AT4G21630,AT4G26350,AT4G27420,AT4G31690,AT4G32090,AT4G33840,AT4G39020,AT5G01440,AT5G01680,AT5G02730,AT5G03580,AT5G03590,AT5G04400,AT5G05030,AT5G08141,AT5G11130,AT5G11940,AT5G12000,AT5G12180,AT5G13130,AT5G14290,AT5G15480,AT5G16020,AT5G16090,AT5G17970,AT5G19610,AT5G22900,AT5G23180,AT5G23260,AT5G23960,AT5G27310,AT5G28885,AT5G31412,AT5G35230,AT5G35540,AT5G37320,AT5G38960,AT5G39010,AT5G39390,AT5G39460,AT5G39470,AT5G39620,AT5G40050,AT5G41765,AT5G43390,AT5G44960,AT5G47290,AT5G47300,AT5G48140,AT5G48700,AT5G49260,AT5G52350,AT5G52610,AT5G53040,AT5G56110,AT5G56390,AT5G56400,AT5G56560,AT5G56640,AT5G59280,AT5G59810,AT5G60090,AT5G60470,AT5G60740,AT5G60830,AT5G61700,AT5G61710

AT5G64060	anac103	4	0	PWM3395	DAP-seq	ANAC103_col	10	2.47643E-06	0.000444023	221	AT5G45690,AT1G04450,AT1G04500,AT1G05615,AT1G05800,AT1G07850,AT1G11990,AT1G17150,AT1G18130,AT1G20750,AT1G20800,AT1G21950,AT1G22000,AT1G23420,AT1G23570,AT1G27260,AT1G27270,AT1G29570,AT1G29870,AT1G30670,AT1G31080,AT1G31090,AT1G31620,AT1G31740,AT1G32020,AT1G33020,AT1G33880,AT1G35820,AT1G46984,AT1G47625,AT1G47780,AT1G50220,AT1G51040,AT1G51900,AT1G52460,AT1G52970,AT1G53420,AT1G53815,AT1G53820,AT1G55070,AT1G55570,AT1G56040,AT1G57906,AT1G58265,AT1G58300,AT1G58430,AT1G61095,AT1G65140,AT1G65170,AT1G69100,AT1G70540,AT1G72460,AT1G78160,AT1G78220,AT1G78980,AT2G01240,AT2G02890,AT2G06645,AT2G07140,AT2G14440,AT2G15460,AT2G15470,AT2G15640,AT2G15710,AT2G17090,AT2G17750,AT2G18810,AT2G19420,AT2G20465,AT2G20625,AT2G23660,AT2G24255,AT2G24320,AT2G25370,AT2G26850,AT2G28090,AT2G29800,AT2G30290,AT2G31030,AT2G31035,AT2G32370,AT2G33030,AT2G33420,AT2G34270,AT2G36700,AT2G38910,AT2G41920,AT2G42730,AT2G44550,AT2G46190,AT2G46460,AT2G46960,AT3G03480,AT3G04150,AT3G06280,AT3G09450,AT3G09510,AT3G10585,AT3G10595,AT3G11405,AT3G11480,AT3G13390,AT3G16680,AT3G18810,AT3G18900,AT3G19410,AT3G21170,AT3G22030,AT3G23770,AT3G23950,AT3G24220,AT3G24620,AT3G25650,AT3G26390,AT3G26880,AT3G26940,AT3G28980,AT3G32400,AT3G42550,AT3G42786,AT3G43260,AT3G43710,AT3G43750,AT3G44250,AT3G44800,AT3G44910,AT3G44920,AT3G44930,AT3G45580,AT3G45800,AT3G46840,AT3G47760,AT3G49270,AT3G52350,AT3G53050,AT3G53060,AT3G53790,AT3G56180,AT3G58330,AT3G59160,AT3G59170,AT3G60060,AT3G60560,AT4G00540,AT4G01985,AT4G03620,AT4G04690,AT4G05370,AT4G09190,AT4G10660,AT4G11340,AT4G12220,AT4G13610,AT4G15250,AT4G17590,AT4G18330,AT4G19250,AT4G19260,AT4G19340,AT4G20790,AT4G21010,AT4G21260,AT4G21630,AT4G26350,AT4G27420,AT4G30300,AT4G30770,AT4G31690,AT4G38120,AT4G39020,AT4G39745,AT5G02730,AT5G08141,AT5G12000,AT5G15110,AT5G15480,AT5G16020,AT5G16090,AT5G17180,AT5G17730,AT5G17740,AT5G22380,AT5G22900,AT5G23180,AT5G23260,AT5G23960,AT5G25340,AT5G27310,AT5G27790,AT5G28550,AT5G28950,AT5G29613,AT5G35715,AT5G38960,AT5G39010,AT5G39620,AT5G39630,AT5G40050,AT5G41500,AT5G41765,AT5G43390,AT5G44760,AT5G46990,AT5G47290,AT5G47300,AT5G48700,AT5G52350,AT5G52610,AT5G52770,AT5G53040,AT5G55080,AT5G56330,AT5G56390,AT5G56400,AT5G56560,AT5G58170,AT5G60090,AT5G60830,AT5G61605,AT5G61690,AT5G61700

AT3G15170	ANAC054	9	0	PWM3400	DAP-seq	CUC1_col	11	3.58945E-06	0.00058508	220	AT1G04450,AT1G04500,AT1G06280,AT1G07850,AT1G13150,AT1G19460,AT1G20320,AT1G20730,AT1G20750,AT1G20800,AT1G22000,AT1G23240,AT1G23520,AT1G23810,AT1G26350,AT1G26797,AT1G27220,AT1G27260,AT1G27270,AT1G29570,AT1G30670,AT1G32020,AT1G35820,AT1G36510,AT1G46408,AT1G47280,AT1G47780,AT1G51040,AT1G51230,AT1G51900,AT1G52460,AT1G58300,AT1G58430,AT1G59630,AT1G65140,AT1G65170,AT1G65740,AT1G66960,AT1G68960,AT1G70540,AT1G70960,AT1G72290,AT1G72460,AT1G75940,AT1G78160,AT1G78980,AT2G02890,AT2G04910,AT2G04920,AT2G05360,AT2G05970,AT2G06960,AT2G13620,AT2G14540,AT2G14830,AT2G15170,AT2G15345,AT2G16450,AT2G17090,AT2G18810,AT2G19420,AT2G20465,AT2G20625,AT2G23660,AT2G24320,AT2G24340,AT2G26850,AT2G28090,AT2G29040,AT2G29800,AT2G30290,AT2G31030,AT2G31035,AT2G32360,AT2G33030,AT2G33270,AT2G33420,AT2G34270,AT2G36700,AT2G39640,AT2G40560,AT2G42730,AT2G44550,AT3G01085,AT3G03405,AT3G03480,AT3G04150,AT3G06280,AT3G09510,AT3G10990,AT3G11405,AT3G11480,AT3G13390,AT3G15490,AT3G17500,AT3G18180,AT3G18810,AT3G18900,AT3G20155,AT3G20980,AT3G21840,AT3G23770,AT3G24620,AT3G25650,AT3G26880,AT3G26940,AT3G28190,AT3G28980,AT3G32400,AT3G33494,AT3G43710,AT3G44250,AT3G44910,AT3G44920,AT3G44930,AT3G44935,AT3G45580,AT3G45760,AT3G45800,AT3G46240,AT3G46840,AT3G47190,AT3G47760,AT3G47870,AT3G49270,AT3G49305,AT3G52330,AT3G52350,AT3G53050,AT3G53060,AT3G56670,AT3G58280,AT3G58300,AT3G58410,AT3G58890,AT3G59170,AT3G59845,AT3G60060,AT4G00540,AT4G01160,AT4G03620,AT4G04510,AT4G04690,AT4G08097,AT4G08395,AT4G10660,AT4G11340,AT4G12220,AT4G13610,AT4G13885,AT4G14260,AT4G15200,AT4G15250,AT4G15650,AT4G16730,AT4G19260,AT4G19340,AT4G19800,AT4G21630,AT4G26350,AT4G27420,AT4G28090,AT4G32090,AT4G33840,AT4G38070,AT4G38120,AT4G39020,AT5G01440,AT5G03580,AT5G03590,AT5G04400,AT5G05030,AT5G08141,AT5G11130,AT5G11940,AT5G12000,AT5G12180,AT5G13130,AT5G14290,AT5G15480,AT5G16020,AT5G16090,AT5G17740,AT5G17750,AT5G17970,AT5G23180,AT5G23260,AT5G23790,AT5G23960,AT5G27310,AT5G28885,AT5G31412,AT5G35540,AT5G37320,AT5G39010,AT5G39390,AT5G39460,AT5G39470,AT5G39620,AT5G40050,AT5G41765,AT5G43390,AT5G44960,AT5G48140,AT5G48700,AT5G52350,AT5G53040,AT5G56070,AT5G56110,AT5G56390,AT5G56400,AT5G56640,AT5G58170,AT5G59280,AT5G59810,AT5G60090,AT5G60740,AT5G60830,AT5G61700,AT5G61710

AT1G32510	ANAC011	168	na	PWM3350	DAP-seq	ANAC011_col	12	1.05766E-05	0.001580324	217	AT3G22640,AT1G04450,AT1G04500,AT1G05615,AT1G05800,AT1G07850,AT1G11990,AT1G13150,AT1G17150,AT1G20240,AT1G20750,AT1G22000,AT1G23240,AT1G23270,AT1G23520,AT1G23810,AT1G27260,AT1G27270,AT1G27860,AT1G29570,AT1G29580,AT1G29870,AT1G30670,AT1G31080,AT1G32980,AT1G33010,AT1G44980,AT1G47280,AT1G51040,AT1G51900,AT1G53420,AT1G55570,AT1G60240,AT1G63070,AT1G65170,AT1G65740,AT1G66960,AT1G68960,AT1G69470,AT1G69500,AT1G70110,AT1G70120,AT1G70540,AT1G72460,AT1G74220,AT1G76370,AT1G78160,AT1G78980,AT2G02890,AT2G13620,AT2G14700,AT2G14830,AT2G15345,AT2G15610,AT2G15640,AT2G16140,AT2G17090,AT2G18460,AT2G18810,AT2G19420,AT2G20625,AT2G21480,AT2G22520,AT2G23660,AT2G24320,AT2G28090,AT2G29040,AT2G29800,AT2G30290,AT2G32360,AT2G33030,AT2G33090,AT2G33270,AT2G33420,AT2G34270,AT2G39030,AT2G40500,AT2G40560,AT2G42730,AT2G43730,AT2G44550,AT2G46960,AT3G01085,AT3G02440,AT3G04150,AT3G04280,AT3G07250,AT3G09510,AT3G10990,AT3G11405,AT3G11480,AT3G13390,AT3G16680,AT3G17500,AT3G18180,AT3G18810,AT3G18900,AT3G19070,AT3G19410,AT3G20750,AT3G20760,AT3G21880,AT3G23770,AT3G23960,AT3G24270,AT3G24620,AT3G25650,AT3G26616,AT3G26880,AT3G28190,AT3G28810,AT3G28980,AT3G32050,AT3G43710,AT3G44250,AT3G44805,AT3G44910,AT3G44920,AT3G44930,AT3G44935,AT3G45580,AT3G45760,AT3G45800,AT3G46350,AT3G46840,AT3G47190,AT3G49270,AT3G49305,AT3G52350,AT3G53050,AT3G53060,AT3G58280,AT3G58300,AT3G58360,AT3G58390,AT3G58410,AT3G59845,AT3G60060,AT4G00540,AT4G01160,AT4G03620,AT4G04402,AT4G04510,AT4G04985,AT4G08430,AT4G09860,AT4G10660,AT4G10680,AT4G11340,AT4G11730,AT4G15200,AT4G15250,AT4G18330,AT4G19260,AT4G19340,AT4G19910,AT4G21630,AT4G26350,AT4G27420,AT4G28090,AT4G28395,AT4G29460,AT4G30300,AT4G32090,AT4G35210,AT5G04400,AT5G05030,AT5G05150,AT5G11130,AT5G12000,AT5G12180,AT5G13130,AT5G16020,AT5G16090,AT5G17740,AT5G17750,AT5G17970,AT5G19610,AT5G20310,AT5G23180,AT5G23960,AT5G27310,AT5G28885,AT5G35540,AT5G36680,AT5G36980,AT5G37320,AT5G38440,AT5G38920,AT5G39390,AT5G39620,AT5G40050,AT5G43390,AT5G44960,AT5G48700,AT5G48750,AT5G49260,AT5G51860,AT5G52000,AT5G52160,AT5G52350,AT5G53040,AT5G53510,AT5G53780,AT5G56110,AT5G56390,AT5G56400,AT5G56640,AT5G59810,AT5G60090,AT5G60500,AT5G60740,AT5G61110,AT5G61700,AT5G61710,AT5G63720,AT5G63740

AT5G53950	ANAC098	5	0	PWM3402	DAP-seq	CUC2_col	13	1.49971E-05	0.002068449	216	AT1G04450,AT1G04500,AT1G05800,AT1G07850,AT1G10100,AT1G11990,AT1G19460,AT1G20320,AT1G20730,AT1G20750,AT1G22000,AT1G23240,AT1G23520,AT1G23810,AT1G26350,AT1G26797,AT1G27220,AT1G27260,AT1G27270,AT1G28020,AT1G29570,AT1G29580,AT1G30670,AT1G32980,AT1G36510,AT1G36960,AT1G46408,AT1G47280,AT1G47780,AT1G51040,AT1G51230,AT1G51900,AT1G52460,AT1G53940,AT1G58265,AT1G58300,AT1G58430,AT1G59630,AT1G65140,AT1G65170,AT1G65740,AT1G66960,AT1G69470,AT1G70540,AT1G72460,AT1G75940,AT1G78980,AT2G02890,AT2G04910,AT2G04920,AT2G05360,AT2G13620,AT2G14540,AT2G14830,AT2G15345,AT2G17090,AT2G18810,AT2G19420,AT2G20465,AT2G20625,AT2G23660,AT2G24320,AT2G26850,AT2G28090,AT2G29040,AT2G29800,AT2G30290,AT2G32360,AT2G32370,AT2G33090,AT2G33270,AT2G33420,AT2G34270,AT2G36700,AT2G38910,AT2G40560,AT2G42730,AT2G44070,AT2G44550,AT2G46960,AT3G01085,AT3G03405,AT3G03480,AT3G04150,AT3G04270,AT3G06280,AT3G09510,AT3G10595,AT3G10990,AT3G11405,AT3G11480,AT3G11920,AT3G13390,AT3G15490,AT3G17500,AT3G18180,AT3G18810,AT3G18900,AT3G19410,AT3G20980,AT3G21840,AT3G23770,AT3G24620,AT3G26880,AT3G26940,AT3G28980,AT3G32400,AT3G33494,AT3G43710,AT3G44250,AT3G44910,AT3G44920,AT3G44930,AT3G44935,AT3G45760,AT3G46310,AT3G46840,AT3G47190,AT3G47760,AT3G48220,AT3G49270,AT3G49305,AT3G52350,AT3G53050,AT3G53060,AT3G56180,AT3G56670,AT3G58300,AT3G58890,AT3G59160,AT3G59170,AT3G59845,AT3G60060,AT4G00540,AT4G01160,AT4G03380,AT4G03620,AT4G04510,AT4G04690,AT4G04985,AT4G05230,AT4G05240,AT4G06479,AT4G10660,AT4G10680,AT4G11340,AT4G12220,AT4G15200,AT4G15250,AT4G15650,AT4G16730,AT4G19260,AT4G19340,AT4G19800,AT4G21010,AT4G21630,AT4G26350,AT4G30030,AT4G30300,AT4G31690,AT4G32090,AT4G33840,AT4G38070,AT4G39020,AT5G01440,AT5G03580,AT5G03590,AT5G04400,AT5G05030,AT5G08141,AT5G11130,AT5G11660,AT5G12000,AT5G13130,AT5G14290,AT5G15110,AT5G16020,AT5G16090,AT5G17970,AT5G23180,AT5G23960,AT5G27310,AT5G28885,AT5G31412,AT5G35540,AT5G37320,AT5G39010,AT5G39390,AT5G39460,AT5G39470,AT5G40050,AT5G40750,AT5G41765,AT5G43390,AT5G44345,AT5G44960,AT5G47290,AT5G47300,AT5G48700,AT5G49750,AT5G52350,AT5G53040,AT5G56070,AT5G56110,AT5G56390,AT5G56400,AT5G56640,AT5G58170,AT5G59280,AT5G59810,AT5G60090,AT5G60740,AT5G61110,AT5G61700,AT5G61710,AT5G63720

AT1G76420	ANAC031	25	0	PWM3404	DAP-seq	CUC3_col	14	2.11481E-05	0.002708464	215	AT1G04450,AT1G04500,AT1G05615,AT1G06280,AT1G07850,AT1G11990,AT1G19460,AT1G20320,AT1G20730,AT1G20750,AT1G22000,AT1G23240,AT1G23520,AT1G23570,AT1G23810,AT1G26350,AT1G26797,AT1G27220,AT1G27260,AT1G27270,AT1G29580,AT1G30670,AT1G34575,AT1G35820,AT1G36510,AT1G46408,AT1G47280,AT1G47780,AT1G49800,AT1G51230,AT1G51900,AT1G52460,AT1G55570,AT1G58265,AT1G58300,AT1G58430,AT1G59630,AT1G64070,AT1G65140,AT1G65170,AT1G65740,AT1G66960,AT1G69500,AT1G70540,AT1G70960,AT1G71290,AT1G72290,AT1G72460,AT1G75940,AT1G78160,AT1G78980,AT1G80990,AT2G02890,AT2G05360,AT2G06960,AT2G10560,AT2G13620,AT2G14540,AT2G14830,AT2G17090,AT2G18460,AT2G18810,AT2G19420,AT2G20465,AT2G20625,AT2G22520,AT2G23660,AT2G24320,AT2G26850,AT2G28090,AT2G29040,AT2G29800,AT2G30290,AT2G31030,AT2G31035,AT2G32360,AT2G32370,AT2G33090,AT2G33270,AT2G33420,AT2G34270,AT2G36700,AT2G39640,AT2G40560,AT2G42730,AT2G44550,AT2G46960,AT3G01085,AT3G03405,AT3G03480,AT3G04150,AT3G06280,AT3G09510,AT3G10595,AT3G10990,AT3G11405,AT3G11480,AT3G11920,AT3G13390,AT3G15490,AT3G17500,AT3G18810,AT3G18900,AT3G20690,AT3G20980,AT3G21840,AT3G23770,AT3G23950,AT3G24220,AT3G24620,AT3G25650,AT3G26940,AT3G28190,AT3G28925,AT3G28980,AT3G32400,AT3G33494,AT3G43710,AT3G44250,AT3G44910,AT3G44920,AT3G44930,AT3G45580,AT3G45760,AT3G45800,AT3G46240,AT3G46840,AT3G47190,AT3G47760,AT3G48220,AT3G49270,AT3G49305,AT3G52330,AT3G52350,AT3G53060,AT3G56180,AT3G56670,AT3G57250,AT3G58300,AT3G58390,AT3G59030,AT3G59170,AT3G59845,AT3G60060,AT4G00540,AT4G01160,AT4G01985,AT4G03620,AT4G04510,AT4G04985,AT4G05230,AT4G05240,AT4G10660,AT4G11340,AT4G12220,AT4G13610,AT4G13885,AT4G15250,AT4G15650,AT4G16730,AT4G19260,AT4G19340,AT4G19910,AT4G21630,AT4G26350,AT4G31690,AT4G32090,AT4G33840,AT4G34940,AT4G39020,AT5G01440,AT5G03580,AT5G03590,AT5G04400,AT5G05030,AT5G11130,AT5G11940,AT5G12000,AT5G13130,AT5G14290,AT5G15480,AT5G16020,AT5G16090,AT5G17970,AT5G20310,AT5G23180,AT5G23790,AT5G23960,AT5G26130,AT5G28885,AT5G31412,AT5G37320,AT5G39010,AT5G39390,AT5G39460,AT5G39470,AT5G40750,AT5G41765,AT5G43390,AT5G44960,AT5G48700,AT5G52350,AT5G53040,AT5G55855,AT5G56110,AT5G56390,AT5G56400,AT5G56640,AT5G58170,AT5G59810,AT5G60090,AT5G60500,AT5G60830,AT5G61700,AT5G61710

AT1G65910	anac028	0	na	PWM3358	DAP-seq	ANAC028_col	15	2.96572E-05	0.00354503	214	AT1G04450,AT1G04500,AT1G07850,AT1G16980,AT1G20730,AT1G20750,AT1G20800,AT1G21950,AT1G23240,AT1G23520,AT1G23810,AT1G26350,AT1G27220,AT1G27260,AT1G28020,AT1G30670,AT1G31080,AT1G32020,AT1G32980,AT1G33020,AT1G35820,AT1G35900,AT1G47280,AT1G48010,AT1G50220,AT1G50880,AT1G51040,AT1G51900,AT1G58265,AT1G58430,AT1G58766,AT1G62080,AT1G65170,AT1G65740,AT1G66960,AT1G68960,AT1G70540,AT1G70960,AT1G72290,AT1G72460,AT1G74290,AT1G78160,AT1G78980,AT2G01240,AT2G02890,AT2G04920,AT2G05360,AT2G13620,AT2G14830,AT2G15640,AT2G16140,AT2G17090,AT2G18810,AT2G19420,AT2G20465,AT2G20625,AT2G23660,AT2G24255,AT2G24320,AT2G25370,AT2G26850,AT2G27650,AT2G28090,AT2G29040,AT2G29800,AT2G30290,AT2G30730,AT2G31030,AT2G31035,AT2G32360,AT2G33030,AT2G33090,AT2G33270,AT2G33420,AT2G34270,AT2G36700,AT2G38910,AT2G40560,AT2G40955,AT2G42730,AT2G44550,AT2G46190,AT2G46960,AT2G47280,AT3G01085,AT3G03405,AT3G03480,AT3G04150,AT3G04200,AT3G09510,AT3G10595,AT3G10990,AT3G11405,AT3G11480,AT3G13390,AT3G17500,AT3G18180,AT3G18810,AT3G18900,AT3G21170,AT3G23770,AT3G24620,AT3G25650,AT3G26880,AT3G26940,AT3G28190,AT3G28980,AT3G30770,AT3G43710,AT3G44250,AT3G44805,AT3G44910,AT3G44920,AT3G44930,AT3G44935,AT3G45580,AT3G45760,AT3G45800,AT3G46240,AT3G46840,AT3G47190,AT3G49270,AT3G49305,AT3G52350,AT3G53050,AT3G53060,AT3G56180,AT3G56670,AT3G58300,AT3G58330,AT3G58410,AT3G59160,AT3G59170,AT3G60060,AT4G00540,AT4G01160,AT4G03450,AT4G03620,AT4G04690,AT4G08097,AT4G08395,AT4G10660,AT4G11340,AT4G12220,AT4G13610,AT4G15200,AT4G15250,AT4G19260,AT4G19340,AT4G20810,AT4G21630,AT4G26350,AT4G27420,AT4G28395,AT4G30300,AT4G32090,AT4G35280,AT4G39020,AT5G01440,AT5G01680,AT5G04400,AT5G05030,AT5G08141,AT5G11130,AT5G11370,AT5G12000,AT5G12180,AT5G13130,AT5G14290,AT5G15480,AT5G16020,AT5G16090,AT5G17970,AT5G19610,AT5G22900,AT5G23180,AT5G23260,AT5G23960,AT5G27310,AT5G28237,AT5G28885,AT5G29613,AT5G31412,AT5G33898,AT5G35540,AT5G37320,AT5G39010,AT5G39390,AT5G39620,AT5G40050,AT5G41765,AT5G43390,AT5G47290,AT5G47300,AT5G48140,AT5G48700,AT5G49260,AT5G49750,AT5G52350,AT5G52610,AT5G53040,AT5G56110,AT5G56390,AT5G56400,AT5G56560,AT5G58170,AT5G59280,AT5G59810,AT5G60090,AT5G60740,AT5G60830,AT5G61110,AT5G61700,AT5G61710

AT5G13180	ANAC083	18	na	PWM3386	DAP-seq	ANAC083_col	16	2.96572E-05	0.00354503	214	AT1G04450,AT1G04500,AT1G05615,AT1G07850,AT1G08140,AT1G11990,AT1G20750,AT1G22000,AT1G23240,AT1G23520,AT1G23590,AT1G23810,AT1G25270,AT1G27260,AT1G27270,AT1G27860,AT1G28020,AT1G30670,AT1G31080,AT1G32020,AT1G34575,AT1G47280,AT1G47390,AT1G47780,AT1G48590,AT1G49800,AT1G50220,AT1G51900,AT1G52460,AT1G55070,AT1G55570,AT1G56040,AT1G58265,AT1G58300,AT1G58430,AT1G63280,AT1G65140,AT1G65170,AT1G65780,AT1G66960,AT1G68040,AT1G70540,AT1G71290,AT1G72290,AT1G72460,AT1G75940,AT1G78160,AT1G78980,AT2G05360,AT2G06020,AT2G06960,AT2G13620,AT2G14540,AT2G14830,AT2G15170,AT2G15345,AT2G17090,AT2G18460,AT2G18810,AT2G19420,AT2G20465,AT2G20625,AT2G23660,AT2G24320,AT2G26850,AT2G28090,AT2G29040,AT2G29800,AT2G30290,AT2G32360,AT2G33030,AT2G33090,AT2G33270,AT2G33420,AT2G34270,AT2G36260,AT2G36700,AT2G38910,AT2G39030,AT2G39640,AT2G40560,AT2G44070,AT2G44550,AT2G46960,AT3G01085,AT3G02440,AT3G02970,AT3G03405,AT3G03480,AT3G04150,AT3G06280,AT3G07250,AT3G09510,AT3G10595,AT3G10990,AT3G11405,AT3G11480,AT3G13390,AT3G15490,AT3G18810,AT3G18900,AT3G19070,AT3G20980,AT3G21840,AT3G23480,AT3G23770,AT3G24270,AT3G24620,AT3G26940,AT3G28190,AT3G28980,AT3G29050,AT3G43710,AT3G44250,AT3G44910,AT3G44920,AT3G44930,AT3G45580,AT3G45760,AT3G45800,AT3G46840,AT3G47190,AT3G47760,AT3G49270,AT3G49305,AT3G52350,AT3G53050,AT3G53060,AT3G53790,AT3G58280,AT3G58300,AT3G59170,AT3G60060,AT4G00540,AT4G01160,AT4G03620,AT4G04510,AT4G04690,AT4G04985,AT4G05230,AT4G05240,AT4G08395,AT4G10440,AT4G10660,AT4G10680,AT4G11340,AT4G12220,AT4G15200,AT4G15250,AT4G16730,AT4G19260,AT4G19340,AT4G19910,AT4G21240,AT4G21260,AT4G26350,AT4G28090,AT4G28395,AT4G30300,AT4G31690,AT4G32090,AT4G33840,AT4G34940,AT4G35280,AT4G39020,AT5G03580,AT5G03590,AT5G04400,AT5G05030,AT5G05150,AT5G06010,AT5G11130,AT5G12000,AT5G13130,AT5G14290,AT5G15480,AT5G16020,AT5G20310,AT5G23180,AT5G23960,AT5G24180,AT5G26130,AT5G27310,AT5G28410,AT5G28885,AT5G31412,AT5G37320,AT5G39010,AT5G39390,AT5G39460,AT5G39470,AT5G39620,AT5G41765,AT5G43390,AT5G44960,AT5G45640,AT5G47300,AT5G48140,AT5G48700,AT5G52350,AT5G53040,AT5G53780,AT5G56110,AT5G56390,AT5G56400,AT5G56560,AT5G56640,AT5G58170,AT5G59280,AT5G59810,AT5G60090,AT5G61700,AT5G61710,AT5G63720

AT1G19040	AT1G19040	19	na	PWM3397	DAP-seq	AT1G19040_col	17	4.13603E-05	0.004362291	213	AT4G28520,AT1G04450,AT1G04500,AT1G07850,AT1G08140,AT1G20730,AT1G20750,AT1G20800,AT1G21950,AT1G22000,AT1G23240,AT1G23520,AT1G23570,AT1G23810,AT1G26350,AT1G27220,AT1G27260,AT1G27270,AT1G28020,AT1G29570,AT1G29580,AT1G29870,AT1G30670,AT1G31080,AT1G31090,AT1G32020,AT1G33020,AT1G35820,AT1G35900,AT1G43320,AT1G43330,AT1G46984,AT1G47810,AT1G49800,AT1G51040,AT1G51900,AT1G52460,AT1G57906,AT1G58265,AT1G58430,AT1G65170,AT1G65740,AT1G66960,AT1G68960,AT1G70540,AT1G70960,AT1G72460,AT1G78980,AT2G01240,AT2G05360,AT2G07140,AT2G10602,AT2G12880,AT2G13620,AT2G14830,AT2G17090,AT2G18810,AT2G19420,AT2G20465,AT2G20625,AT2G23660,AT2G24255,AT2G24320,AT2G26850,AT2G28090,AT2G29040,AT2G29800,AT2G30290,AT2G31030,AT2G31035,AT2G32360,AT2G33030,AT2G33270,AT2G33420,AT2G34270,AT2G38910,AT2G40560,AT2G40955,AT2G42730,AT2G44550,AT2G45110,AT2G46190,AT3G01085,AT3G03480,AT3G04150,AT3G04200,AT3G09510,AT3G10990,AT3G11405,AT3G11480,AT3G13390,AT3G15490,AT3G18180,AT3G18810,AT3G18900,AT3G20690,AT3G21170,AT3G23770,AT3G23950,AT3G24620,AT3G25650,AT3G26390,AT3G26940,AT3G27720,AT3G28190,AT3G28925,AT3G28980,AT3G29050,AT3G43710,AT3G44250,AT3G44805,AT3G44910,AT3G44920,AT3G44930,AT3G44935,AT3G45760,AT3G45800,AT3G46240,AT3G46840,AT3G47870,AT3G49270,AT3G52350,AT3G53050,AT3G53060,AT3G53790,AT3G56180,AT3G58300,AT3G58330,AT3G59170,AT3G60060,AT4G00540,AT4G01985,AT4G03380,AT4G03620,AT4G04690,AT4G06479,AT4G08097,AT4G08395,AT4G10660,AT4G11340,AT4G12220,AT4G13610,AT4G14260,AT4G15200,AT4G19250,AT4G19260,AT4G20650,AT4G20670,AT4G20680,AT4G20810,AT4G21010,AT4G21630,AT4G26350,AT4G27420,AT4G31690,AT4G32090,AT4G38120,AT4G39020,AT4G39745,AT5G01440,AT5G01680,AT5G02730,AT5G04400,AT5G05030,AT5G08141,AT5G11130,AT5G12000,AT5G13130,AT5G15480,AT5G16020,AT5G16090,AT5G17970,AT5G19610,AT5G22900,AT5G23260,AT5G23650,AT5G23960,AT5G27310,AT5G27790,AT5G28550,AT5G28885,AT5G35540,AT5G37320,AT5G38960,AT5G39010,AT5G39390,AT5G39620,AT5G40050,AT5G41500,AT5G41765,AT5G43090,AT5G43390,AT5G44960,AT5G47290,AT5G47300,AT5G48140,AT5G49070,AT5G52350,AT5G52610,AT5G53040,AT5G56110,AT5G56400,AT5G56560,AT5G56640,AT5G58170,AT5G59810,AT5G60090,AT5G60500,AT5G60740,AT5G60830,AT5G61700,AT5G61710,AT5G62060

AT3G10500	ANAC053	9	0	PWM3370	DAP-seq	ANAC053_col	18	5.73619E-05	0.005713879	212	AT1G04450,AT1G04500,AT1G07150,AT1G07850,AT1G17150,AT1G20320,AT1G20730,AT1G20750,AT1G20800,AT1G22000,AT1G23240,AT1G23520,AT1G23610,AT1G23810,AT1G26350,AT1G27220,AT1G27260,AT1G27270,AT1G28020,AT1G29570,AT1G31080,AT1G32020,AT1G32980,AT1G33020,AT1G35820,AT1G46984,AT1G51040,AT1G51230,AT1G51900,AT1G55570,AT1G56040,AT1G57906,AT1G58430,AT1G60240,AT1G60350,AT1G61095,AT1G65170,AT1G65740,AT1G66960,AT1G68960,AT1G69100,AT1G70540,AT1G72290,AT1G74290,AT1G78980,AT2G01240,AT2G02890,AT2G13620,AT2G14830,AT2G15345,AT2G15640,AT2G17090,AT2G17750,AT2G18810,AT2G19420,AT2G20465,AT2G20625,AT2G22520,AT2G23660,AT2G28090,AT2G29040,AT2G29800,AT2G30290,AT2G31030,AT2G31035,AT2G32360,AT2G33030,AT2G33190,AT2G33270,AT2G33420,AT2G34270,AT2G40560,AT2G42730,AT2G44175,AT2G44550,AT2G46190,AT2G46960,AT3G01085,AT3G04150,AT3G09510,AT3G10990,AT3G11405,AT3G11480,AT3G12000,AT3G13390,AT3G15490,AT3G18180,AT3G18810,AT3G18900,AT3G19410,AT3G20155,AT3G21170,AT3G21880,AT3G23770,AT3G23850,AT3G24620,AT3G26880,AT3G26940,AT3G27720,AT3G28190,AT3G28980,AT3G30770,AT3G43710,AT3G44250,AT3G44800,AT3G44805,AT3G44910,AT3G44920,AT3G44930,AT3G44935,AT3G45760,AT3G45800,AT3G46840,AT3G47190,AT3G47760,AT3G48220,AT3G49270,AT3G49305,AT3G53050,AT3G53060,AT3G58280,AT3G58300,AT3G58330,AT3G59170,AT3G59180,AT3G60060,AT4G00540,AT4G03620,AT4G04690,AT4G04985,AT4G06479,AT4G08097,AT4G10680,AT4G11340,AT4G11730,AT4G12220,AT4G13610,AT4G14260,AT4G15200,AT4G15250,AT4G15650,AT4G19250,AT4G19260,AT4G19340,AT4G19800,AT4G20790,AT4G21010,AT4G21630,AT4G27420,AT4G28090,AT4G32090,AT4G35210,AT4G35820,AT4G38120,AT4G39020,AT4G39745,AT5G01440,AT5G03580,AT5G03590,AT5G04045,AT5G05030,AT5G08141,AT5G11130,AT5G12000,AT5G12180,AT5G13130,AT5G14290,AT5G16020,AT5G16090,AT5G17180,AT5G17750,AT5G17970,AT5G19610,AT5G22900,AT5G23180,AT5G23260,AT5G23960,AT5G25340,AT5G25390,AT5G27310,AT5G28190,AT5G28885,AT5G35540,AT5G37320,AT5G38960,AT5G39010,AT5G39620,AT5G40050,AT5G41765,AT5G43390,AT5G48140,AT5G48700,AT5G49260,AT5G49750,AT5G51250,AT5G52350,AT5G53040,AT5G53780,AT5G56110,AT5G56390,AT5G56400,AT5G56560,AT5G56640,AT5G59280,AT5G59810,AT5G60090,AT5G60740,AT5G60830,AT5G61110,AT5G61700,AT5G61710,AT5G67000

AT1G32870	ANAC013	11	0	PWM3351	DAP-seq	ANAC013_col	19	7.91127E-05	0.007465742	211	AT1G04450,AT1G04500,AT1G07850,AT1G20320,AT1G20730,AT1G20750,AT1G20800,AT1G22000,AT1G23240,AT1G23520,AT1G23610,AT1G23810,AT1G26350,AT1G27220,AT1G27260,AT1G27270,AT1G28020,AT1G29570,AT1G29580,AT1G29870,AT1G30670,AT1G31080,AT1G31090,AT1G32020,AT1G32980,AT1G33020,AT1G35820,AT1G42190,AT1G46408,AT1G46984,AT1G51040,AT1G51230,AT1G51900,AT1G55570,AT1G58265,AT1G58430,AT1G60240,AT1G61095,AT1G64070,AT1G65140,AT1G65170,AT1G65740,AT1G66960,AT1G68960,AT1G72460,AT1G78980,AT2G01240,AT2G02890,AT2G04910,AT2G04920,AT2G05360,AT2G10602,AT2G13150,AT2G13620,AT2G14540,AT2G17090,AT2G18810,AT2G20465,AT2G20625,AT2G22520,AT2G23660,AT2G24255,AT2G28090,AT2G29040,AT2G29800,AT2G30290,AT2G31030,AT2G31035,AT2G32360,AT2G33030,AT2G33270,AT2G33420,AT2G34270,AT2G36700,AT2G38910,AT2G39060,AT2G40560,AT2G42730,AT2G44550,AT2G46190,AT2G47280,AT3G01085,AT3G04150,AT3G06280,AT3G09110,AT3G09510,AT3G10990,AT3G11405,AT3G11480,AT3G13390,AT3G15490,AT3G17500,AT3G18180,AT3G18810,AT3G18900,AT3G21880,AT3G23770,AT3G23950,AT3G24620,AT3G25650,AT3G26880,AT3G26940,AT3G28190,AT3G28980,AT3G32400,AT3G33494,AT3G43710,AT3G44250,AT3G44910,AT3G44920,AT3G44930,AT3G44935,AT3G45760,AT3G45800,AT3G46240,AT3G46840,AT3G47870,AT3G49270,AT3G49305,AT3G52330,AT3G53050,AT3G53060,AT3G56180,AT3G58280,AT3G58300,AT3G59845,AT3G60060,AT4G00540,AT4G01985,AT4G03380,AT4G03620,AT4G04690,AT4G04985,AT4G08097,AT4G11340,AT4G11730,AT4G12220,AT4G13610,AT4G13885,AT4G14260,AT4G15200,AT4G15250,AT4G15650,AT4G21010,AT4G21630,AT4G26350,AT4G27420,AT4G30300,AT4G31690,AT4G32090,AT4G35210,AT4G38120,AT4G39020,AT4G39745,AT5G01440,AT5G01680,AT5G03580,AT5G03590,AT5G04045,AT5G04400,AT5G05030,AT5G08141,AT5G11130,AT5G12000,AT5G12180,AT5G13130,AT5G15110,AT5G16020,AT5G16090,AT5G17740,AT5G17750,AT5G17970,AT5G19610,AT5G22900,AT5G23260,AT5G23960,AT5G25340,AT5G25960,AT5G27310,AT5G28885,AT5G31412,AT5G35540,AT5G36680,AT5G38440,AT5G39010,AT5G39460,AT5G39470,AT5G39620,AT5G40050,AT5G41765,AT5G43390,AT5G48140,AT5G48700,AT5G49070,AT5G49750,AT5G52350,AT5G53040,AT5G56110,AT5G56390,AT5G56400,AT5G56560,AT5G56640,AT5G59810,AT5G60090,AT5G60500,AT5G60740,AT5G61110,AT5G61700,AT5G61710,AT5G62060,AT5G62970

AT1G79580	ANAC033	27	na	PWM3418	DAP-seq	SMB_col	20	7.91127E-05	0.007465742	211	AT1G04450,AT1G04500,AT1G05615,AT1G07850,AT1G08140,AT1G11990,AT1G17150,AT1G20750,AT1G22000,AT1G23240,AT1G23270,AT1G23520,AT1G23810,AT1G27260,AT1G27270,AT1G27860,AT1G28020,AT1G29870,AT1G30670,AT1G31080,AT1G32020,AT1G34575,AT1G44980,AT1G47280,AT1G47625,AT1G47780,AT1G48590,AT1G49800,AT1G50220,AT1G51900,AT1G52460,AT1G54880,AT1G55070,AT1G55570,AT1G56040,AT1G57906,AT1G58265,AT1G58300,AT1G58430,AT1G61095,AT1G65140,AT1G65170,AT1G66960,AT1G69470,AT1G69500,AT1G70540,AT1G70960,AT1G71290,AT1G72460,AT1G76290,AT1G77780,AT1G78160,AT1G78980,AT2G05360,AT2G06020,AT2G13620,AT2G14700,AT2G14830,AT2G15170,AT2G15640,AT2G17090,AT2G18460,AT2G18810,AT2G19420,AT2G20465,AT2G20625,AT2G21480,AT2G23660,AT2G24320,AT2G26850,AT2G28090,AT2G29040,AT2G29800,AT2G30290,AT2G32360,AT2G33030,AT2G33090,AT2G33420,AT2G34270,AT2G36700,AT2G38910,AT2G40560,AT2G42730,AT2G44070,AT2G46190,AT2G46960,AT3G02440,AT3G03405,AT3G03480,AT3G04150,AT3G06280,AT3G06630,AT3G09510,AT3G10990,AT3G11405,AT3G11480,AT3G13390,AT3G16680,AT3G18180,AT3G18810,AT3G18900,AT3G19070,AT3G21840,AT3G23480,AT3G23770,AT3G24270,AT3G24620,AT3G25650,AT3G26880,AT3G26940,AT3G28190,AT3G28980,AT3G42550,AT3G43710,AT3G44250,AT3G44910,AT3G44920,AT3G44930,AT3G45580,AT3G45760,AT3G46840,AT3G47130,AT3G47140,AT3G47190,AT3G47760,AT3G47870,AT3G49305,AT3G52350,AT3G53050,AT3G53060,AT3G53790,AT3G58330,AT3G58410,AT3G59160,AT3G59170,AT3G59845,AT3G60060,AT3G60560,AT4G00540,AT4G01160,AT4G03620,AT4G04402,AT4G04510,AT4G04690,AT4G04985,AT4G05230,AT4G05240,AT4G10440,AT4G10660,AT4G11340,AT4G15250,AT4G19250,AT4G19260,AT4G19340,AT4G19910,AT4G21240,AT4G21260,AT4G21630,AT4G26350,AT4G28090,AT4G28395,AT4G30300,AT4G31690,AT4G32090,AT4G34940,AT4G35280,AT5G01440,AT5G03580,AT5G03590,AT5G04400,AT5G05030,AT5G05150,AT5G11130,AT5G12000,AT5G12180,AT5G14290,AT5G15480,AT5G16020,AT5G20310,AT5G22380,AT5G22900,AT5G23180,AT5G23960,AT5G26130,AT5G27310,AT5G28463,AT5G36680,AT5G37320,AT5G39010,AT5G39390,AT5G39460,AT5G39470,AT5G39620,AT5G43390,AT5G44345,AT5G44760,AT5G44960,AT5G47300,AT5G48700,AT5G53040,AT5G53510,AT5G56110,AT5G56390,AT5G56400,AT5G56640,AT5G58170,AT5G59810,AT5G60090,AT5G61110,AT5G61700,AT5G61710

AT2G18060	ANAC037	18	0	PWM3424	DAP-seq	VND1_col	21	7.91127E-05	0.007465742	211	AT3G22640,AT1G04450,AT1G04500,AT1G06280,AT1G07150,AT1G07850,AT1G08140,AT1G13290,AT1G20730,AT1G20750,AT1G23240,AT1G23520,AT1G23570,AT1G23590,AT1G23610,AT1G23810,AT1G26350,AT1G26797,AT1G27080,AT1G27260,AT1G28000,AT1G28020,AT1G29870,AT1G30350,AT1G30670,AT1G31080,AT1G32020,AT1G32710,AT1G33020,AT1G35520,AT1G35820,AT1G46408,AT1G47810,AT1G50220,AT1G51040,AT1G51900,AT1G52460,AT1G53285,AT1G53815,AT1G53820,AT1G56040,AT1G58265,AT1G58430,AT1G65140,AT1G65170,AT1G66960,AT1G70540,AT1G70960,AT1G75940,AT1G78980,AT2G04920,AT2G05360,AT2G05635,AT2G06960,AT2G09970,AT2G12880,AT2G14830,AT2G16140,AT2G16220,AT2G16450,AT2G17090,AT2G19420,AT2G20465,AT2G20625,AT2G22520,AT2G23660,AT2G24255,AT2G24320,AT2G26850,AT2G28090,AT2G29800,AT2G30290,AT2G31030,AT2G31035,AT2G32360,AT2G33030,AT2G33270,AT2G33420,AT2G34270,AT2G36260,AT2G38910,AT2G40560,AT2G42730,AT2G44070,AT2G44550,AT2G45110,AT2G46960,AT3G03405,AT3G03480,AT3G04150,AT3G04200,AT3G07250,AT3G09510,AT3G10595,AT3G10990,AT3G11405,AT3G11920,AT3G13390,AT3G18180,AT3G18810,AT3G20690,AT3G23770,AT3G23950,AT3G26390,AT3G26940,AT3G27720,AT3G28120,AT3G28925,AT3G28980,AT3G30770,AT3G32120,AT3G32400,AT3G33494,AT3G43710,AT3G44250,AT3G44910,AT3G44920,AT3G44930,AT3G44935,AT3G45580,AT3G45760,AT3G45800,AT3G46840,AT3G47760,AT3G48220,AT3G49270,AT3G49450,AT3G52330,AT3G52350,AT3G53050,AT3G56670,AT3G58300,AT3G58330,AT3G58360,AT3G58410,AT3G58890,AT3G59845,AT4G00540,AT4G01160,AT4G01985,AT4G03380,AT4G03620,AT4G04690,AT4G08395,AT4G10660,AT4G11340,AT4G12220,AT4G13610,AT4G14260,AT4G15060,AT4G15650,AT4G19260,AT4G19340,AT4G21010,AT4G21630,AT4G29970,AT4G30300,AT4G31690,AT4G32090,AT4G39020,AT4G39290,AT4G39745,AT5G01440,AT5G02730,AT5G03580,AT5G03590,AT5G04400,AT5G05030,AT5G05720,AT5G08141,AT5G11080,AT5G11130,AT5G12000,AT5G13130,AT5G15110,AT5G15480,AT5G16020,AT5G16090,AT5G17970,AT5G23260,AT5G23960,AT5G26840,AT5G27310,AT5G28885,AT5G38960,AT5G39010,AT5G39390,AT5G39470,AT5G39620,AT5G41500,AT5G41765,AT5G43090,AT5G43390,AT5G44960,AT5G47290,AT5G47300,AT5G47350,AT5G52610,AT5G53040,AT5G53780,AT5G56110,AT5G56400,AT5G56560,AT5G56640,AT5G59810,AT5G60740,AT5G60830,AT5G61700,AT5G61710,AT5G62970,AT5G66990

AT3G03200	anac045	6	0	PWM3363	DAP-seq	ANAC045_col	22	0.000108505	0.008843118	210	AT1G04450,AT1G04500,AT1G07850,AT1G11990,AT1G20320,AT1G20730,AT1G20750,AT1G20800,AT1G22000,AT1G23240,AT1G23520,AT1G23810,AT1G26350,AT1G27220,AT1G27260,AT1G27270,AT1G29570,AT1G29580,AT1G30670,AT1G31270,AT1G32020,AT1G32980,AT1G35820,AT1G46408,AT1G47280,AT1G51040,AT1G51230,AT1G51900,AT1G52460,AT1G56040,AT1G58265,AT1G58430,AT1G61095,AT1G65170,AT1G65740,AT1G66960,AT1G68960,AT1G70540,AT1G72290,AT1G72460,AT1G74220,AT1G75940,AT1G78160,AT1G78980,AT2G04910,AT2G04920,AT2G05360,AT2G13620,AT2G14390,AT2G14830,AT2G17090,AT2G18810,AT2G19420,AT2G20465,AT2G20625,AT2G23660,AT2G24320,AT2G26850,AT2G28090,AT2G29040,AT2G29800,AT2G30290,AT2G32360,AT2G33030,AT2G33090,AT2G33270,AT2G33420,AT2G34270,AT2G36700,AT2G40560,AT2G42730,AT2G44550,AT2G46960,AT3G01085,AT3G03405,AT3G03480,AT3G04150,AT3G06280,AT3G09510,AT3G10990,AT3G11405,AT3G11480,AT3G13390,AT3G15490,AT3G16680,AT3G17500,AT3G18180,AT3G18810,AT3G18900,AT3G19410,AT3G23770,AT3G24220,AT3G24620,AT3G25650,AT3G26880,AT3G26940,AT3G27810,AT3G28190,AT3G28980,AT3G30770,AT3G32400,AT3G33494,AT3G43710,AT3G44250,AT3G44910,AT3G44920,AT3G44930,AT3G44935,AT3G45760,AT3G45800,AT3G46840,AT3G47190,AT3G49270,AT3G49305,AT3G52330,AT3G52350,AT3G53050,AT3G53060,AT3G56180,AT3G56670,AT3G58300,AT3G58410,AT3G58890,AT3G59845,AT3G60060,AT4G00540,AT4G01160,AT4G03620,AT4G04690,AT4G04985,AT4G05240,AT4G08097,AT4G08395,AT4G08430,AT4G09190,AT4G10680,AT4G11340,AT4G12220,AT4G13610,AT4G13885,AT4G15200,AT4G15250,AT4G15650,AT4G19260,AT4G19340,AT4G21630,AT4G26350,AT4G27420,AT4G28090,AT4G28395,AT4G30300,AT4G31690,AT4G32090,AT4G33840,AT4G38120,AT4G39020,AT4G39745,AT5G01440,AT5G03580,AT5G03590,AT5G04400,AT5G05030,AT5G08141,AT5G11130,AT5G12000,AT5G12180,AT5G13130,AT5G14290,AT5G15110,AT5G16020,AT5G16090,AT5G17740,AT5G17750,AT5G17970,AT5G19610,AT5G20310,AT5G22900,AT5G23960,AT5G27310,AT5G28885,AT5G31412,AT5G35540,AT5G37320,AT5G39010,AT5G39390,AT5G39460,AT5G39470,AT5G39620,AT5G40050,AT5G41765,AT5G43390,AT5G44760,AT5G44960,AT5G48700,AT5G49260,AT5G52350,AT5G53040,AT5G56070,AT5G56110,AT5G56390,AT5G56400,AT5G56640,AT5G59280,AT5G59810,AT5G60090,AT5G60740,AT5G60830,AT5G61110,AT5G61700,AT5G61710

AT1G02250	anac005	4	na	PWM3349	DAP-seq	ANAC005_col	23	0.000147987	0.01153655	209	AT2G15010,AT1G04450,AT1G04500,AT1G05800,AT1G16980,AT1G17150,AT1G20320,AT1G20730,AT1G20750,AT1G23420,AT1G23520,AT1G27260,AT1G27270,AT1G29570,AT1G29870,AT1G30670,AT1G31080,AT1G31090,AT1G32020,AT1G32980,AT1G33020,AT1G34410,AT1G35520,AT1G35820,AT1G46984,AT1G47625,AT1G50220,AT1G51230,AT1G51900,AT1G52970,AT1G53420,AT1G53815,AT1G53820,AT1G53940,AT1G55570,AT1G58430,AT1G58766,AT1G60410,AT1G63070,AT1G65170,AT1G65740,AT1G66960,AT1G78160,AT1G78220,AT1G78980,AT2G01780,AT2G02890,AT2G04920,AT2G05360,AT2G06645,AT2G07020,AT2G07140,AT2G13620,AT2G14830,AT2G15640,AT2G15740,AT2G16140,AT2G17090,AT2G18460,AT2G18810,AT2G20465,AT2G20625,AT2G22520,AT2G23660,AT2G24320,AT2G24340,AT2G25370,AT2G26850,AT2G27650,AT2G28090,AT2G29040,AT2G29800,AT2G30290,AT2G31030,AT2G31035,AT2G32360,AT2G33030,AT2G33270,AT2G33420,AT2G40560,AT2G40955,AT2G42730,AT2G44550,AT2G46190,AT2G46960,AT3G02440,AT3G03480,AT3G04150,AT3G09510,AT3G10990,AT3G11405,AT3G11480,AT3G11920,AT3G18180,AT3G18810,AT3G18900,AT3G21170,AT3G22030,AT3G23770,AT3G24620,AT3G25650,AT3G26880,AT3G26940,AT3G27785,AT3G28190,AT3G28780,AT3G28980,AT3G30770,AT3G42786,AT3G43710,AT3G44250,AT3G44800,AT3G44920,AT3G44930,AT3G45580,AT3G45800,AT3G46840,AT3G47190,AT3G47760,AT3G47870,AT3G49270,AT3G49305,AT3G52330,AT3G52970,AT3G53050,AT3G53060,AT3G56180,AT3G58280,AT3G58330,AT3G58410,AT3G58890,AT3G59160,AT3G59170,AT3G60060,AT4G00540,AT4G01245,AT4G03380,AT4G03450,AT4G04690,AT4G04985,AT4G09860,AT4G11340,AT4G13610,AT4G14260,AT4G15053,AT4G15250,AT4G19250,AT4G19260,AT4G19340,AT4G19800,AT4G21260,AT4G21630,AT4G23370,AT4G26350,AT4G27420,AT4G28090,AT4G29460,AT4G29970,AT4G30300,AT4G32090,AT4G38120,AT4G39753,AT5G01440,AT5G04400,AT5G11130,AT5G12000,AT5G12180,AT5G13130,AT5G15110,AT5G16020,AT5G16090,AT5G17180,AT5G17970,AT5G19610,AT5G20310,AT5G22900,AT5G23180,AT5G23260,AT5G23960,AT5G25340,AT5G27310,AT5G28885,AT5G31412,AT5G35830,AT5G36680,AT5G37320,AT5G39010,AT5G39200,AT5G39620,AT5G39630,AT5G40050,AT5G41500,AT5G43090,AT5G43390,AT5G44345,AT5G44760,AT5G46380,AT5G48140,AT5G48700,AT5G53040,AT5G56060,AT5G56110,AT5G56390,AT5G56400,AT5G56640,AT5G59280,AT5G59810,AT5G60090,AT5G61690

AT3G17730	anac057	8	na	PWM3373	DAP-seq	ANAC057_col	24	0.00036304	0.027122108	206	AT5G45690,AT1G04450,AT1G04500,AT1G07150,AT1G07850,AT1G08140,AT1G09245,AT1G11990,AT1G20730,AT1G20750,AT1G22000,AT1G23240,AT1G23520,AT1G23570,AT1G23810,AT1G26350,AT1G26797,AT1G27220,AT1G27260,AT1G27270,AT1G28000,AT1G28020,AT1G29870,AT1G30670,AT1G31080,AT1G32020,AT1G33020,AT1G34575,AT1G35900,AT1G43320,AT1G43330,AT1G47280,AT1G48010,AT1G51040,AT1G51230,AT1G51900,AT1G52460,AT1G56040,AT1G58430,AT1G58766,AT1G65140,AT1G65170,AT1G65740,AT1G66960,AT1G70540,AT1G72290,AT1G72460,AT1G74290,AT1G78980,AT2G01800,AT2G04920,AT2G05360,AT2G10602,AT2G12880,AT2G13150,AT2G13620,AT2G14540,AT2G14830,AT2G16220,AT2G17090,AT2G18810,AT2G19420,AT2G20465,AT2G20625,AT2G22520,AT2G24320,AT2G26850,AT2G28090,AT2G29040,AT2G29800,AT2G30290,AT2G31030,AT2G31035,AT2G32360,AT2G33030,AT2G33090,AT2G33270,AT2G33420,AT2G34270,AT2G36260,AT2G36700,AT2G38910,AT2G40560,AT2G42730,AT2G44550,AT3G01085,AT3G03405,AT3G03480,AT3G04150,AT3G07250,AT3G09510,AT3G10595,AT3G10990,AT3G11405,AT3G11480,AT3G13390,AT3G15490,AT3G17500,AT3G18810,AT3G18900,AT3G19410,AT3G20155,AT3G23770,AT3G23950,AT3G24620,AT3G25650,AT3G26940,AT3G27720,AT3G28190,AT3G32120,AT3G32400,AT3G43710,AT3G44250,AT3G44805,AT3G44910,AT3G44920,AT3G44930,AT3G44935,AT3G45760,AT3G45800,AT3G46840,AT3G47760,AT3G49270,AT3G52330,AT3G52350,AT3G53050,AT3G56670,AT3G58300,AT3G58330,AT3G58890,AT3G59845,AT3G60060,AT4G00540,AT4G01160,AT4G03620,AT4G04402,AT4G04690,AT4G05240,AT4G10660,AT4G11340,AT4G12220,AT4G13610,AT4G13885,AT4G14260,AT4G15250,AT4G15650,AT4G19260,AT4G21010,AT4G21630,AT4G26350,AT4G29970,AT4G31690,AT4G32090,AT4G39020,AT4G39745,AT5G01140,AT5G01440,AT5G03580,AT5G03590,AT5G04400,AT5G05030,AT5G05150,AT5G08141,AT5G11130,AT5G12000,AT5G13130,AT5G14290,AT5G16020,AT5G16090,AT5G17970,AT5G20310,AT5G22900,AT5G23960,AT5G27310,AT5G28237,AT5G28885,AT5G31412,AT5G37320,AT5G38960,AT5G39010,AT5G39460,AT5G39470,AT5G39620,AT5G39770,AT5G40750,AT5G41765,AT5G43390,AT5G47290,AT5G47300,AT5G48140,AT5G48700,AT5G49070,AT5G49260,AT5G52350,AT5G52610,AT5G53040,AT5G56110,AT5G56390,AT5G56400,AT5G56560,AT5G56640,AT5G59810,AT5G60090,AT5G60830,AT5G61700,AT5G61710

AT4G10350	ANAC070	24	na	PWM3379	DAP-seq	ANAC070_col	25	0.000484157	0.034723758	205	AT1G04450,AT1G04500,AT1G05615,AT1G07850,AT1G08140,AT1G11990,AT1G17150,AT1G20750,AT1G22000,AT1G23240,AT1G23520,AT1G25270,AT1G27260,AT1G27270,AT1G27570,AT1G27860,AT1G29870,AT1G30670,AT1G31080,AT1G32020,AT1G34575,AT1G44980,AT1G47280,AT1G47625,AT1G47780,AT1G48590,AT1G49800,AT1G50220,AT1G51900,AT1G52460,AT1G54880,AT1G55070,AT1G55570,AT1G56040,AT1G57906,AT1G58265,AT1G58300,AT1G58430,AT1G61095,AT1G65140,AT1G65170,AT1G66960,AT1G69500,AT1G70540,AT1G71290,AT1G72460,AT1G76290,AT1G77780,AT1G78160,AT1G78980,AT2G05360,AT2G06020,AT2G13620,AT2G14700,AT2G14830,AT2G15170,AT2G15640,AT2G17090,AT2G17960,AT2G18460,AT2G18810,AT2G19420,AT2G20465,AT2G24320,AT2G26850,AT2G27650,AT2G28090,AT2G29040,AT2G29800,AT2G30290,AT2G32360,AT2G33030,AT2G33090,AT2G33420,AT2G34270,AT2G36700,AT2G38910,AT2G40560,AT2G42730,AT2G44070,AT2G46190,AT2G46960,AT3G02970,AT3G03405,AT3G03480,AT3G04150,AT3G06280,AT3G06630,AT3G09510,AT3G10990,AT3G11405,AT3G11480,AT3G13390,AT3G15490,AT3G18180,AT3G18810,AT3G18900,AT3G19070,AT3G20760,AT3G20980,AT3G21840,AT3G23480,AT3G23770,AT3G24270,AT3G24620,AT3G25650,AT3G26880,AT3G26940,AT3G28190,AT3G28980,AT3G29050,AT3G31900,AT3G43710,AT3G43750,AT3G44250,AT3G44910,AT3G44920,AT3G44930,AT3G45580,AT3G45760,AT3G46840,AT3G47130,AT3G47140,AT3G47190,AT3G47760,AT3G47870,AT3G49305,AT3G52350,AT3G53050,AT3G53060,AT3G53790,AT3G58330,AT3G58410,AT3G59160,AT3G59170,AT3G60060,AT3G60560,AT4G00540,AT4G01160,AT4G03620,AT4G04402,AT4G04510,AT4G04690,AT4G04985,AT4G05230,AT4G05240,AT4G10440,AT4G10660,AT4G11340,AT4G15250,AT4G16730,AT4G19260,AT4G19340,AT4G19910,AT4G21240,AT4G21260,AT4G21630,AT4G26350,AT4G28090,AT4G28395,AT4G30300,AT4G31690,AT4G34940,AT4G35280,AT5G01440,AT5G03580,AT5G03590,AT5G04400,AT5G05030,AT5G05150,AT5G06010,AT5G11130,AT5G12000,AT5G12180,AT5G13130,AT5G14290,AT5G15480,AT5G16020,AT5G20310,AT5G22380,AT5G22900,AT5G23180,AT5G23960,AT5G26130,AT5G27310,AT5G36680,AT5G37320,AT5G39010,AT5G39390,AT5G39470,AT5G43390,AT5G44960,AT5G47300,AT5G48700,AT5G53040,AT5G53780,AT5G56110,AT5G56390,AT5G56400,AT5G56640,AT5G58170,AT5G59810,AT5G60090,AT5G61700,AT5G61710

Number	TF	Background_bind	Query_all	Query_bind	p_value	q_value	Gene Model Description
1	AT1G54330	926	1282	92	2.23E-13	8.70E-11	NAC domain containing protein 20;(source:Araport11)
2	AT5G07680	756	1282	80	2.90E-13	8.70E-11	NAC domain containing protein 80;(source:Araport11)
3	AT5G18270	701	1282	74	2.43E-12	4.07E-10	NAC domain containing protein 87;(source:Araport11)
4	AT1G32510	1013	1282	95	2.72E-12	4.07E-10	NAC domain containing protein 11;(source:Araport11)
5	AT3G10490	1019	1282	95	3.81E-12	4.56E-10	Encodes a NAC transcription factor that physically associates with the histone H3K4 demethylase JMJ14 and through that association is involved in transcriptional repression and flowering time control.
6	AT3G04060	728	1282	74	1.51E-11	1.51E-09	NAC046 is a member of the NAC domain containing family of transcription factors. It was identified in a screen for regulators of chlorophyll protein gene expression. Mutants in NAC046 have delayed senescence and increased CHL content suggesting a role in regulation of senescence and chlorophyll degradation.
7	AT1G79580	751	1282	73	1.63E-10	1.40E-08	NAC-domain protein. Involved in root cap development. Involved in a regulatory feedback loop with FEZ. FEZ activates SMB in hte root cap daughter cells soon after division, and SMB in turn represses FEZ expression in these cells, thereby preventing further stem cell divisions.
8	AT4G10350	604	1282	58	1.63E-08	1.22E-06	NAC domain protein. SMB, BRN1, and BRN2 act to regulate root cap maturation, in a partially redundant fashion.BRN1 and BRN2, control the cell wall maturation processes that are required to detach root cap layers from the root.
9	AT5G64060	835	1282	72	3.58E-08	2.38E-06	NAC domain containing protein 103;(source:Araport11)
10	AT5G62380	718	1282	62	2.73E-07	1.64E-05	Encodes a NAC-domain transcription factor involved in xylem formation. Induces transdifferentiation of various cells into metaxylem vessel elements. Located in the nucleus. Expression induced in the presence of auxin, cytokinin and brassinosteroids.
11	AT5G46590	1072	1282	83	4.00E-07	2.18E-05	NAC domain containing protein 96;(source:Araport11)
12	AT3G18400	688	1282	58	1.37E-06	6.33E-05	NAC domain containing protein 58;(source:Araport11)
13	AT1G32870	1088	1282	82	1.37E-06	6.33E-05	Expression in rosette leaves is activated by high concentration of boron.
14	AT3G15510	1103	1282	82	2.35E-06	1.00E-04	Note of caution: not to be confused with another protein (AtNAC6 locus AT5G39610) which on occasion has also been referred to as AtNAC2.
15	AT1G65910	1005	1282	74	9.61E-06	3.84E-04	NAC domain containing protein 28;(source:Araport11)
16	AT1G02250	736	1282	58	1.10E-05	4.10E-04	Encodes a member of the NAC family of transcription factors. ANAC005 contains sequences specifying both nuclear and plasma membrane targeting. Overexpression results in increased xylem differentiation suggesting ANAC005 promotes xylem formation.
17	AT3G15170	835	1282	62	3.56E-05	1.25E-03	Encodes a transcription factor involved in shoot apical meristem formation and cotyledon separation. Functions redundantly with CUC2 and CUC3. The cuc1 cuc2 double mutant phenotype is first detectable at the heart stage, as embryos lacking two distinct bulges of cotyledonary primordia.In post embryonic development it plays a role in axillary meristem formation, boundary separation, gynoecium and ovule development.Contains a MIR164 binding site.
18	AT5G13180	520	1282	43	4.01E-05	1.34E-03	Encodes a NAC domain transcription factor that interacts with VND7 and negatively regulates xylem vessel formation.

19	AT5G53950	824	1282	61	4.43E-05	1.40E-03	Transcriptional activator of the NAC gene family, with CUC1 redundantly required for embryonic apical meristem formation, cotyledon separation and expression of STM. Proper timing of CUC2 expression is required to maintain the phyllotactic pattern initiated in the meristem. CUC2 expression in leaf sinus region is required for serration and the extent of serration is modulated by mir164A mediated repression of CUC2. Together with CUC3-DA1-UBP15 part of a regulatory module which controls the initiation of axillary meristems, thereby determining plant architecture. Regulates the axillary meristem initiation, directly binding to the DA1 promoter.
20	AT1G19040	852	1282	62	6.39E-05	1.91E-03	NAC (No Apical Meristem) domain transcriptional regulator superfamily protein;(source:Araport11)
21	AT3G03200	1079	1282	74	9.93E-05	2.83E-03	NAC domain containing protein 45;(source:Araport11)
22	AT1G28300	487	1282	39	1.66E-04	4.51E-03	Transcription factor that contains a B3 domain, a DNA-binding motif unique to plants and characteristic of several transcription factors. Plays critical roles both early and late during embryo development. LEC2 RNA accumulates primarily during seed development. LEC2 is required for the maintenance of suspensor morphology, specification of cotyledon identity, progression through the maturation phase, and suppression of premature germination. It establishes a cellular environment sufficient to initiate embryo development - ectopic, postembryonic expression of LEC2 in transgenic plants induces the formation of somatic embryos and other organ-like structures and often confers embryonic characteristics to seedlings and to reproductive and vegetative organs of mature plants.
23	AT1G34190	919	1282	64	1.76E-04	4.58E-03	Encodes a NAC domain transcription factor that regulates the mitochondrial retrograde response and coordinates organellar functions and stress responses.
24	AT2G46770	458	1282	37	1.95E-04	4.86E-03	NAC transcription factor NST1. NST1 and NST2 are redundant in regulating secondary wall thickening in anther walls and siliques. An NST1 promoter fusion was detected in various tissues in which lignified secondary walls develop. Both MYC2 and MYC4 bind to the NST1 promoter and appear to regulate its expression in response to blue light.
25	AT3G10480	813	1282	57	3.10E-04	7.27E-03	Encodes a NAC transcription factor that physically associates with the histone H3K4 demethylase JMJ14 and through that association is involved in transcriptional repression and flowering time control. It binds the NAC-binding site, the Mitochondrial Dysfunction Motif.
26	AT5G66300	867	1282	60	3.16E-04	7.27E-03	Encodes a NAC-domain transcription factor. Expressed in the vascular tissue.
27	AT3G10500	1088	1282	72	3.47E-04	7.71E-03	Encodes a transcriptional activator that is associated with the plasma membrane in a dormant form and is proteolytically cleaved to create a form that can enter the nucleus. It is thought to promote ROS production by binding directly to the promoters of genes encoding ROS biosynthetic enzymes during drought-induced leaf senescence. The mRNA is cell-to-cell mobile.
28	AT2G28550	285	1282	25	4.68E-04	1.00E-02	AP2 family transcription factor that is involved in regulation of flowering and innate immunity.Interacts with CRY2 to regulate CO and FT. TOE1 binds to activation domain of CO and binds CORE sequences of the FT promoter.TOE1/TOE2 are also targets of MiR172b and function in regulation of innate immunity.
29	AT3G17730	730	1282	51	6.42E-04	1.33E-02	NAC domain containing protein 57;(source:Araport11)

30	AT3G54990	251	1282	22	9.10E-04	1.82E-02	Encodes a AP2 domain transcription factor that can repress flowering. SMZ and its paralogous gene, SNARCHZAPFEN (SNZ), share a signature with partial complementarity to the miR172 microRNA, whose precursor is induced upon flowering.
31	AT4G17980	1035	1282	67	9.49E-04	1.83E-02	Encodes ANAC071, a transcription factor involved in cell proliferation in incised inflorescence stems.
32	AT1G34180	841	1282	56	1.18E-03	2.21E-02	NAC domain containing protein 16;(source:Araport11)
33	AT2G24430	635	1282	44	1.59E-03	2.88E-02	NAC domain containing protein 38;(source:Araport11)
34	AT4G01550	712	1282	48	1.85E-03	3.27E-02	Encodes a plasma-membrane bound NAC transcription factor, whose controlled proteolytic activation allows it to enter the nucleus.
35	AT1G76420	750	1282	50	1.95E-03	3.33E-02	Identified in an enhancer trap line; member of the NAC family of proteins. Expressed at the boundary between the shoot meristem and lateral organs and the polar nuclei in the embryo sac. Together with CUC2-DA1-UBP15 part of a regulatory module which controls the initiation of axillary meristems, thereby determining plant architecture. Regulates axillary meristem initiation by directly binding to the DA1 promoter.
36	AT4G01540	458	1282	33	2.71E-03	4.51E-02	Encodes a membrane-bound NAC (for NAM, ATAF1/2, CUC2) transcription factor, designated NTM1 (for NAC with transmembrane motif1). NTM1 regulates cell division in Arabidopsis.
37	AT1G51140	617	1282	40	7.46E-03	1.21E-01	Encodes a basic helix-loop-helix-type transcription factor involved in photoperiodism flowering. Binds to the E-box cis-element in the CONSTANS (CO) promoter to regulate flowering. Interacts with CFL1 and along with CFLAP2 negatively regulates cuticle development. Binds to the potassium channel gene KAT1 as a dimer. The DNA-binding capacity is inhibited in response to ABA through phosphorylation-dependent monomerization.
38	AT2G27300	907	1282	55	8.97E-03	1.41E-01	NTL8 is a membrane-associated NAC transcription factor that binds both TRY and TCL1. Overexpression results in fewer trichomes.
39	AT3G49530	463	1282	31	9.86E-03	1.51E-01	Transcription factor that serves as a molecular link between cold signals and pathogen resistance responses. Undergoes proteolytic processing triggered by cold-induced changes in membrane fluidity.It relocates from the plasma membrane to the nucleus in response to ER stress. NAC062 is phosphorylated by SnRK2.8 at Thr-142.
40	AT3G26790	631	1282	40	1.07E-02	1.59E-01	Transcriptional factor with high similarity to the B3 region of the VP1/ABI3-like proteins. Full length FUS3 protein binds to the highly conserved RY motif [DNA motif CATGCA(TG)], present in many seed-specific promoters, and the B3 domains of this transcription factor is necessary for the specific interaction with the RY element. Transcriptional activity of FUS3 requires the B3 DNA-binding domain and an activation domain. FUS3 specifies cotyledon identity. Regulator of gene expression during late embryogenesis. Involved in the control foliar organ identity in Arabidopsis by regulating the synthesis of two hormones, abscisic acid and gibberellin. FUS3 together with LEC1 positively regulate the abundance of the ABI3 protein in the seed.
41	AT4G21080	468	1282	31	1.14E-02	1.67E-01	Dof-type zinc finger domain-containing protein;(source:Araport11)
42	AT4G36160	624	1282	39	1.41E-02	2.02E-01	Encodes a NAC-domain transcription factor that is expressed in developing xylem. Over expression of this protein causes ectopic secondary cell wall growth. Complements some of the cell wall defects seen in SND1/NST1 double mutants.

43	AT2G18060	663	1282	41	1.46E-02	2.03E-01	Encodes a NAC-domain transcription factor that is expressed in developing vessels and protoxylem. Along with other members of this family, VND1 appears to regulate the development of genes required for secondary cell wall biosynthesis.
44	AT4G18770	299	1282	21	1.52E-02	2.08E-01	MYB98 is a member of the R2R3-MYB gene family, the members of which likely encode transcription factors. Within an ovule, MYB98 is expressed exclusively in the synergid cells, and mutations in this gene affect the female gametophyte specifically. myb98 female gametophytes are affected in two unique features of the synergid cell, pollen tube guidance and the filiform apparatus, but are otherwise normal. This suggests that MYB98 controls the development of specific features within the synergid cell during female gametophyte development. MYB98 also is expressed in trichomes and endosperm. Homozygous myb98 mutants exhibit no sporophytic defects, including trichome and endosperm defects.
45	AT1G13260	603	1282	37	2.09E-02	2.78E-01	Encodes an AP2/B3 domain transcription factor which is upregulated in response to low temperature. It contains a B3 DNA binding domain. It has circadian regulation and may function as a negative growth regulator. The mRNA is cell-to-cell mobile.
46	AT1G32240	310	1282	21	2.20E-02	2.87E-01	Encodes a member of the KANADI family of putative transcription factors. Together with KAN1, this gene appears to be involved in the development of the carpel and the outer integument of the ovule.Along with KAN1 and KAN4 appears to regulate the proper localization of PIN1 in early embryogenesis.
47	AT5G39610	648	1282	39	2.43E-02	3.10E-01	Encodes a NAC-domain transcription factor. Positively regulates aging-induced cell death and senescence in leaves. This gene is upregulated in response to salt stress in wildtype as well as NTHK1 transgenic lines although in the latter case the induction was drastically reduced. It was also upregulated by ABA, ACC and NAA treatment, although in the latter two cases, the induction occurred relatively late when compared with NaCl or ABA treatments. Note: this protein (AtNAC6) on occasion has also been referred to as AtNAC2, not to be confused with the AtNAC2 found at locus AT3G15510.
48	AT1G15720	111	1282	9	2.65E-02	3.25E-01	Arabidopsis thaliana myb family transcription factor (At1g15720). MYB/SANT domain containing protein.
49	AT1G28470	807	1282	47	2.66E-02	3.25E-01	NAC domain containing protein 10;(source:Araport11)
50	AT5G67580	1841	1282	98	2.96E-02	3.55E-01	Encodes a telomeric DNA binding protein and Single Myb Histone (SMH) gene family member. In vitro, the protein preferentially binds double-stranded telomeric repeats, but it can also bind to the single G-rich telomeric strand.

51	AT3G24650	234	1282	16	3.35E-02	3.93E-01	Homologous to the maize transcription factor Viviparous-1. Full length ABI3 protein binds to the highly conserved RY motif [DNA motif CATGCA(TG)], present in many seed-specific promoters, and the B3 domains of this transcription factor is necessary for the specific interaction with the RY element. Transcriptional activity of ABI3 requires the B3 DNA-binding domain and an activation domain. In addition to the known N-terminal-located activation domain, a second transcription activation domain was found in the B1 region of ABI3. ABI3 is essential for seed maturation. Regulator of the transition between embryo maturation and early seedling development. Putative seed-specific transcriptional activator. ABI3 is a central regulator in ABA signaling and is unstable in vivo. It interacts with and can by polyubiquitinated by AIP2 in vivo. Based on double mutant analyses, ABI3 interacts genetically with both FUS3 and LEC1 and is involved in controlling accumulation of chlorophyll and anthocyanins, sensitivity to abscisic acid, and expression of the members of the 12S storage protein gene family. In addition, both FUS3 and LEC1 regulate positively the abundance of the ABI3 protein in the seed. Alternative splicing of ABI3 is developmentally regulated by SUA (AT3G54230).
52	AT3G61120	824	1282	47	3.62E-02	4.17E-01	Encodes AGL13, a member of the AGL6 clade of the MADS-box gene family. Expressed in both pollen and ovules. Functions in male and female gametophyte morphogenesis.
53	AT5G13790	791	1282	45	4.05E-02	4.58E-01	AGL15 (AGAMOUS-Like 15) is a member of the MADS domain family of regulatory factors. Although AGL15 is preferentially expressed during embryogenesis, AGL15 is also expressed in leaf primordia, shoot apical meristems and young floral buds, suggesting that AGL15 may play a role during post-germinative development. Transgenic plants that ectopically express AGL15 show delays in the transition to flowering, perianth abscission and senescence and fruit and seed maturation. Role in embryogenesis and gibberellic acid catabolism. Targets B3 domain transcription factors that are key regulators of embryogenesis.AGL15 binds the HAE promoter in floral receptacles and represses HAE expression. AGL15 is phosphorylated in a MKK4/5 dependent manner in floral receptacles. Serines 231 and 257 are phosphorylated in floral receptacles. AGL15 also directly regulates the expression of the peroxidase PRX17, linking it to lignified tissue expression.
54	AT4G21040	429	1282	26	4.62E-02	5.12E-01	Dof-type zinc finger domain-containing protein;(source:Araport11)
55	AT1G32640	75	1282	6	4.94E-02	5.28E-01	Encodes a MYC-related transcriptional activator with a typical DNA binding domain of a basic helix-loop-helix leucine zipper motif. Binds to an extended G-Box promoter motif and interacts with Jasmonate ZIM-domain proteins. MYC2 interacts with EIN3 and EIL1 to repress hook curvature and resistance to Botrytis cinera.Its transcription is induced by dehydration stress, ABA treatment and blue light via CRY1. Negative regulator of blue light-mediated photomorphogenic growth and blue and far-red-light-regulated gene expression. Positive regulator of lateral root formation. Regulates diverse JA-dependent functions. Negatively regulates Trp metabolism and biosynthesis of Trp-derived secondary metabolites. Positively regulates flavonoid biosynthesis, resistance to insects, and response to oxidative stress. Regulates other transcription factors, and negatively regulates its own expression. For example it binds to and regulates the expression of NST1. Its stability is modulated by PUB10 through polyubiquitination.
56	AT4G16430	75	1282	6	4.94E-02	5.28E-01	bHLH3 interacts with JAZ proteins, and functions redundantly with bHLH13, bHLH14 and bHLH17 to negatively regulate jasmonate responses.
